# Identification and characterization of ferroptosis-related genes in therapy-resistant gastric cancer

**DOI:** 10.1097/MD.0000000000038193

**Published:** 2024-05-17

**Authors:** Jieli Yu, Hua Li, Can Huang, Huoguo Chen

**Affiliations:** a Department of Geriatric Oncology, Jiangxi Cancer Hospital, Nanchang, China; b Department of Oncology, Pengze County People’s Hospital, Jiujiang, China.

**Keywords:** drug resistance, ferroptosis, gastric cancer, risk model, survival, tumor microenvironment

## Abstract

Therapy resistance in gastric cancer poses ongoing challenges, necessitating the identification of ferroptosis-related genes linked to overall survival for potential therapeutic insights. The purpose of the study was to identify ferroptosis-related genes contributing to therapy resistance in gastric cancer and explore their associations with overall survival. Differentially expressed ferroptosis-related genes were identified in therapy-resistant versus therapy-responsive gastric cancer patients. Hub genes were selected from these genes. Enrichment analysis focused on oxidative stress and ROS metabolism. Validation was conducted in a TCGA stomach adenocarcinoma dataset. A hub gene-based risk model (DUSP1/TNF/NOX4/LONP1) was constructed and assessed for overall survival prediction. Associations with the tumor immune microenvironment were examined using the ESTIMATE algorithm and correlation analysis. Ten hub genes were identified, enriched in oxidative stress and ROS metabolism. Validation confirmed their aberrant expressions in the TCGA dataset. The hub gene-based risk model effectively predicted overall survival. High G6PD/TNF expression and low NOX4/SREBF1/MAPK3/DUSP1/KRAS/SIRT3/LONP1 expression correlated with stromal and immune scores. KRAS/TNF/MAPK3 expression positively correlated with immune-related SREBF1/NOX4 expression. DUSP1/NOX4/SREBF1/TNF/KRAS expression was associated with immune cell infiltration. The hub gene-based risk model (DUSP1/TNF/NOX4/LONP1) shows promise as an overall survival predictor in gastric cancer. Ferroptosis-related hub genes represent potential therapeutic targets for overcoming therapy resistance in gastric cancer treatment.

## 1. Introduction

Gastric cancer ranks the fifth most common cancer and the third cancer-related death worldwide.^[1]^ Surgical resection of the lesion remains the primary treatment for gastric cancer. However, most patients present with advanced unresectable disease at diagnosis, and approximately 50% relapse after radical surgery.^[2]^ Chemotherapy is considered the first-line treatment for advanced or recurrent gastric cancer and is applied perioperatively in resectable gastric cancer.^[3,4]^ Unfortunately, intrinsic or acquired drug resistance may lead to treatment failure in these patients, representing a major challenge in gastric cancer therapy.^[5]^

Apoptosis is often deactivated in therapy-resistant gastric cancer cells.^[6]^ Ferroptosis is an iron-dependent cell death that is different from apoptosis, necrosis, and autophagy, representing a novel approach to reversing resistance to chemotherapy, targeted therapy, and immunotherapy in cancer treatment.^[7,8]^ Ferroptosis is caused by iron-dependent lipid peroxidation and subsequent plasma membrane rupture, characterized by intracellular accumulation of Fe^2+^, lipid peroxide, and reactive oxygen species (ROS).^[9]^ Recent studies have implicated ferroptosis activation in overcoming therapy resistance in cancer, and many genes that are involved in lipid peroxidation and amino acid metabolism have been identified to play important roles in the regulation of ferroptosis, such as NADPH oxidases (NOXs) that are the major sources of cellular ROS and glutathione peroxidase 4 (GPX4) that catalyzes the reduction of lipid peroxides.^[9,10]^ In gastric cancer, inducing ferroptosis by restraining Nrf2/Keap1/xCT signaling can reverse cisplatin resistance.^[11]^ Downregulation of microRNA-522 and subsequent upregulation of arachidonate lipoxygenase 15 expression enhances cisplatin/paclitaxel sensitivity of gastric cancer cells by inducing ferroptosis.^[12]^ These findings suggest that ferroptosis-related genes are highly involved in therapy resistance in gastric cancer. Although a few studies have identified differentially expressed ferroptosis-related genes in gastric cancer, such as tumor necrosis factor (TNF), dual specificity phosphatase 1 (DUSP1), and mitochondrial lon peptidase 1 (LONP1),^[13–15]^ ferroptosis-related genes involved in therapy resistance in gastric cancer and their associations with the prognosis of patients remain unclear.

In this study, we aimed to identify ferroptosis-related genes that may contribute to therapy resistance in gastric cancer and explored their associations with the overall survival of patients. Our study provides several potential therapeutic targets for overcoming therapy resistance in gastric cancer and constructs a potential ferroptosis-related gene signature for predicting the overall survival of patients with gastric cancer.

## 2. Methods

### 2.1. Data collection

The RNA-sequencing data of this study were from the NCBI Gene Expression Omnibus (GEO) under accession numbers GSE31811 and GSE26253 using the GEOquery package.^[16]^ The raw data were normalized using log transformation. A comparison of data distributions before and after normalization was visualized using box plots. Principal component analysis (PCA) was performed to compare data separations before and after normalization using the R packages FactoMineR and factoextra.^[17]^ The PCA results were plotted using the R package scatterplot3d.

### 2.2. Identification of differentially expressed ferroptosis-related genes

GSE31811 and GSE26253 datasets were separated into therapy-responsive and therapy-resistant groups, respectively. Differential gene expression was analyzed using the R package limma.^[18]^ The Benjamini-Hochberg method was used to control the false discovery rate (FDR) and adjust the *P* value. Genes with (|log2FC|) > 0.5 and adjusted *P* value < .05 were considered differentially expressed genes (DEGs). Volcano plots and heat maps were generated using R package ggplot2.^[19]^ Ferroptosis-related genes were obtained from GeneCards (http://www.genecards.org/)^[20]^ and FerrDB,^[21]^ respectively. For GeneCards searching, we used “ferroptosis” as the keyword and the association score > 1 as the threshold. Intersection analysis was performed between DEGs and ferroptosis-related genes to identify ferroptosis-related DEGs.

### 2.3. Identification and functional characterization of hub genes

To identify hub genes related to ferroptosis in gastric cancer, a protein-protein interaction (PPI) network was established in the 21 ferroptosis-related DEGs using String (https://www.string-db.org/).^[22]^ The interaction degrees of the genes were analyzed using Cytoscape plugin cytoHubba.^[23]^ The top 10 DEGs with the highest interaction degrees were defined as hub genes. The potential interacting microRNAs and transcription factors were predicted using miRNet.^[24]^ The potential interacting RNA binding proteins and drugs were predicted using StarBase^[25]^ (http://starbase.sysu.edu.cn/) and RNAactDrug,^[26]^ respectively.

Functional and pathway enrichment analyses were conducted using Gene Ontology (GO),^[27]^ Kyoto Encyclopedia of Genes and Genomes (KEGG),^[28]^ Gene set enrichment analysis (GSEA),^[29]^ and Gene Set Variation Analysis (GSVA).^[30,31]^ The results of GO and KEGG were analyzed using clusterprofiler^[32]^ and visualized using GOplot.^[33]^ For GSEA, the reference gene sets “c5.go.v7.4.entrez.gmt” and “c2.cp.kegg.v7.4.entrez.gmt” were downloaded from the MSigDB database.^[30]^ Enrichment analysis was conducted using lusterProfiler. GSVA was performed using the R package “GSVA.”^[30,31]^ The reference gene set “h.all.v7.4.symbols.gmt” was obtained from MSigDB. The pathway with an adjusted *P* value < .05 was considered statistically significant.

### 2.4. Establishment of a hub gene-based risk model

To establish a hub gene-based risk model to predict the overall survival of patients with gastric cancer, the mRNA expression profiles and clinical data of The Cancer Genome Atlas stomach adenocarcinoma dataset (TCGA-STAD) were downloaded from UCSC xena (http://xena.ucsc.edu/). A total of 381 patients with survival data were included in the study. The multivariate Cox hazard regression analysis was performed to identify potential prognostic factors in the hub genes. A forest map of the factors was generated. The predictive values of the risk model on 1, 3, and 5-year survivals were evaluated using the receiver operating characteristic curve and the area under the curve. The risk score was calculated as ∑ coefficient × expression level of the gene to categorize the patients into low-risk and high-risk groups based on the cutoff value calculated by “survival.” The correlations of the risk scores with the age, gender, clinical grading, and tumor staging were analyzed using the Wilcoxon rank sum test. Decision curve analysis (DCA) was conducted to assess the performance of the risk model in predicting the overall survival of the patients using “stdca.”^[34]^ A nomogram of the risk model was generated.

### 2.5. Estimation of STromal and Immune cells in MAlignant Tumours using Expression data (ESTIMATE)

The immune and stromal scores of each sample in the TCGA-STAD were obtained using “estimate.”^[35]^ The correlation of hub gene expression with ESTIMATE scores was analyzed. The relative proportions of 22 types of infiltrating immune cells were calculated using CIBERSORT.^[36]^ The correlations of hub gene expression with the proportions of 22 types of infiltrating immune cells were assessed using the Pearson correlation coefficient. Immune-related genes were obtained from ImmPort (https://www.immport.org)^[37]^ to analyze the correlation of hub genes with immune-related genes.

### 2.6. Statistical analysis

Statistical analysis was performed using the R program 4.0 (https://www.r-project.org/). Multiple testing correction was performed using Benjamini-Hochberg procedure. FDR correction was performed across multiple testing to reduce false-positive rates. Comparisons between two groups were conducted using the Student *t* test. The difference between non-normally distributed variables was analyzed by the Wilcoxon rank-sum test. A *P* value < .05 was considered statistically significant.

## 3. Results

### 3.1. Identification of ferroptosis-related DEGs in therapy-resistant gastric cancer

To identify ferroptosis-related DEGs in therapy-resistant gastric cancer, we downloaded the RNA-sequencing data of the GSE31811 and GSE26253 datasets. As shown in Table 1, GSE31811 contained the RNA-sequencing data of 21 gastric tumor samples (13 therapy-responsive and 8 therapy-resistant) and 17 normal gastric mucosa samples from 19 gastric patients. GSE26253 contained the RNA-sequencing data of 432 gastric tumor samples (255 therapy-responsive and 177 therapy-resistant) from gastric cancer patients who were at high risk after curative surgery plus adjuvant chemoradiotherapy.

**Table 1:** Datasets.

	GSE31811	GSE26253	TCGA
Organism	Homo sapiens	Homo sapiens	Homo sapiens
Experiment type	Expression profiling by array	Expression profiling by array	Expression profiling by array
Platforms	GPL6480	GPL8432	–
Normal sample (n)	17	–	31 (with OS)
Tumor sample (n)	21	432	350 (with OS)
Chemoresponsive (n)	13	255	–
Chemoresistant (n)	8	177	–

OS = overall survival, TCGA = The Cancer Genome Atlas.

**Figure 1.**
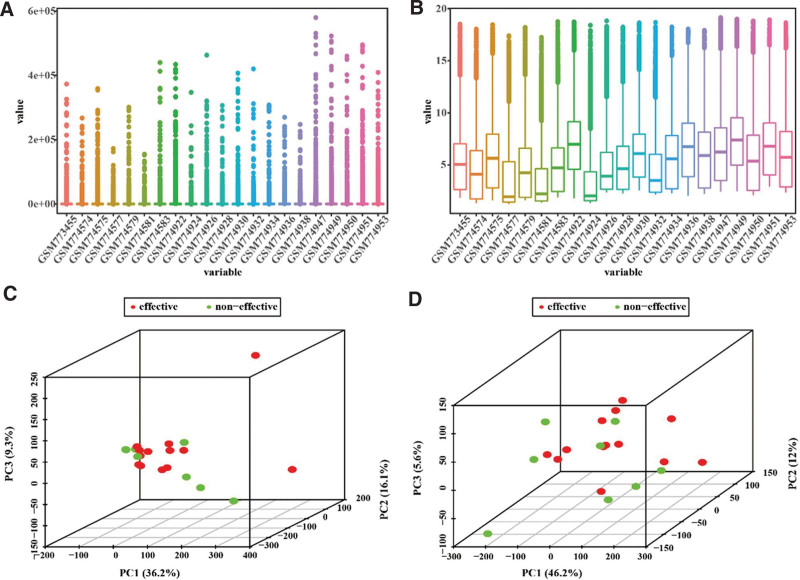
Data preprocessing of the GSE31811 dataset. The RNA-sequencing data of the GSE31811 dataset were obtained from the NCBI Gene Expression Omnibus and normalized using logarithm transformation. (A, B) Box plots were generated to compare data distributions before (A) and after (B) normalization. (C, D) PCA was performed to compare data separations before (C) and after (D) normalization. PCA = principal component analysis.

To decrease intragroup variation for the following analysis, we normalized the raw data using logarithm transformation. The values after normalization were more centered than those before normalization in GSE31811 (Fig. 1A and B). PCA results showed better separation between therapy-responsive and therapy-resistant groups after data normalization (Fig. 1C and D; PC1: 46.2% vs 36.2%). Although there was no significant difference in data distribution or group separation before and after data preprocessing of the GSE26253 dataset (Figure S1, Supplemental Digital Content, http://links.lww.com/MD/M514), our results still highlight the importance of normalizing gene sequencing data.

**Figure 2.**
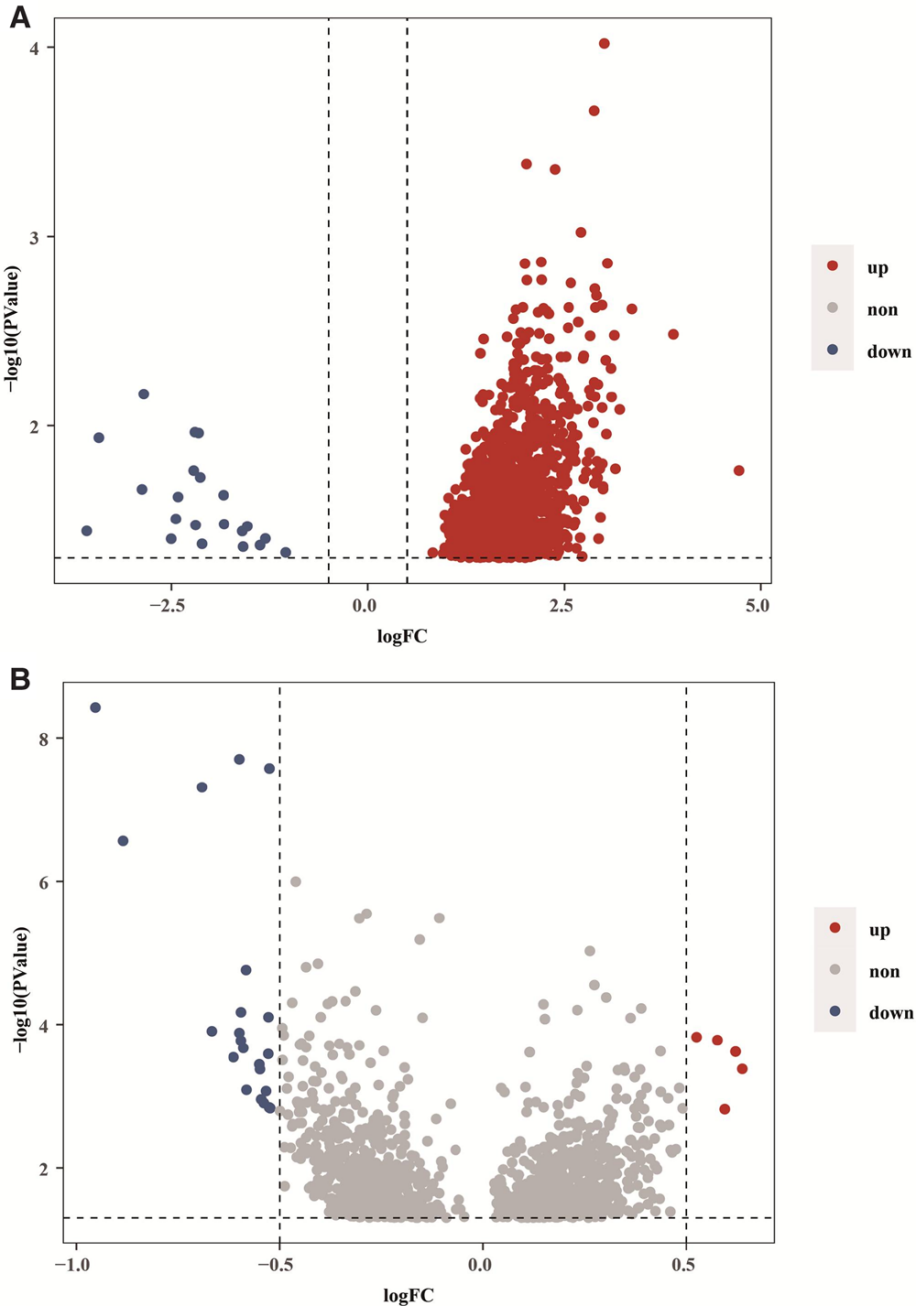
Identification of DEGs between therapy-responsive and therapy-resistant patients with gastric cancer. GSE31811 (A, B) and GSE26253 (C, D) datasets were divided into therapy-responsive and therapy-resistant groups, respectively. Volcano plots (A, C) and heatmaps (B, D) depict the differential RNA expressions in therapy-resistant group versus therapy-responsive group. Genes with |fold change| > 1 and adjusted *P* value < .05 were defined as DEGs. The red dots represent upregulated DEGs. The blue dots represent downregulated DEGs. DEGs = differentially expressed genes.

**Figure 3.**
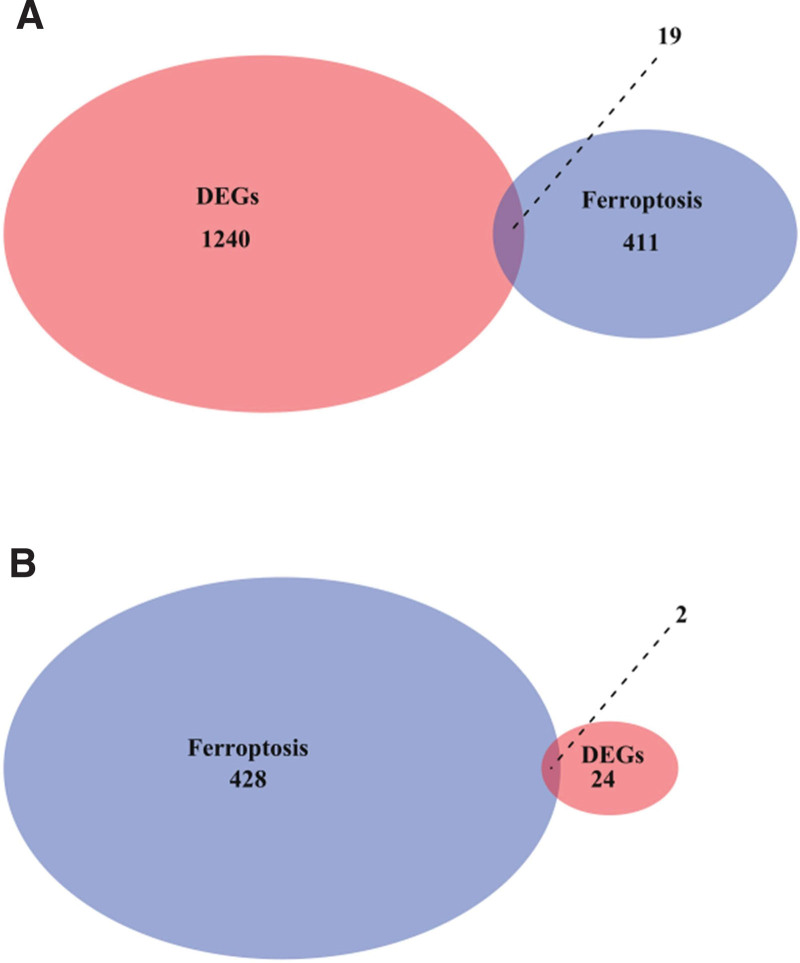
Identification of ferroptosis-related DEGs. A total of 430 ferroptosis-related genes were obtained from GeneCards and FerrDB databases after removing the duplicates. Intersection analysis was conducted to identify ferroptosis-related DEGs in GSE31811 (A) and GSE26253 (B) datasets. DEGs = differentially expressed genes.

Then, we sought to identify the DEGs between therapy-responsive and therapy-resistant groups. With (|log2FC|) > 1 and adjusted *P* value < .05 as the threshold, we identified 1259 DEGs in GSE31811 dataset (Fig. 2A) and 26 DEGs in GSE26253 dataset (Fig. 2B). Further, we obtained 430 ferroptosis-related genes after removing the duplicates from 156 ferroptosis-related genes in the GeneCards database and 335 ferroptosis-related genes in the FerrDB database. Intersection analysis revealed 21 ferroptosis-related DEGs (Fig. 3A and B). These genes are potential ferroptosis-related genes involved in therapy resistance in gastric cancer.

### 3.2. Ten hub genes are identified in the PPI network

**Figure 4.**
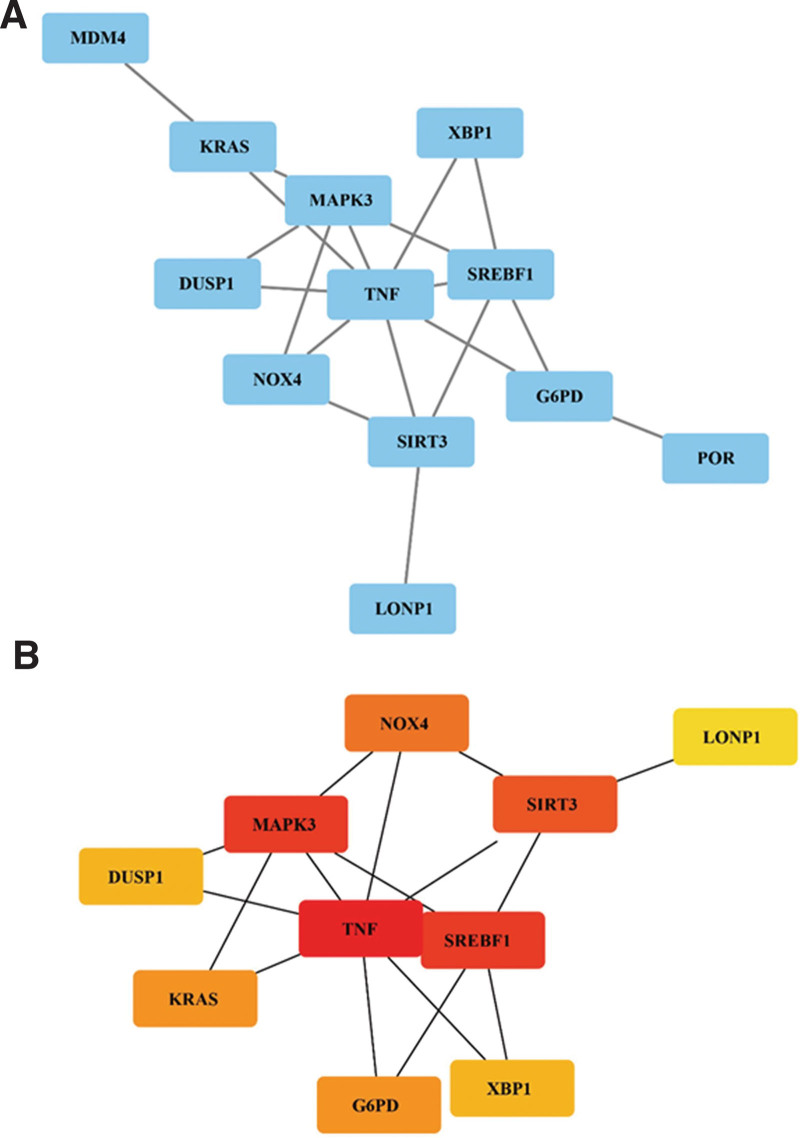
Identification of hub genes. (A) A protein-protein interaction network was constructed to identify the hub genes in 21 ferroptosis-related DEGs using String. (B) The top 10 DEGs with the highest interaction degrees were defined as hub genes. DEGs = differentially expressed genes.

To identify the hub genes in the 21 ferroptosis-related DEGs, we established a PPI network (Fig. 4A). CytoHubba analysis revealed the top 10 genes with the highest interaction degrees (Fig. 4B), including TNF (interaction degree = 16), MAPK3 (interaction degree = 8), SREBF1 (interaction degree = 8), SIRT3 (interaction degree = 5), NOX4 (interaction degree = 4), G6PD (interaction degree = 3), KRAS (interaction degree = 3), DUSP1 (interaction degree = 2), XBP1 (interaction degree = 2), and LONP1 (interaction degree = 1).

### 3.3. The hub genes are highly related to oxidative stress, ROS and lipid metabolism, as well as immune regulation

**Figure 5.**
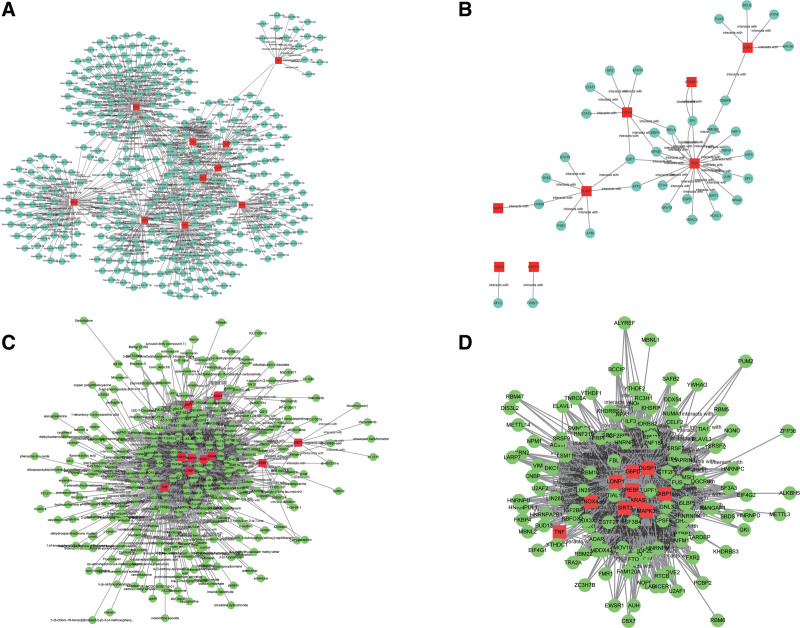
Construction of regulatory networks of the hub genes. (A) The RNA-microRNA interaction network. (B) The RNA-transcription interaction network. (C) The RNA-RNA binding protein interaction network. (D) The RNA-drug interaction network.

**Figure 6.**
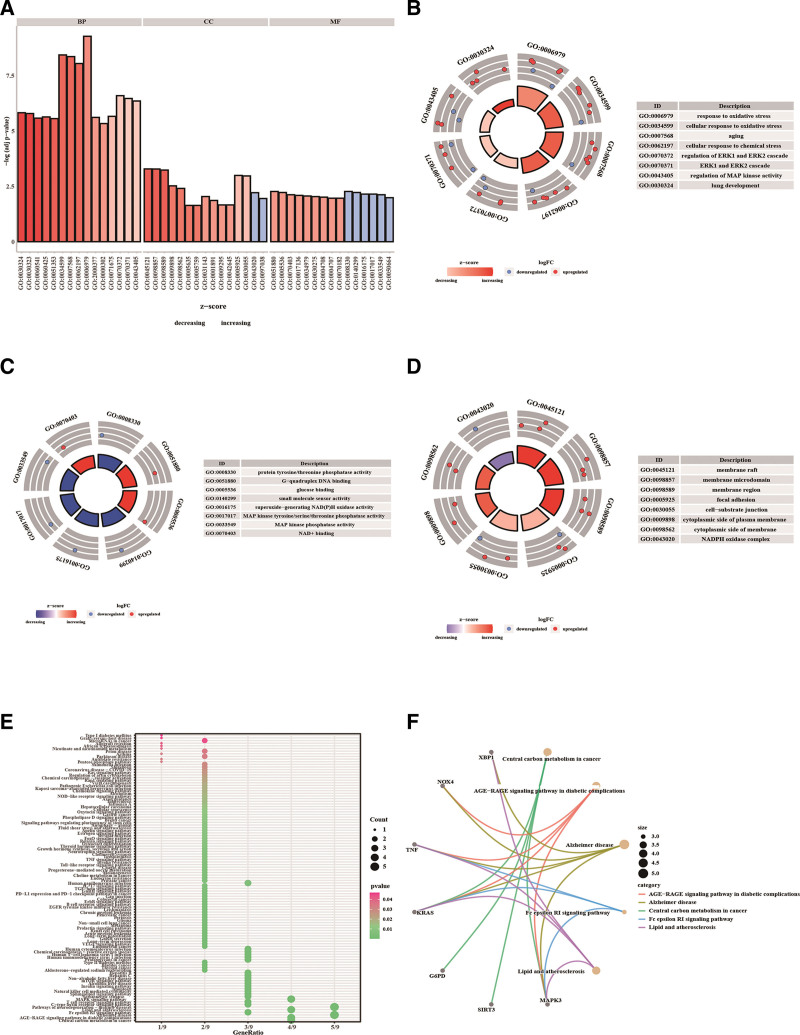
GO annotation and KEGG enrichment analysis of the hub genes. (A) GO enrichment bar chart. The y-axis represents the significance of the terms. The bars are sorted according to the z-scores. (B–D) Circle diagrams of enriched GO terms in biological process (B), molecular function (C), and cellular component (D) categories. The outer circle shows a scatter plot of the expression level (logFC) of each gene in the assigned GO term. Red dots represent upregulated genes. Blue dots represent downregulated genes. The inner ring is a bar plot indicating the significance of the GO terms, and the color corresponds to the z-score. (E) A bar chart of enriched KEGG signaling pathways. (F) The top 5 enriched KEGG signaling pathways. BP = biological process, CC = cellular component, GO = gene ontology, KEGG = Kyoto Encyclopedia of Genes and Genomes, MF = molecular function.

To characterize the functions of the hub genes, we performed GO annotation and pathway enrichment analyses. Figure 5A–D demonstrates the potential interacting microRNAs, transcription factors, RNA binding proteins, and drugs of the hub genes. GO annotation showed that the hub genes were primarily enriched in terms related to oxidative stress and ROS metabolism, such as “response to oxidative stress,” “cellular response to chemical stress,” and “regulation of reactive oxygen species metabolic process” (Fig. 6A–D; Table S1, Supplemental Digital Content, http://links.lww.com/MD/M515). We noticed that most of the enriched GO terms were upregulated in therapy-resistant patients compared with those in therapy-responsive patients, based on the Z-scores (Fig. 6A). This finding suggests that chemoresistance of gastric cancer is associated with ferroptosis. KEGG enrichment analysis revealed that the hub genes were highly related to immune regulation, lipid metabolism, and cancer progression, such as “lipid and atherosclerosis,” “T cell receptor signaling pathway,” and “MAPK signaling pathway” (Fig. 6E and F; Table S2, Supplemental Digital Content, http://links.lww.com/MD/M516).

**Figure 7.**
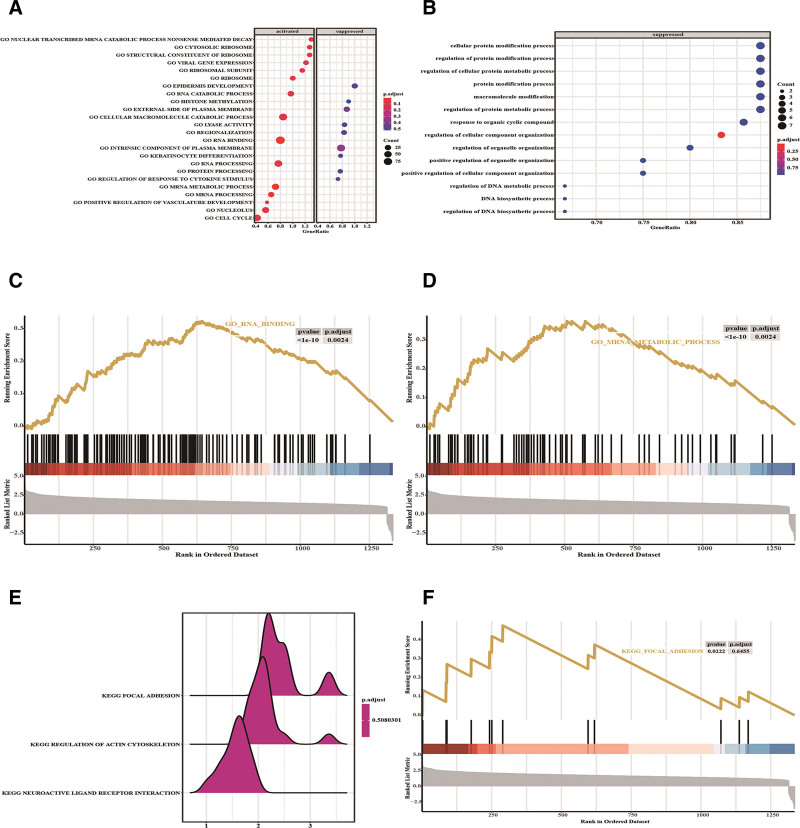
GSEA. The enriched gene sets in the GSE31811 dataset. (B) The enriched gene sets in the GSE26253 dataset. (C, D, F) Enrichment analysis of GO terms RNA binding (C) and mRNA metabolic process (D) as well as KEGG pathway focal adhesions (F). (E) Enrichment analysis of KEGG pathways focal adhesion, regulation of actin cytoskeleton, and neuroactive ligand receptor interaction. GO = gene ontology, GSEA = gene set enrichment analysis, KEGG = Kyoto Encyclopedia of Genes and Genomes.

In GSEA, based on the enrichment scores, the top 5 pathways enriched in GSE31811 were cytosolic ribosome, structural constituent of ribosome, nuclear transcribed mRNA catabolic process nonsense mediated decay, positive regulation of vasculature development, and ribosomal subunit, all of which were upregulated pathways (Fig. 7A and C–E; Table S3, Supplemental Digital Content, http://links.lww.com/MD/M517). The top 5 pathways enriched in GSE26253 were regulation of DNA biosynthetic process, DNA biosynthetic process, regulation of DNA metabolic process, positive regulation of cellular component organization, and positive regulation of organelle organization, all of which were suppressed pathways (Fig. 7B and F; Table 2). No significant results were observed in GSVA.

**Table 2:** Gene set enrichment analysis of the GSE26253 dataset.

Description	EnrichmentScore	Adjusted *P* value	*q* values
Regulation of cellular component organization	−1	2.55E-07	1.19E-10
Regulation of DNA metabolic process	−1	.958012015	0.009374219
DNA biosynthetic process	−1	.958012015	0.009374219
Regulation of DNA biosynthetic process	−1	.958012015	0.009374219
Regulation of organelle organization	−1	.958012015	0.012165249
Response to organic cyclic compound	−0.793308038	.958012015	0.026702872
Positive regulation of organelle organization	−0.857142857	.958012015	0.042416526
Positive regulation of cellular component organization	−0.857142857	.958012015	0.042416526
Cellular protein modification process	−0.821660593	.958012015	0.048432141
Regulation of protein modification process	−0.821660593	.958012015	0.048432141
Regulation of cellular protein metabolic process	−0.821660593	.958012015	0.048432141
Protein modification process	−0.821660593	.958012015	0.048432141
Macromolecule modification	−0.821660593	.958012015	0.048432141
Regulation of protein metabolic process	−0.821660593	.958012015	0.048432141

### 3.4. A risk model consisting of DUSP1, TNF, NOX4, and LONP1 can predict the overall survival of patients with gastric cancer

**Figure 8.**
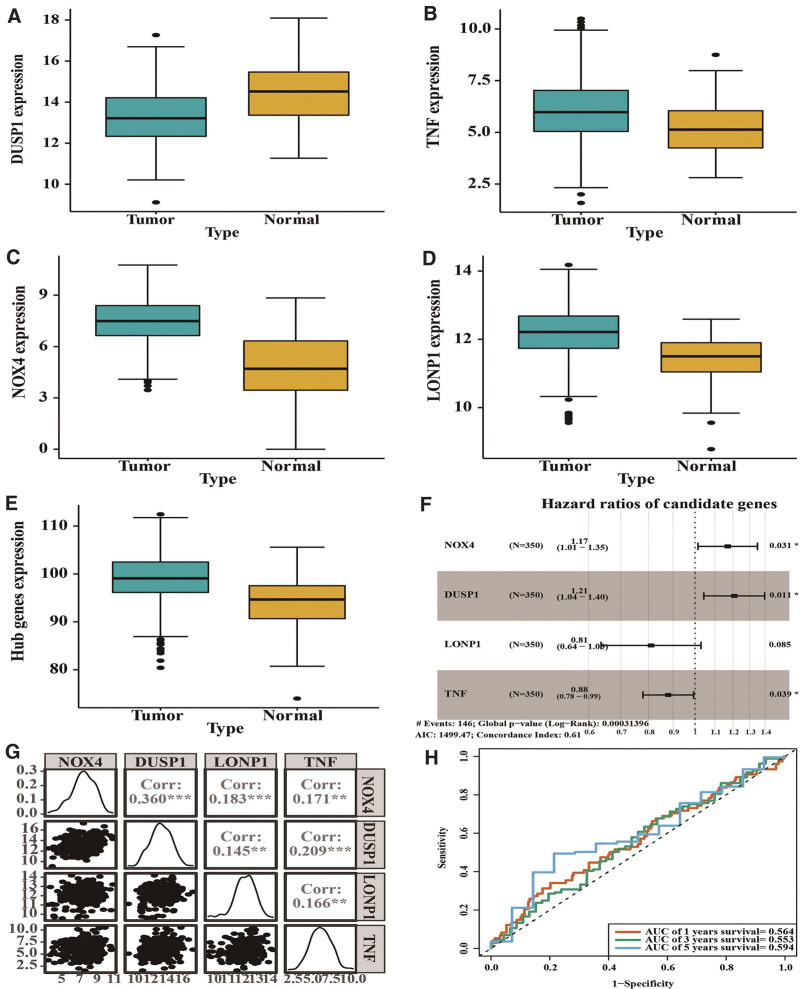
The predictive value of the hub gene mRNA expressions in the overall survival of TCGA-STAD. (A–D) Comparison of mRNA expression of DUSP1 (A), TNF (B), NOX4 (C), and LONP1 (D) between gastric tumor tissue and normal gastric tissue samples from the TCGA-STAD (n = 350). (E) Total mRNA expression of 10 hub genes. (F) Forest plot of the multivariate Cox regression analysis of potential overall survival predictors. (G) The correlation matrix of the potential predictors. (H) Time-dependent receiver operating characteristic curve analysis. AUC = area under the curve, TCGA-STAD = The Cancer Genome Atlas stomach adenocarcinoma dataset.

To investigate the clinical significance of the hub genes, we analyzed the correlation of the hub gene expression with the overall survival of patients with gastric cancer. After downloading the RNA expression profiles and clinical data of the TCGA-STAD, we obtained the survival data of 381 patients and tumoral hub gene expression levels of 350 patients. As shown in Figure 8A–D, DUSP1 expression was decreased in tumor tissue whereas TNF, NOX4, and LONP1 expressions were increased in tumor tissue, compared with those in normal tissue. The total mRNA level of the 10 hub genes was elevated in gastric tumor tissue compared with that in normal tissue (Fig. 8E). Multivariable Cox regression analysis showed that DUSP1, TNF, NOX4, and LONP1 were independent predictors of the overall survival of patients (Fig. 8F). The correlation matrix of the 4 predictors was shown in Figure 8G. Further, we constructed a risk model using the 4 predictors. The receiver operating characteristic curves showed that the area under the curves of the risk model in predicting 1, 3, and 5-year overall survivals were 0.564, 0.553, and 0.594, respectively, suggesting that the model performed well in predicting the overall survival of patients with gastric cancer (Fig. 8H).

**Figure 9.**
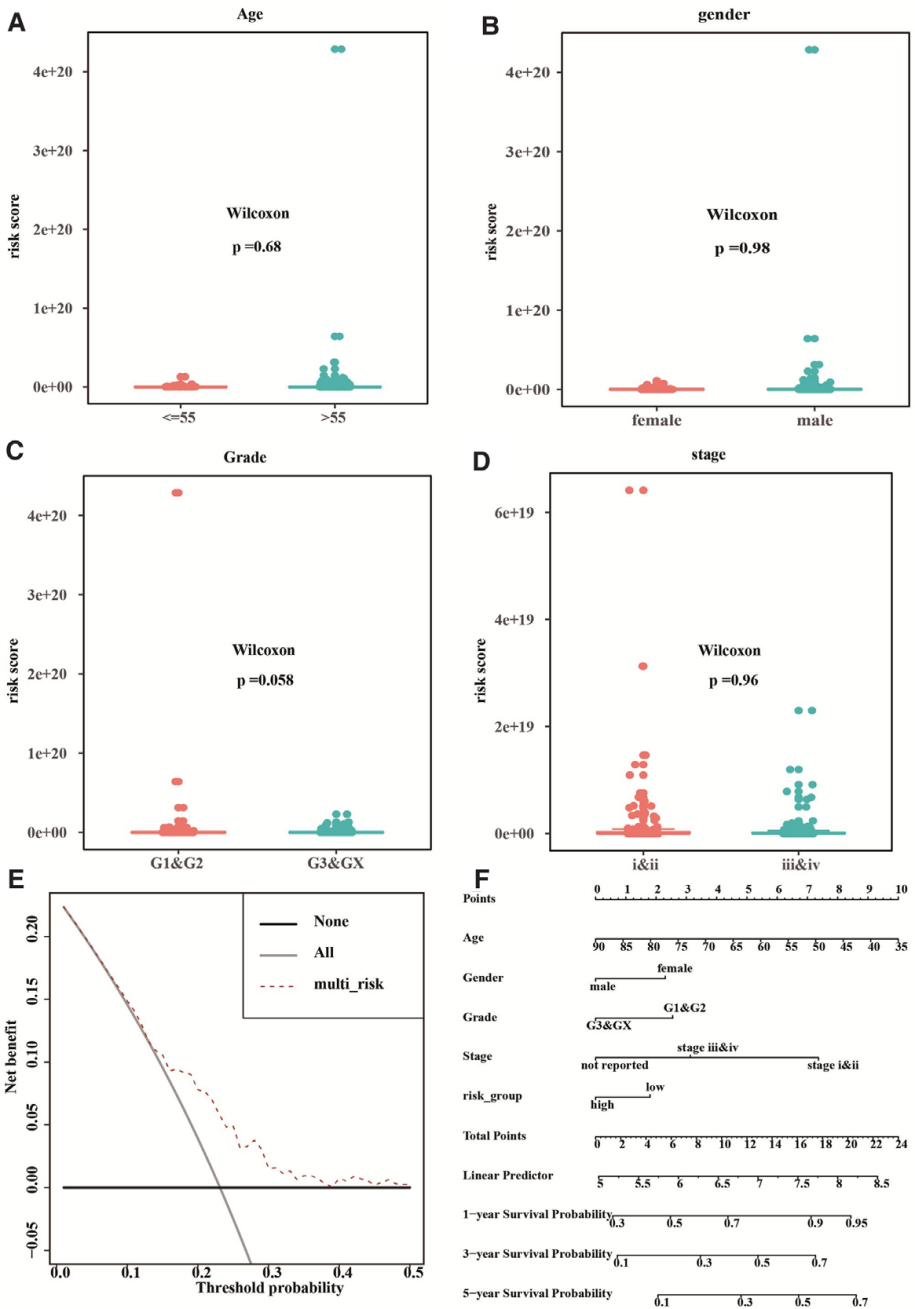
The performance of hub gene-based risk model in predicting the overall survival of patients in TCGA-STAD. (A–D) Wilcoxon rank-sum test was carried out to assess the correlation of hub gene-based risk scores with the age (A), gender (B), clinical staging (C), and tumor grading (D) of the TCGA-STAD. (E) Decision curve analysis. (F) A nomogram of the risk model. TCGA-STAD = The Cancer Genome Atlas stomach adenocarcinoma dataset.

Then, we divided the TCGA cohort into low-risk and high-risk groups according to the risk scores. Wilcoxon rank-sum test showed that the risk score was not significantly correlated with age, gender, tumor grading, and tumor staging (all *P* > .05; Figure 9A–D). DCA showed that the risk model performed better than “treat none” or “treat all” in predicting the overall survival of TCGA patients (Fig. 9E). A nomogram based on the risk scores to predict the 1, 3, and 5-year overall survival of the TCGA cohort was illustrated in Figure 9F.

### 3.5. The expressions of ferroptosis-related hub genes are associated with the immune microenvironment of gastric tumors

**Figure 10.**
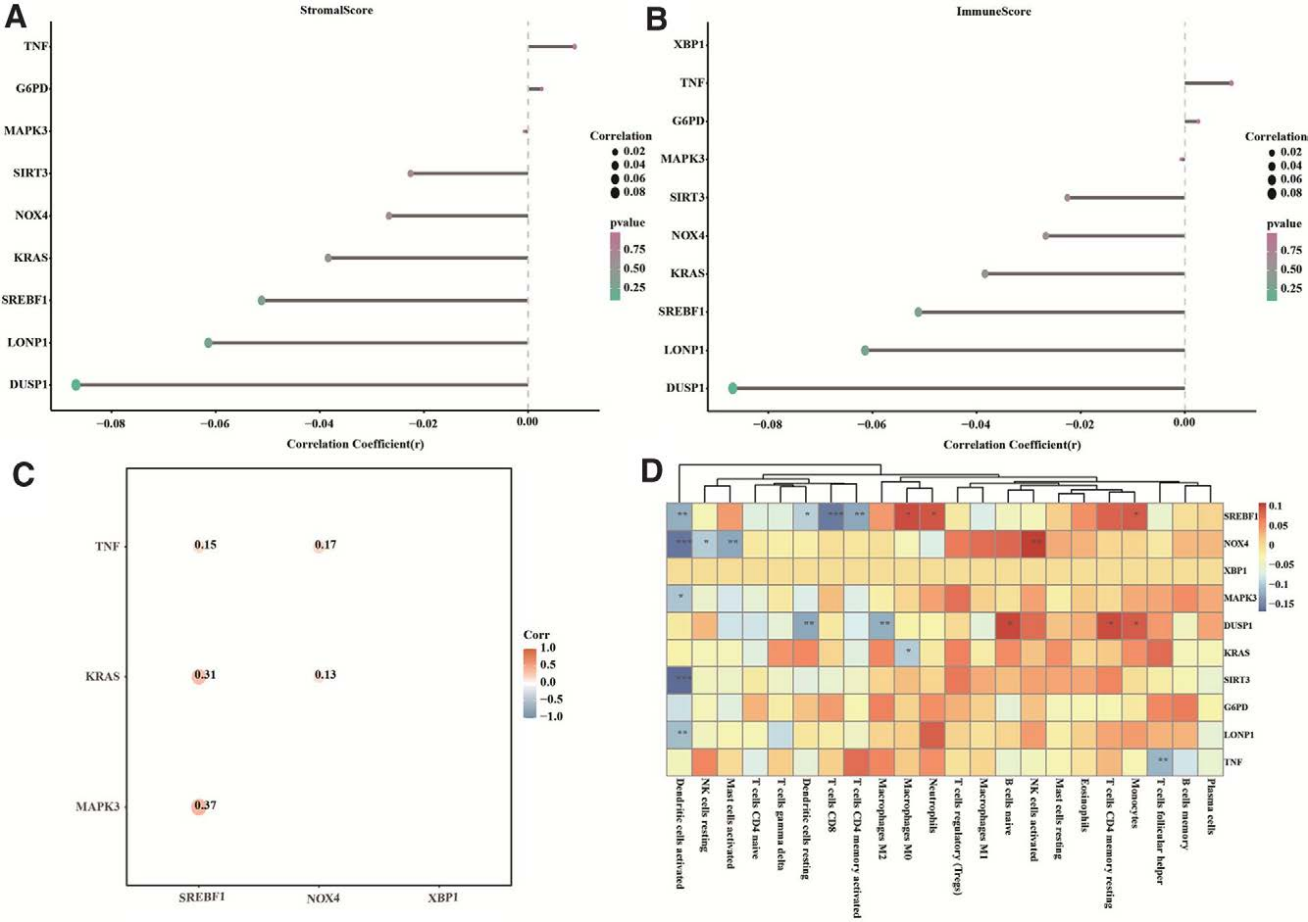
Correlations of hub gene expression with the immune microenvironment of gastric cancer patients from TCGA-STAD. (A, B) Correlations of hub gene expression with stromal scores (A) and immune scores (B). (C) Correlations of hub gene expression with immune-related gene expression. (D) Correlations of hub gene expression with immune cell infiltration. TCGA-STAD = The Cancer Genome Atlas stomach adenocarcinoma dataset.

Considering the relationship between ferroptosis and immune responses in tumor cells,^[38,39]^ we sought to investigate the correlation of the hub genes with the tumor immune microenvironment. As shown in Figure 10A and B, G6PD and TNF expressions positively correlated with both stromal scores and immune scores, whereas NOX4, SREBF1, MAPK3, DUSP1, KRAS, SIRT3, and LONP1 expressions negatively correlated with both stromal scores and immune scores. We also observed significant positive correlations between SREBF1 expression and KRAS/TNF/MAPK3 expression and between NOX4 expression and KRAS/TNF expression (Fig. 10C). Furthermore, the expression of DUSP1, NOX4, SREBF1, TNF, or KRAS was associated with the infiltration of at least one subtype of immune cells (Fig. 10D). Taken together, these data suggest that the ferroptosis-related hub genes are highly related to the immune microenvironment of gastric cancer.

## 4. Discussion

In this study, we identified 21 ferroptosis-related DEGs in therapy-resistant gastric cancer samples compared with therapy-responsive samples from two GEO cohorts. The PPI network revealed 10 hub genes that were mainly enriched in terms associated with oxidative stress and ROS metabolism. A hub gene-based risk model composed of DUSP1, TNF, NOX4, and LONP1 could predict the overall survival of the patients from TCGA-STAD. Moreover, the expressions of the hub genes strongly correlated with the immune microenvironment of gastric tumors in TCGA-STAD. Thus, the hub gene-based risk model is a potential overall survival predictor for gastric cancer. Modulating ferroptosis by targeting the hub genes may overcome therapy resistance in gastric cancer.

Of the 21 ferroptosis-related DEGs in therapy-resistant gastric cancer samples, the PPI network revealed 10 hub genes. GO annotation indicated that the hub genes were predominantly linked to the biological processes related to oxidative stress and ROS. KEGG analysis demonstrated that the hub genes were enriched in the pathways related to immune regulation, lipid metabolism, and cancer progression. It is well-established that the dysregulation of ROS generation, oxidative stress defense ability, and lipid metabolism underlies the mechanism of ferroptosis.^[7]^ Additionally, ferroptosis is immunogenic as evidenced by triggering ferroptosis-dependent immunogenic cell death in preclinical models and promoting the phenotypic maturation of bone marrow-derived dendritic cells.^[38,39]^ Thus, the ferroptosis-related hub genes might contribute to therapy resistance in gastric cancer through dysregulated redox state and immune microenvironment.

Then, we validated the aberrant hub gene expressions in gastric tumor samples from the TCGA cohort. We found that compared with those in normal samples, gastric tumor samples had significantly increased TNF, NOX4, and LONP1 mRNA levels and decreased DUSP1 mRNA levels. Wu et al have shown that TNF signaling promotes cystine uptake and biosynthesis of glutathione to protect fibroblasts from ferroptosis. TNF inhibition may induce ferroptotic cell death, serving as a potential therapeutic strategy against arthritis.^[40]^ On the other hand, TNF promotes the activation of NOXs and ROS generation through the NF-κB pathway,^[41]^ consistent with the upregulation of NOX4 expression seen in TCGA tumor samples. We speculate that the role of TNF in ferroptosis depends on the cell types. MAPK phosphatase DUSP1 plays an important role in regulating the functions of the MAPK family in cancer cells. Shen et al have shown that DUSP1 is significantly downregulated in TCGA-STAD, consistent with our finding.^[14]^ However, Teng et al have demonstrated that DUSP1 is upregulated in apatinib-resistant gastric cell lines and induces apatinib resistance by activating the MAPK pathway.^[42]^ Furthermore, increased DUSP1 inhibits autophagy-dependent ferroptosis in pancreatic cancer.^[43]^ Thus, the role of DUSP1 in ferroptosis and drug resistance in gastric cancer remains elusive and needs further exploration. Luo et al have observed LONP1 upregulation in *Helicobacter pylori*-Induced gastric carcinogenesis.^[15]^ Moreover, LONP1 is upregulated in erastin-induced ferroptosis in human pancreatic ductal adenocarcinoma cells. Inhibition of LONP1 enhances GPX4 expression to protect the cells from ferroptosis.^[44]^ These findings are consistent with ours and indicate active ferroptosis in therapy-resistant gastric cancer.

Next, we analyzed the prognostic value of the hub genes to investigate their clinical significance. Studies have shown that the TNF family of cytokines and their receptors such as tumor necrosis factor receptor-associated factor 2 and TNF-like ligand 1A are associated with the prognosis of gastric cancer.^[45,46]^ In addition, NOX4 predicts a poor prognosis of gastric cancer patients.^[47,48]^ However, the prognostic values of DUSP1 and LONP1 remain unclear. In this study, the results of multivariable Cox regression analysis showed that DUSP1, TNF, NOX4, and LONP1 were independent predictors of the overall survival of the TCGA cohort. Based on these data, we established a predictive risk model containing the four hub genes. Although we did not see significant correlations of the risk scores with the age, gender, tumor grading, and tumor staging in the TCGA dataset, the result of DCA suggested that the risk model performed well in predicting the overall survival of TCGA patients.

Ferroptosis is involved in T cell immunity and cancer immunotherapy.^[38,39]^ Gastric cancer patients with appropriate tumor immune microenvironment may benefit from immunotherapy when given alone or in combination with other therapy.^[49]^ Ma et al have identified two distinct ferroptosis subtypes in gastric cancer patients with significantly different prognosis and immune cell infiltration, suggesting that individualized immunotherapy according to ferroptosis levels of individual tumors may improve the outcomes of patients with gastric cancer.^[50]^ In this study, we observed correlations of the hub genes with stromal/immune scores, immune-related gene expression, as well as immune cell infiltration, suggesting that ferroptosis dysregulation may contribute to immunotherapy resistance in gastric cancer by modulating the immune microenvironment of the tumor.

In our study, we specifically investigated ferroptosis-related genes associated with therapy resistance in gastric cancer. On the other hand, Chen et al focused on understanding trastuzumab resistance in gastric cancer, identifying GNGT1, KRT7, KRT16, SOX9, and TIMP1 as promising diagnostic biomarkers for gastric cancer. Notably, upregulation of KRT16 was correlated with overall survival in gastric cancer patients.^[51]^ Comparing the two studies, both identified hub genes associated with therapy resistance in gastric cancer, albeit focusing on different therapeutic strategies. While there is no direct overlap in the identified hub genes, both studies underscore the complexity of therapy resistance mechanisms in gastric cancer and provide valuable insights into potential diagnostic and therapeutic biomarkers. Integrating findings from different studies could provide a more comprehensive understanding of therapy resistance mechanisms in gastric cancer and facilitate the development of effective treatment strategies.

Nevertheless, this study has several limitations. First, there is a lack of the therapy data of the GSE cohorts. Therapy resistance may develop through different mechanisms based on the treatment options. Second, the predictive performance of the hub gene-based risk model needs to be further validated in an independent testing cohort.

In summary, we identified ferroptosis-related hub genes involved in therapy resistance in gastric cancer and constructed a hub gene-based risk signature for predicting the overall survival of patients. This study provides several ferroptosis-related genes as potential therapeutic targets for reversing the therapy resistance in combating gastric cancer.

## Author contributions

**Conceptualization:** Jieli Yu, Huoguo Chen.

**Data curation:** Jieli Yu, Hua Li, Can Huang.

**Formal analysis:** Jieli Yu, Hua Li, Can Huang.

**Funding acquisition:** Hua Li.

**Investigation:** Jieli Yu, Can Huang.

**Methodology:** Jieli Yu, Huoguo Chen.

**Project administration:** Jieli Yu, Hua Li, Can Huang.

**Resources:** Jieli Yu, Hua Li.

**Software:** Jieli Yu, Can Huang.

**Supervision:** Jieli Yu, Hua Li, Can Huang.

**Validation:** Jieli Yu, Can Huang, Huoguo Chen.

**Visualization:** Jieli Yu.

**Writing – original draft:** Jieli Yu, Can Huang.

**Writing – review & editing:** Jieli Yu, Hua Li, Huoguo Chen.

## Supplementary Material
