# Supplementary figures and images for: Identification and characterization of ferroptosis-related genes in therapy-resistant gastric cancer
Source: Medicine (Baltimore). 2024 May 17;103(20):e38193. doi: 10.1097/MD.0000000000038193 (PMC11098190; doi:10.1097/MD.0000000000038193)

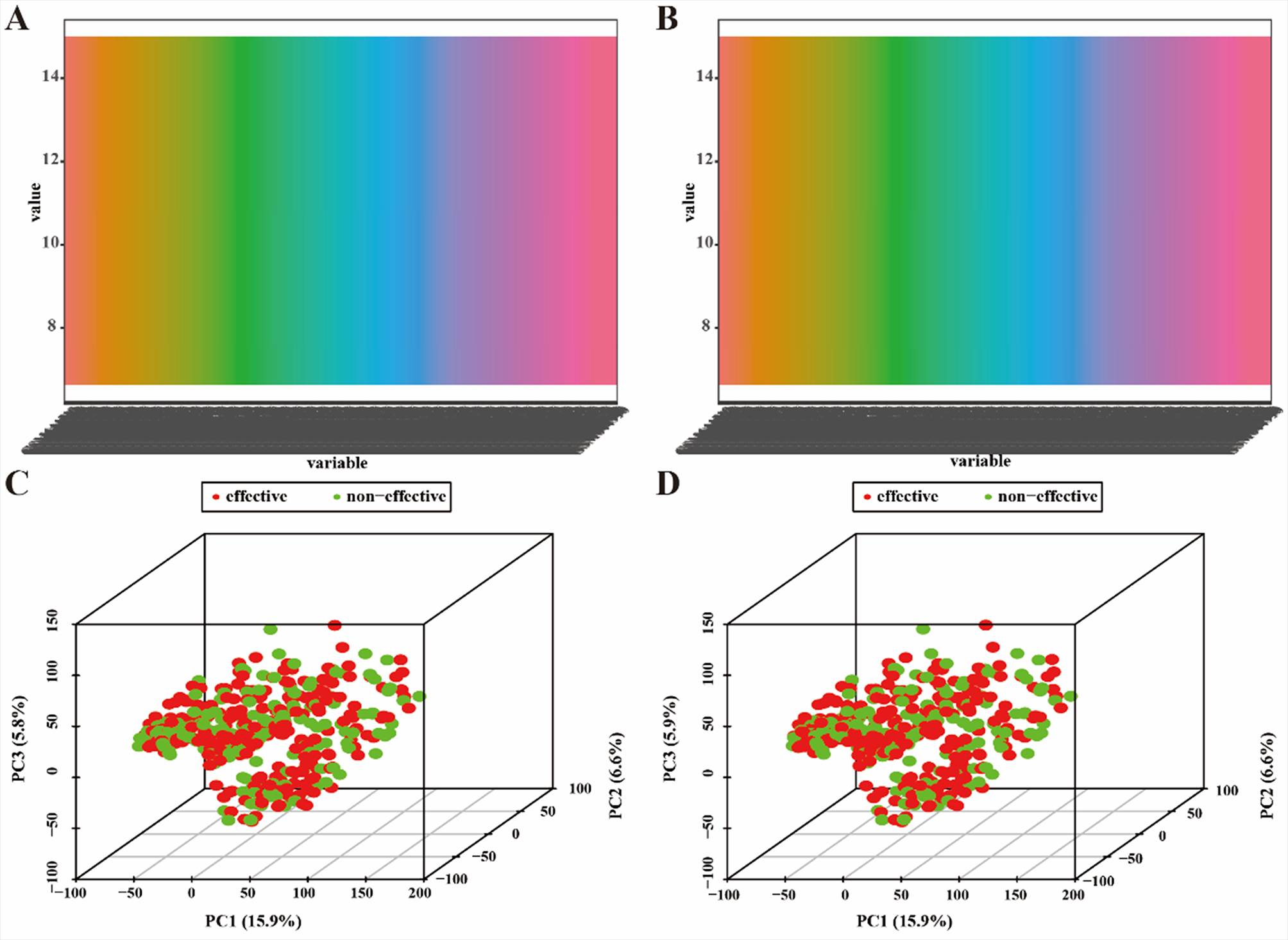

Supplement: Supplementary file 1 [file medi-103-e38193-s001.jpg]
