# Supplementary material for: Identification and characterization of ferroptosis-related genes in therapy-resistant gastric cancer
Source: Medicine (Baltimore). 2024 May 17;103(20):e38193. doi: 10.1097/MD.0000000000038193 (PMC11098190; doi:10.1097/MD.0000000000038193)
Supplement: Supplementary file 2 [file medi-103-e38193-s002.docx]

**Table S1** Gene Ontology analysis

| ONTOLOGY | ID | Description | GeneRatio | BgRatio | pvalue | p.adjust | qvalue | geneID | Count |
| --- | --- | --- | --- | --- | --- | --- | --- | --- | --- |
| BP | GO:0006979 | response to oxidative stress | 7月10日 | 458/18866 | 5.35E-10 | 6.85E-07 | 2.52E-07 | TNF,MAPK3,SIRT3,NOX4,G6PD,DUSP1,LONP1 | 7 |
| BP | GO:0034599 | cellular response to oxidative stress | 6月10日 | 310/18866 | 3.73E-09 | 1.89E-06 | 6.94E-07 | TNF,MAPK3,SIRT3,NOX4,G6PD,LONP1 | 6 |
| BP | GO:0007568 | aging | 6月10日 | 319/18866 | 4.42E-09 | 1.89E-06 | 6.94E-07 | MAPK3,SREBF1,SIRT3,NOX4,KRAS,LONP1 | 6 |
| BP | GO:0062197 | cellular response to chemical stress | 6月10日 | 360/18866 | 9.12E-09 | 2.92E-06 | 1.07E-06 | TNF,MAPK3,SIRT3,NOX4,G6PD,LONP1 | 6 |
| BP | GO:0070372 | regulation of ERK1 and ERK2 cascade | 5月10日 | 306/18866 | 2.56E-07 | 6.56E-05 | 2.41E-05 | TNF,MAPK3,SIRT3,NOX4,DUSP1 | 5 |
| BP | GO:0070371 | ERK1 and ERK2 cascade | 5月10日 | 325/18866 | 3.45E-07 | 7.37E-05 | 2.71E-05 | TNF,MAPK3,SIRT3,NOX4,DUSP1 | 5 |
| BP | GO:0043405 | regulation of MAP kinase activity | 5月10日 | 342/18866 | 4.45E-07 | 8.13E-05 | 2.99E-05 | TNF,MAPK3,NOX4,KRAS,DUSP1 | 5 |
| BP | GO:0030324 | lung development | 4月10日 | 177/18866 | 1.50E-06 | 0.000234 | 8.60E-05 | TNF,MAPK3,SREBF1,KRAS | 4 |
| BP | GO:0030323 | respiratory tube development | 4月10日 | 181/18866 | 1.65E-06 | 0.000234 | 8.60E-05 | TNF,MAPK3,SREBF1,KRAS | 4 |
| BP | GO:0071675 | regulation of mononuclear cell migration | 3月10日 | 51/18866 | 2.20E-06 | 0.000254 | 9.32E-05 | TNF,MAPK3,DUSP1 | 3 |
| BP | GO:0060425 | lung morphogenesis | 3月10日 | 52/18866 | 2.34E-06 | 0.000254 | 9.32E-05 | TNF,MAPK3,KRAS | 3 |
| BP | GO:2000377 | regulation of reactive oxygen species metabolic process | 4月10日 | 200/18866 | 2.45E-06 | 0.000254 | 9.32E-05 | TNF,SIRT3,NOX4,G6PD | 4 |
| BP | GO:0060541 | respiratory system development | 4月10日 | 204/18866 | 2.65E-06 | 0.000254 | 9.32E-05 | TNF,MAPK3,SREBF1,KRAS | 4 |
| BP | GO:0051353 | positive regulation of oxidoreductase activity | 3月10日 | 55/18866 | 2.77E-06 | 0.000254 | 9.32E-05 | TNF,SIRT3,KRAS | 3 |
| BP | GO:0000302 | response to reactive oxygen species | 4月10日 | 235/18866 | 4.65E-06 | 0.000397 | 0.000146 | TNF,MAPK3,SIRT3,DUSP1 | 4 |
| BP | GO:0006801 | superoxide metabolic process | 3月10日 | 74/18866 | 6.82E-06 | 0.000545 | 0.0002 | TNF,SIRT3,NOX4 | 3 |
| BP | GO:0043406 | positive regulation of MAP kinase activity | 4月10日 | 264/18866 | 7.37E-06 | 0.000555 | 0.000204 | TNF,MAPK3,NOX4,KRAS | 4 |
| BP | GO:0048708 | astrocyte differentiation | 3月10日 | 83/18866 | 9.64E-06 | 0.000685 | 0.000252 | TNF,MAPK3,KRAS | 3 |
| BP | GO:0072593 | reactive oxygen species metabolic process | 4月10日 | 288/18866 | 1.04E-05 | 0.0007 | 0.000257 | TNF,SIRT3,NOX4,G6PD | 4 |
| BP | GO:0051146 | striated muscle cell differentiation | 4月10日 | 295/18866 | 1.14E-05 | 0.000701 | 0.000258 | NOX4,G6PD,KRAS,XBP1 | 4 |
| BP | GO:0046889 | positive regulation of lipid biosynthetic process | 3月10日 | 88/18866 | 1.15E-05 | 0.000701 | 0.000258 | TNF,SREBF1,SIRT3 | 3 |
| BP | GO:1901033 | positive regulation of response to reactive oxygen species | 2月10日 | 11/18866 | 1.39E-05 | 0.00078 | 0.000287 | TNF,SIRT3 | 2 |
| BP | GO:0051591 | response to cAMP | 3月10日 | 94/18866 | 1.40E-05 | 0.00078 | 0.000287 | SREBF1,NOX4,DUSP1 | 3 |
| BP | GO:0071674 | mononuclear cell migration | 3月10日 | 97/18866 | 1.54E-05 | 0.000819 | 0.000301 | TNF,MAPK3,DUSP1 | 3 |
| BP | GO:0090154 | positive regulation of sphingolipid biosynthetic process | 2月10日 | 12/18866 | 1.66E-05 | 0.000819 | 0.000301 | TNF,SIRT3 | 2 |
| BP | GO:2000304 | positive regulation of ceramide biosynthetic process | 2月10日 | 12/18866 | 1.66E-05 | 0.000819 | 0.000301 | TNF,SIRT3 | 2 |
| BP | GO:0061043 | regulation of vascular wound healing | 2月10日 | 13/18866 | 1.97E-05 | 0.000874 | 0.000321 | TNF,XBP1 | 2 |
| BP | GO:2000121 | regulation of removal of superoxide radicals | 2月10日 | 13/18866 | 1.97E-05 | 0.000874 | 0.000321 | TNF,SIRT3 | 2 |
| BP | GO:2000379 | positive regulation of reactive oxygen species metabolic process | 3月10日 | 106/18866 | 2.01E-05 | 0.000874 | 0.000321 | TNF,SIRT3,NOX4 | 3 |
| BP | GO:0071902 | positive regulation of protein serine/threonine kinase activity | 4月10日 | 345/18866 | 2.12E-05 | 0.000874 | 0.000321 | TNF,MAPK3,NOX4,KRAS | 4 |
| BP | GO:0048545 | response to steroid hormone | 4月10日 | 346/18866 | 2.14E-05 | 0.000874 | 0.000321 | TNF,SREBF1,KRAS,DUSP1 | 4 |
| BP | GO:2000278 | regulation of DNA biosynthetic process | 3月10日 | 109/18866 | 2.19E-05 | 0.000874 | 0.000321 | MAPK3,NOX4,DUSP1 | 3 |
| BP | GO:0050708 | regulation of protein secretion | 4月10日 | 352/18866 | 2.29E-05 | 0.000888 | 0.000326 | TNF,SREBF1,SIRT3,XBP1 | 4 |
| BP | GO:0010038 | response to metal ion | 4月10日 | 366/18866 | 2.67E-05 | 0.000988 | 0.000363 | MAPK3,G6PD,DUSP1,LONP1 | 4 |
| BP | GO:0051341 | regulation of oxidoreductase activity | 3月10日 | 117/18866 | 2.70E-05 | 0.000988 | 0.000363 | TNF,SIRT3,KRAS | 3 |
| BP | GO:0002791 | regulation of peptide secretion | 4月10日 | 381/18866 | 3.12E-05 | 0.00111 | 0.000408 | TNF,SREBF1,SIRT3,XBP1 | 4 |
| BP | GO:0042692 | muscle cell differentiation | 4月10日 | 390/18866 | 3.42E-05 | 0.001184 | 0.000435 | NOX4,G6PD,KRAS,XBP1 | 4 |
| BP | GO:0051023 | regulation of immunoglobulin secretion | 2月10日 | 18/18866 | 3.85E-05 | 0.001264 | 0.000465 | TNF,XBP1 | 2 |
| BP | GO:2000303 | regulation of ceramide biosynthetic process | 2月10日 | 18/18866 | 3.85E-05 | 0.001264 | 0.000465 | TNF,SIRT3 | 2 |
| BP | GO:0046683 | response to organophosphorus | 3月10日 | 134/18866 | 4.05E-05 | 0.001297 | 0.000477 | SREBF1,NOX4,DUSP1 | 3 |
| BP | GO:0090153 | regulation of sphingolipid biosynthetic process | 2月10日 | 19/18866 | 4.30E-05 | 0.001312 | 0.000482 | TNF,SIRT3 | 2 |
| BP | GO:1905038 | regulation of membrane lipid metabolic process | 2月10日 | 19/18866 | 4.30E-05 | 0.001312 | 0.000482 | TNF,SIRT3 | 2 |
| BP | GO:1900409 | positive regulation of cellular response to oxidative stress | 2月10日 | 20/18866 | 4.78E-05 | 0.001423 | 0.000523 | TNF,SIRT3 | 2 |
| BP | GO:0010893 | positive regulation of steroid biosynthetic process | 2月10日 | 21/18866 | 5.28E-05 | 0.001455 | 0.000535 | TNF,SREBF1 | 2 |
| BP | GO:0048305 | immunoglobulin secretion | 2月10日 | 21/18866 | 5.28E-05 | 0.001455 | 0.000535 | TNF,XBP1 | 2 |
| BP | GO:0061042 | vascular wound healing | 2月10日 | 21/18866 | 5.28E-05 | 0.001455 | 0.000535 | TNF,XBP1 | 2 |
| BP | GO:0051384 | response to glucocorticoid | 3月10日 | 147/18866 | 5.34E-05 | 0.001455 | 0.000535 | TNF,KRAS,DUSP1 | 3 |
| BP | GO:0014074 | response to purine-containing compound | 3月10日 | 149/18866 | 5.56E-05 | 0.001458 | 0.000536 | SREBF1,NOX4,DUSP1 | 3 |
| BP | GO:0048732 | gland development | 4月10日 | 443/18866 | 5.63E-05 | 0.001458 | 0.000536 | TNF,MAPK3,KRAS,XBP1 | 4 |
| BP | GO:0051000 | positive regulation of nitric-oxide synthase activity | 2月10日 | 22/18866 | 5.81E-05 | 0.001458 | 0.000536 | TNF,KRAS | 2 |
| BP | GO:1902884 | positive regulation of response to oxidative stress | 2月10日 | 22/18866 | 5.81E-05 | 0.001458 | 0.000536 | TNF,SIRT3 | 2 |
| BP | GO:0045834 | positive regulation of lipid metabolic process | 3月10日 | 153/18866 | 6.02E-05 | 0.001482 | 0.000545 | TNF,SREBF1,SIRT3 | 3 |
| BP | GO:0035051 | cardiocyte differentiation | 3月10日 | 158/18866 | 6.62E-05 | 0.001556 | 0.000572 | MAPK3,NOX4,G6PD | 3 |
| BP | GO:0009306 | protein secretion | 4月10日 | 462/18866 | 6.63E-05 | 0.001556 | 0.000572 | TNF,SREBF1,SIRT3,XBP1 | 4 |
| BP | GO:0035592 | establishment of protein localization to extracellular region | 4月10日 | 463/18866 | 6.69E-05 | 0.001556 | 0.000572 | TNF,SREBF1,SIRT3,XBP1 | 4 |
| BP | GO:0071692 | protein localization to extracellular region | 4月10日 | 470/18866 | 7.09E-05 | 0.001579 | 0.00058 | TNF,SREBF1,SIRT3,XBP1 | 4 |
| BP | GO:0031667 | response to nutrient levels | 4月10日 | 473/18866 | 7.27E-05 | 0.001579 | 0.00058 | MAPK3,SREBF1,G6PD,XBP1 | 4 |
| BP | GO:0009267 | cellular response to starvation | 3月10日 | 163/18866 | 7.27E-05 | 0.001579 | 0.00058 | MAPK3,SREBF1,XBP1 | 3 |
| BP | GO:0031960 | response to corticosteroid | 3月10日 | 164/18866 | 7.40E-05 | 0.001579 | 0.00058 | TNF,KRAS,DUSP1 | 3 |
| BP | GO:0046165 | alcohol biosynthetic process | 3月10日 | 164/18866 | 7.40E-05 | 0.001579 | 0.00058 | TNF,SREBF1,G6PD | 3 |
| BP | GO:0034250 | positive regulation of cellular amide metabolic process | 3月10日 | 167/18866 | 7.81E-05 | 0.001639 | 0.000602 | TNF,MAPK3,SIRT3 | 3 |
| BP | GO:0019430 | removal of superoxide radicals | 2月10日 | 26/18866 | 8.16E-05 | 0.001673 | 0.000615 | TNF,SIRT3 | 2 |
| BP | GO:0034614 | cellular response to reactive oxygen species | 3月10日 | 170/18866 | 8.23E-05 | 0.001673 | 0.000615 | TNF,MAPK3,SIRT3 | 3 |
| BP | GO:0050714 | positive regulation of protein secretion | 3月10日 | 172/18866 | 8.53E-05 | 0.001705 | 0.000627 | TNF,SIRT3,XBP1 | 3 |
| BP | GO:0071450 | cellular response to oxygen radical | 2月10日 | 28/18866 | 9.49E-05 | 0.001813 | 0.000666 | TNF,SIRT3 | 2 |
| BP | GO:0071451 | cellular response to superoxide | 2月10日 | 28/18866 | 9.49E-05 | 0.001813 | 0.000666 | TNF,SIRT3 | 2 |
| BP | GO:1902932 | positive regulation of alcohol biosynthetic process | 2月10日 | 28/18866 | 9.49E-05 | 0.001813 | 0.000666 | TNF,SREBF1 | 2 |
| BP | GO:0050796 | regulation of insulin secretion | 3月10日 | 181/18866 | 9.92E-05 | 0.001867 | 0.000686 | TNF,SREBF1,SIRT3 | 3 |
| BP | GO:0060441 | epithelial tube branching involved in lung morphogenesis | 2月10日 | 29/18866 | 0.000102 | 0.00189 | 0.000695 | TNF,KRAS | 2 |
| BP | GO:0000303 | response to superoxide | 2月10日 | 30/18866 | 0.000109 | 0.001967 | 0.000723 | TNF,SIRT3 | 2 |
| BP | GO:0060055 | angiogenesis involved in wound healing | 2月10日 | 30/18866 | 0.000109 | 0.001967 | 0.000723 | TNF,XBP1 | 2 |
| BP | GO:0000305 | response to oxygen radical | 2月10日 | 31/18866 | 0.000117 | 0.002017 | 0.000742 | TNF,SIRT3 | 2 |
| BP | GO:0032770 | positive regulation of monooxygenase activity | 2月10日 | 31/18866 | 0.000117 | 0.002017 | 0.000742 | TNF,KRAS | 2 |
| BP | GO:0045940 | positive regulation of steroid metabolic process | 2月10日 | 31/18866 | 0.000117 | 0.002017 | 0.000742 | TNF,SREBF1 | 2 |
| BP | GO:0002793 | positive regulation of peptide secretion | 3月10日 | 193/18866 | 0.00012 | 0.002048 | 0.000753 | TNF,SIRT3,XBP1 | 3 |
| BP | GO:0071897 | DNA biosynthetic process | 3月10日 | 196/18866 | 0.000126 | 0.002092 | 0.000769 | MAPK3,NOX4,DUSP1 | 3 |
| BP | GO:0006694 | steroid biosynthetic process | 3月10日 | 197/18866 | 0.000127 | 0.002092 | 0.000769 | TNF,SREBF1,G6PD | 3 |
| BP | GO:0009749 | response to glucose | 3月10日 | 197/18866 | 0.000127 | 0.002092 | 0.000769 | SREBF1,NOX4,XBP1 | 3 |
| BP | GO:0009746 | response to hexose | 3月10日 | 202/18866 | 0.000137 | 0.002225 | 0.000818 | SREBF1,NOX4,XBP1 | 3 |
| BP | GO:0042594 | response to starvation | 3月10日 | 206/18866 | 0.000146 | 0.002304 | 0.000847 | MAPK3,SREBF1,XBP1 | 3 |
| BP | GO:0034284 | response to monosaccharide | 3月10日 | 207/18866 | 0.000148 | 0.002304 | 0.000847 | SREBF1,NOX4,XBP1 | 3 |
| BP | GO:0046890 | regulation of lipid biosynthetic process | 3月10日 | 207/18866 | 0.000148 | 0.002304 | 0.000847 | TNF,SREBF1,SIRT3 | 3 |
| BP | GO:0071222 | cellular response to lipopolysaccharide | 3月10日 | 208/18866 | 0.00015 | 0.002309 | 0.000849 | TNF,MAPK3,XBP1 | 3 |
| BP | GO:0002685 | regulation of leukocyte migration | 3月10日 | 212/18866 | 0.000158 | 0.00239 | 0.000879 | TNF,MAPK3,DUSP1 | 3 |
| BP | GO:0030073 | insulin secretion | 3月10日 | 213/18866 | 0.000161 | 0.00239 | 0.000879 | TNF,SREBF1,SIRT3 | 3 |
| BP | GO:0090276 | regulation of peptide hormone secretion | 3月10日 | 213/18866 | 0.000161 | 0.00239 | 0.000879 | TNF,SREBF1,SIRT3 | 3 |
| BP | GO:0043523 | regulation of neuron apoptotic process | 3月10日 | 214/18866 | 0.000163 | 0.002396 | 0.000881 | TNF,G6PD,KRAS | 3 |
| BP | GO:0070374 | positive regulation of ERK1 and ERK2 cascade | 3月10日 | 215/18866 | 0.000165 | 0.002398 | 0.000882 | TNF,MAPK3,NOX4 | 3 |
| BP | GO:0090322 | regulation of superoxide metabolic process | 2月10日 | 37/18866 | 0.000167 | 0.002398 | 0.000882 | TNF,SIRT3 | 2 |
| BP | GO:0032094 | response to food | 2月10日 | 38/18866 | 0.000176 | 0.002503 | 0.00092 | SREBF1,G6PD | 2 |
| BP | GO:0031669 | cellular response to nutrient levels | 3月10日 | 221/18866 | 0.000179 | 0.002519 | 0.000926 | MAPK3,SREBF1,XBP1 | 3 |
| BP | GO:0071219 | cellular response to molecule of bacterial origin | 3月10日 | 222/18866 | 0.000181 | 0.002525 | 0.000928 | TNF,MAPK3,XBP1 | 3 |
| BP | GO:0007431 | salivary gland development | 2月10日 | 39/18866 | 0.000185 | 0.002552 | 0.000938 | TNF,XBP1 | 2 |
| BP | GO:1902895 | positive regulation of pri-miRNA transcription by RNA polymerase II | 2月10日 | 40/18866 | 0.000195 | 0.002657 | 0.000977 | TNF,SREBF1 | 2 |
| BP | GO:0010001 | glial cell differentiation | 3月10日 | 230/18866 | 0.000201 | 0.002714 | 0.000998 | TNF,MAPK3,KRAS | 3 |
| BP | GO:0009743 | response to carbohydrate | 3月10日 | 233/18866 | 0.000209 | 0.002791 | 0.001026 | SREBF1,NOX4,XBP1 | 3 |
| BP | GO:0045444 | fat cell differentiation | 3月10日 | 235/18866 | 0.000215 | 0.002832 | 0.001041 | TNF,SREBF1,XBP1 | 3 |
| BP | GO:0032872 | regulation of stress-activated MAPK cascade | 3月10日 | 237/18866 | 0.00022 | 0.002874 | 0.001057 | TNF,MAPK3,DUSP1 | 3 |
| BP | GO:0051154 | negative regulation of striated muscle cell differentiation | 2月10日 | 43/18866 | 0.000226 | 0.002889 | 0.001062 | G6PD,XBP1 | 2 |
| BP | GO:1901031 | regulation of response to reactive oxygen species | 2月10日 | 43/18866 | 0.000226 | 0.002889 | 0.001062 | TNF,SIRT3 | 2 |
| BP | GO:0070302 | regulation of stress-activated protein kinase signaling cascade | 3月10日 | 240/18866 | 0.000228 | 0.002894 | 0.001064 | TNF,MAPK3,DUSP1 | 3 |
| BP | GO:0051402 | neuron apoptotic process | 3月10日 | 245/18866 | 0.000243 | 0.003044 | 0.001119 | TNF,G6PD,KRAS | 3 |
| BP | GO:0071216 | cellular response to biotic stimulus | 3月10日 | 246/18866 | 0.000246 | 0.003044 | 0.001119 | TNF,MAPK3,XBP1 | 3 |
| BP | GO:0014002 | astrocyte development | 2月10日 | 45/18866 | 0.000247 | 0.003044 | 0.001119 | TNF,KRAS | 2 |
| BP | GO:0009636 | response to toxic substance | 3月10日 | 250/18866 | 0.000258 | 0.00314 | 0.001154 | TNF,MAPK3,SIRT3 | 3 |
| BP | GO:0031668 | cellular response to extracellular stimulus | 3月10日 | 253/18866 | 0.000267 | 0.003221 | 0.001184 | MAPK3,SREBF1,XBP1 | 3 |
| BP | GO:0030072 | peptide hormone secretion | 3月10日 | 257/18866 | 0.000279 | 0.003342 | 0.001228 | TNF,SREBF1,SIRT3 | 3 |
| BP | GO:1901617 | organic hydroxy compound biosynthetic process | 3月10日 | 258/18866 | 0.000283 | 0.003349 | 0.001231 | TNF,SREBF1,G6PD | 3 |
| BP | GO:0046883 | regulation of hormone secretion | 3月10日 | 267/18866 | 0.000312 | 0.003634 | 0.001336 | TNF,SREBF1,SIRT3 | 3 |
| BP | GO:0006984 | ER-nucleus signaling pathway | 2月10日 | 51/18866 | 0.000318 | 0.003634 | 0.001336 | SREBF1,XBP1 | 2 |
| BP | GO:0035272 | exocrine system development | 2月10日 | 51/18866 | 0.000318 | 0.003634 | 0.001336 | TNF,XBP1 | 2 |
| BP | GO:1902893 | regulation of pri-miRNA transcription by RNA polymerase II | 2月10日 | 51/18866 | 0.000318 | 0.003634 | 0.001336 | TNF,SREBF1 | 2 |
| BP | GO:0050999 | regulation of nitric-oxide synthase activity | 2月10日 | 52/18866 | 0.000331 | 0.003745 | 0.001377 | TNF,KRAS | 2 |
| BP | GO:0061614 | pri-miRNA transcription by RNA polymerase II | 2月10日 | 53/18866 | 0.000343 | 0.003857 | 0.001418 | TNF,SREBF1 | 2 |
| BP | GO:0051403 | stress-activated MAPK cascade | 3月10日 | 286/18866 | 0.000382 | 0.004255 | 0.001564 | TNF,MAPK3,DUSP1 | 3 |
| BP | GO:0031647 | regulation of protein stability | 3月10日 | 296/18866 | 0.000423 | 0.004658 | 0.001712 | SREBF1,KRAS,XBP1 | 3 |
| BP | GO:0031663 | lipopolysaccharide-mediated signaling pathway | 2月10日 | 59/18866 | 0.000426 | 0.004658 | 0.001712 | TNF,MAPK3 | 2 |
| BP | GO:0031098 | stress-activated protein kinase signaling cascade | 3月10日 | 300/18866 | 0.00044 | 0.00477 | 0.001753 | TNF,MAPK3,DUSP1 | 3 |
| BP | GO:0042063 | gliogenesis | 3月10日 | 307/18866 | 0.00047 | 0.00506 | 0.00186 | TNF,MAPK3,KRAS | 3 |
| BP | GO:1903532 | positive regulation of secretion by cell | 3月10日 | 313/18866 | 0.000498 | 0.00531 | 0.001952 | TNF,SIRT3,XBP1 | 3 |
| BP | GO:0046879 | hormone secretion | 3月10日 | 314/18866 | 0.000502 | 0.005315 | 0.001954 | TNF,SREBF1,SIRT3 | 3 |
| BP | GO:0032768 | regulation of monooxygenase activity | 2月10日 | 66/18866 | 0.000533 | 0.005532 | 0.002033 | TNF,KRAS | 2 |
| BP | GO:2000378 | negative regulation of reactive oxygen species metabolic process | 2月10日 | 66/18866 | 0.000533 | 0.005532 | 0.002033 | SIRT3,G6PD | 2 |
| BP | GO:1901214 | regulation of neuron death | 3月10日 | 321/18866 | 0.000536 | 0.005532 | 0.002033 | TNF,G6PD,KRAS | 3 |
| BP | GO:0009914 | hormone transport | 3月10日 | 323/18866 | 0.000546 | 0.005576 | 0.00205 | TNF,SREBF1,SIRT3 | 3 |
| BP | GO:0046513 | ceramide biosynthetic process | 2月10日 | 67/18866 | 0.000549 | 0.005576 | 0.00205 | TNF,SIRT3 | 2 |
| BP | GO:0071496 | cellular response to external stimulus | 3月10日 | 326/18866 | 0.000561 | 0.00561 | 0.002062 | MAPK3,SREBF1,XBP1 | 3 |
| BP | GO:0002637 | regulation of immunoglobulin production | 2月10日 | 68/18866 | 0.000565 | 0.00561 | 0.002062 | TNF,XBP1 | 2 |
| BP | GO:0051148 | negative regulation of muscle cell differentiation | 2月10日 | 68/18866 | 0.000565 | 0.00561 | 0.002062 | G6PD,XBP1 | 2 |
| BP | GO:0071230 | cellular response to amino acid stimulus | 2月10日 | 69/18866 | 0.000582 | 0.005688 | 0.002091 | TNF,XBP1 | 2 |
| BP | GO:2000573 | positive regulation of DNA biosynthetic process | 2月10日 | 69/18866 | 0.000582 | 0.005688 | 0.002091 | MAPK3,NOX4 | 2 |
| BP | GO:0008202 | steroid metabolic process | 3月10日 | 332/18866 | 0.000591 | 0.005733 | 0.002108 | TNF,SREBF1,G6PD | 3 |
| BP | GO:0032496 | response to lipopolysaccharide | 3月10日 | 334/18866 | 0.000602 | 0.005791 | 0.002129 | TNF,MAPK3,XBP1 | 3 |
| BP | GO:0051047 | positive regulation of secretion | 3月10日 | 340/18866 | 0.000634 | 0.006054 | 0.002225 | TNF,SIRT3,XBP1 | 3 |
| BP | GO:0010506 | regulation of autophagy | 3月10日 | 347/18866 | 0.000672 | 0.006376 | 0.002344 | MAPK3,SREBF1,XBP1 | 3 |
| BP | GO:0006695 | cholesterol biosynthetic process | 2月10日 | 75/18866 | 0.000687 | 0.006422 | 0.002361 | SREBF1,G6PD | 2 |
| BP | GO:1902653 | secondary alcohol biosynthetic process | 2月10日 | 75/18866 | 0.000687 | 0.006422 | 0.002361 | SREBF1,G6PD | 2 |
| BP | GO:0071229 | cellular response to acid chemical | 2月10日 | 76/18866 | 0.000706 | 0.006546 | 0.002406 | TNF,XBP1 | 2 |
| BP | GO:0051222 | positive regulation of protein transport | 3月10日 | 354/18866 | 0.000713 | 0.006563 | 0.002413 | TNF,SIRT3,XBP1 | 3 |
| BP | GO:0002237 | response to molecule of bacterial origin | 3月10日 | 356/18866 | 0.000725 | 0.006577 | 0.002418 | TNF,MAPK3,XBP1 | 3 |
| BP | GO:0033044 | regulation of chromosome organization | 3月10日 | 356/18866 | 0.000725 | 0.006577 | 0.002418 | MAPK3,SREBF1,DUSP1 | 3 |
| BP | GO:0070997 | neuron death | 3月10日 | 360/18866 | 0.000748 | 0.006746 | 0.00248 | TNF,G6PD,KRAS | 3 |
| BP | GO:0016575 | histone deacetylation | 2月10日 | 79/18866 | 0.000762 | 0.006776 | 0.002491 | SREBF1,SIRT3 | 2 |
| BP | GO:0070373 | negative regulation of ERK1 and ERK2 cascade | 2月10日 | 79/18866 | 0.000762 | 0.006776 | 0.002491 | SIRT3,DUSP1 | 2 |
| BP | GO:0051052 | regulation of DNA metabolic process | 3月10日 | 365/18866 | 0.000779 | 0.006877 | 0.002528 | MAPK3,NOX4,DUSP1 | 3 |
| BP | GO:0016126 | sterol biosynthetic process | 2月10日 | 81/18866 | 0.000801 | 0.007024 | 0.002582 | SREBF1,G6PD | 2 |
| BP | GO:1904951 | positive regulation of establishment of protein localization | 3月10日 | 370/18866 | 0.00081 | 0.007057 | 0.002594 | TNF,SIRT3,XBP1 | 3 |
| BP | GO:1902930 | regulation of alcohol biosynthetic process | 2月10日 | 82/18866 | 0.000821 | 0.007101 | 0.00261 | TNF,SREBF1 | 2 |
| BP | GO:0018108 | peptidyl-tyrosine phosphorylation | 3月10日 | 374/18866 | 0.000836 | 0.007183 | 0.00264 | TNF,MAPK3,NOX4 | 3 |
| BP | GO:0018212 | peptidyl-tyrosine modification | 3月10日 | 377/18866 | 0.000856 | 0.007303 | 0.002684 | TNF,MAPK3,NOX4 | 3 |
| BP | GO:0006066 | alcohol metabolic process | 3月10日 | 385/18866 | 0.00091 | 0.007659 | 0.002815 | TNF,SREBF1,G6PD | 3 |
| BP | GO:0031349 | positive regulation of defense response | 3月10日 | 385/18866 | 0.00091 | 0.007659 | 0.002815 | TNF,MAPK3,KRAS | 3 |
| BP | GO:0032755 | positive regulation of interleukin-6 production | 2月10日 | 88/18866 | 0.000945 | 0.007904 | 0.002905 | TNF,XBP1 | 2 |
| BP | GO:0006631 | fatty acid metabolic process | 3月10日 | 396/18866 | 0.000987 | 0.008203 | 0.003015 | MAPK3,SREBF1,XBP1 | 3 |
| BP | GO:0031058 | positive regulation of histone modification | 2月10日 | 92/18866 | 0.001032 | 0.008467 | 0.003113 | MAPK3,SREBF1 | 2 |
| BP | GO:1900407 | regulation of cellular response to oxidative stress | 2月10日 | 92/18866 | 0.001032 | 0.008467 | 0.003113 | TNF,SIRT3 | 2 |
| BP | GO:0006476 | protein deacetylation | 2月10日 | 97/18866 | 0.001146 | 0.009345 | 0.003435 | SREBF1,SIRT3 | 2 |
| BP | GO:0050810 | regulation of steroid biosynthetic process | 2月10日 | 98/18866 | 0.00117 | 0.009476 | 0.003483 | TNF,SREBF1 | 2 |
| BP | GO:0006672 | ceramide metabolic process | 2月10日 | 100/18866 | 0.001218 | 0.009801 | 0.003603 | TNF,SIRT3 | 2 |
| BP | GO:1902882 | regulation of response to oxidative stress | 2月10日 | 101/18866 | 0.001242 | 0.009934 | 0.003652 | TNF,SIRT3 | 2 |
| BP | GO:0019216 | regulation of lipid metabolic process | 3月10日 | 431/18866 | 0.001261 | 0.010023 | 0.003684 | TNF,SREBF1,SIRT3 | 3 |
| BP | GO:0098869 | cellular oxidant detoxification | 2月10日 | 103/18866 | 0.001291 | 0.0102 | 0.00375 | TNF,SIRT3 | 2 |
| BP | GO:1905269 | positive regulation of chromatin organization | 2月10日 | 106/18866 | 0.001366 | 0.01073 | 0.003945 | MAPK3,SREBF1 | 2 |
| BP | GO:0030148 | sphingolipid biosynthetic process | 2月10日 | 107/18866 | 0.001392 | 0.010798 | 0.003969 | TNF,SIRT3 | 2 |
| BP | GO:0035601 | protein deacylation | 2月10日 | 107/18866 | 0.001392 | 0.010798 | 0.003969 | SREBF1,SIRT3 | 2 |
| BP | GO:0009314 | response to radiation | 3月10日 | 447/18866 | 0.0014 | 0.010798 | 0.003969 | NOX4,KRAS,DUSP1 | 3 |
| BP | GO:0032526 | response to retinoic acid | 2月10日 | 108/18866 | 0.001418 | 0.010868 | 0.003995 | SREBF1,DUSP1 | 2 |
| BP | GO:0051090 | regulation of DNA-binding transcription factor activity | 3月10日 | 455/18866 | 0.001474 | 0.011229 | 0.004128 | TNF,MAPK3,KRAS | 3 |
| BP | GO:0098732 | macromolecule deacylation | 2月10日 | 111/18866 | 0.001497 | 0.011338 | 0.004168 | SREBF1,SIRT3 | 2 |
| BP | GO:0043200 | response to amino acid | 2月10日 | 114/18866 | 0.001578 | 0.011812 | 0.004342 | TNF,XBP1 | 2 |
| BP | GO:1990748 | cellular detoxification | 2月10日 | 114/18866 | 0.001578 | 0.011812 | 0.004342 | TNF,SIRT3 | 2 |
| BP | GO:0016570 | histone modification | 3月10日 | 468/18866 | 0.001598 | 0.011894 | 0.004372 | MAPK3,SREBF1,SIRT3 | 3 |
| BP | GO:0060249 | anatomical structure homeostasis | 3月10日 | 469/18866 | 0.001608 | 0.011898 | 0.004374 | MAPK3,NOX4,KRAS | 3 |
| BP | GO:0007569 | cell aging | 2月10日 | 118/18866 | 0.001689 | 0.012427 | 0.004568 | NOX4,KRAS | 2 |
| BP | GO:0051153 | regulation of striated muscle cell differentiation | 2月10日 | 119/18866 | 0.001718 | 0.012563 | 0.004618 | G6PD,XBP1 | 2 |
| BP | GO:0016569 | covalent chromatin modification | 3月10日 | 481/18866 | 0.001729 | 0.012575 | 0.004623 | MAPK3,SREBF1,SIRT3 | 3 |
| BP | GO:0021782 | glial cell development | 2月10日 | 122/18866 | 0.001804 | 0.012974 | 0.004769 | TNF,KRAS | 2 |
| BP | GO:0097237 | cellular response to toxic substance | 2月10日 | 122/18866 | 0.001804 | 0.012974 | 0.004769 | TNF,SIRT3 | 2 |
| BP | GO:0002688 | regulation of leukocyte chemotaxis | 2月10日 | 124/18866 | 0.001863 | 0.013248 | 0.00487 | MAPK3,DUSP1 | 2 |
| BP | GO:0022612 | gland morphogenesis | 2月10日 | 124/18866 | 0.001863 | 0.013248 | 0.00487 | TNF,XBP1 | 2 |
| BP | GO:0055007 | cardiac muscle cell differentiation | 2月10日 | 126/18866 | 0.001923 | 0.013597 | 0.004998 | NOX4,G6PD | 2 |
| BP | GO:0014066 | regulation of phosphatidylinositol 3-kinase signaling | 2月10日 | 127/18866 | 0.001953 | 0.013735 | 0.005049 | TNF,MAPK3 | 2 |
| BP | GO:1903409 | reactive oxygen species biosynthetic process | 2月10日 | 128/18866 | 0.001983 | 0.013873 | 0.0051 | TNF,NOX4 | 2 |
| BP | GO:0019218 | regulation of steroid metabolic process | 2月10日 | 131/18866 | 0.002076 | 0.014442 | 0.005309 | TNF,SREBF1 | 2 |
| BP | GO:0001101 | response to acid chemical | 2月10日 | 132/18866 | 0.002107 | 0.014581 | 0.00536 | TNF,XBP1 | 2 |
| BP | GO:0071333 | cellular response to glucose stimulus | 2月10日 | 134/18866 | 0.002171 | 0.014939 | 0.005491 | NOX4,XBP1 | 2 |
| BP | GO:0045727 | positive regulation of translation | 2月10日 | 136/18866 | 0.002235 | 0.015217 | 0.005594 | TNF,MAPK3 | 2 |
| BP | GO:0071331 | cellular response to hexose stimulus | 2月10日 | 136/18866 | 0.002235 | 0.015217 | 0.005594 | NOX4,XBP1 | 2 |
| BP | GO:0071326 | cellular response to monosaccharide stimulus | 2月10日 | 137/18866 | 0.002268 | 0.015357 | 0.005645 | NOX4,XBP1 | 2 |
| BP | GO:0098754 | detoxification | 2月10日 | 138/18866 | 0.0023 | 0.015496 | 0.005696 | TNF,SIRT3 | 2 |
| BP | GO:0001889 | liver development | 2月10日 | 141/18866 | 0.0024 | 0.016081 | 0.005912 | KRAS,XBP1 | 2 |
| BP | GO:0002687 | positive regulation of leukocyte migration | 2月10日 | 142/18866 | 0.002433 | 0.016222 | 0.005963 | TNF,MAPK3 | 2 |
| BP | GO:0045598 | regulation of fat cell differentiation | 2月10日 | 143/18866 | 0.002467 | 0.016362 | 0.006015 | TNF,XBP1 | 2 |
| BP | GO:0002700 | regulation of production of molecular mediator of immune response | 2月10日 | 144/18866 | 0.002501 | 0.016417 | 0.006035 | TNF,XBP1 | 2 |
| BP | GO:0061008 | hepaticobiliary system development | 2月10日 | 144/18866 | 0.002501 | 0.016417 | 0.006035 | KRAS,XBP1 | 2 |
| BP | GO:0071322 | cellular response to carbohydrate stimulus | 2月10日 | 145/18866 | 0.002535 | 0.016557 | 0.006087 | NOX4,XBP1 | 2 |
| BP | GO:0046467 | membrane lipid biosynthetic process | 2月10日 | 147/18866 | 0.002605 | 0.016838 | 0.006189 | TNF,SIRT3 | 2 |
| BP | GO:0055123 | digestive system development | 2月10日 | 147/18866 | 0.002605 | 0.016838 | 0.006189 | TNF,XBP1 | 2 |
| BP | GO:0031056 | regulation of histone modification | 2月10日 | 149/18866 | 0.002675 | 0.017204 | 0.006324 | MAPK3,SREBF1 | 2 |
| BP | GO:0061041 | regulation of wound healing | 2月10日 | 151/18866 | 0.002746 | 0.017572 | 0.006459 | TNF,XBP1 | 2 |
| BP | GO:0001678 | cellular glucose homeostasis | 2月10日 | 153/18866 | 0.002817 | 0.017853 | 0.006563 | NOX4,XBP1 | 2 |
| BP | GO:0008203 | cholesterol metabolic process | 2月10日 | 153/18866 | 0.002817 | 0.017853 | 0.006563 | SREBF1,G6PD | 2 |
| BP | GO:0014065 | phosphatidylinositol 3-kinase signaling | 2月10日 | 154/18866 | 0.002854 | 0.017906 | 0.006582 | TNF,MAPK3 | 2 |
| BP | GO:0062013 | positive regulation of small molecule metabolic process | 2月10日 | 154/18866 | 0.002854 | 0.017906 | 0.006582 | TNF,SREBF1 | 2 |
| BP | GO:0048754 | branching morphogenesis of an epithelial tube | 2月10日 | 155/18866 | 0.00289 | 0.018046 | 0.006634 | TNF,KRAS | 2 |
| BP | GO:0000187 | activation of MAPK activity | 2月10日 | 156/18866 | 0.002927 | 0.018187 | 0.006685 | TNF,MAPK3 | 2 |
| BP | GO:0032675 | regulation of interleukin-6 production | 2月10日 | 159/18866 | 0.003038 | 0.018608 | 0.00684 | TNF,XBP1 | 2 |
| BP | GO:0051092 | positive regulation of NF-kappaB transcription factor activity | 2月10日 | 159/18866 | 0.003038 | 0.018608 | 0.00684 | TNF,KRAS | 2 |
| BP | GO:1902107 | positive regulation of leukocyte differentiation | 2月10日 | 159/18866 | 0.003038 | 0.018608 | 0.00684 | TNF,XBP1 | 2 |
| BP | GO:1902652 | secondary alcohol metabolic process | 2月10日 | 162/18866 | 0.003152 | 0.019211 | 0.007062 | SREBF1,G6PD | 2 |
| BP | GO:0006665 | sphingolipid metabolic process | 2月10日 | 163/18866 | 0.00319 | 0.019352 | 0.007114 | TNF,SIRT3 | 2 |
| BP | GO:0016125 | sterol metabolic process | 2月10日 | 169/18866 | 0.003424 | 0.020674 | 0.0076 | SREBF1,G6PD | 2 |
| BP | GO:0032635 | interleukin-6 production | 2月10日 | 170/18866 | 0.003464 | 0.020816 | 0.007652 | TNF,XBP1 | 2 |
| BP | GO:0051897 | positive regulation of protein kinase B signaling | 2月10日 | 178/18866 | 0.00379 | 0.02267 | 0.008333 | TNF,NOX4 | 2 |
| BP | GO:0048469 | cell maturation | 2月10日 | 179/18866 | 0.003832 | 0.022813 | 0.008386 | G6PD,XBP1 | 2 |
| BP | GO:2001252 | positive regulation of chromosome organization | 2月10日 | 182/18866 | 0.003958 | 0.023457 | 0.008623 | MAPK3,SREBF1 | 2 |
| BP | GO:1903034 | regulation of response to wounding | 2月10日 | 183/18866 | 0.004001 | 0.0236 | 0.008675 | TNF,XBP1 | 2 |
| BP | GO:0051147 | regulation of muscle cell differentiation | 2月10日 | 186/18866 | 0.00413 | 0.02425 | 0.008914 | G6PD,XBP1 | 2 |
| BP | GO:0043409 | negative regulation of MAPK cascade | 2月10日 | 187/18866 | 0.004174 | 0.024282 | 0.008926 | SIRT3,DUSP1 | 2 |
| BP | GO:0061138 | morphogenesis of a branching epithelium | 2月10日 | 187/18866 | 0.004174 | 0.024282 | 0.008926 | TNF,KRAS | 2 |
| BP | GO:0048015 | phosphatidylinositol-mediated signaling | 2月10日 | 192/18866 | 0.004394 | 0.02545 | 0.009355 | TNF,MAPK3 | 2 |
| BP | GO:0050731 | positive regulation of peptidyl-tyrosine phosphorylation | 2月10日 | 195/18866 | 0.004529 | 0.025996 | 0.009556 | TNF,NOX4 | 2 |
| BP | GO:1902275 | regulation of chromatin organization | 2月10日 | 195/18866 | 0.004529 | 0.025996 | 0.009556 | MAPK3,SREBF1 | 2 |
| BP | GO:0007565 | female pregnancy | 2月10日 | 196/18866 | 0.004574 | 0.026023 | 0.009566 | MAPK3,KRAS | 2 |
| BP | GO:0048017 | inositol lipid-mediated signaling | 2月10日 | 196/18866 | 0.004574 | 0.026023 | 0.009566 | TNF,MAPK3 | 2 |
| BP | GO:0001935 | endothelial cell proliferation | 2月10日 | 199/18866 | 0.004712 | 0.026687 | 0.00981 | TNF,XBP1 | 2 |
| BP | GO:0051054 | positive regulation of DNA metabolic process | 2月10日 | 200/18866 | 0.004758 | 0.026712 | 0.009819 | MAPK3,NOX4 | 2 |
| BP | GO:1901654 | response to ketone | 2月10日 | 200/18866 | 0.004758 | 0.026712 | 0.009819 | SREBF1,DUSP1 | 2 |
| BP | GO:0001763 | morphogenesis of a branching structure | 2月10日 | 201/18866 | 0.004805 | 0.026738 | 0.009829 | TNF,KRAS | 2 |
| BP | GO:0010952 | positive regulation of peptidase activity | 2月10日 | 201/18866 | 0.004805 | 0.026738 | 0.009829 | TNF,MAPK3 | 2 |
| BP | GO:1903708 | positive regulation of hemopoiesis | 2月10日 | 204/18866 | 0.004945 | 0.027402 | 0.010073 | TNF,XBP1 | 2 |
| BP | GO:0002377 | immunoglobulin production | 2月10日 | 210/18866 | 0.005232 | 0.028543 | 0.010493 | TNF,XBP1 | 2 |
| BP | GO:0001660 | fever generation | 1月10日 | 10/18866 | 0.005289 | 0.028543 | 0.010493 | TNF | 1 |
| BP | GO:0002676 | regulation of chronic inflammatory response | 1月10日 | 10/18866 | 0.005289 | 0.028543 | 0.010493 | TNF | 1 |
| BP | GO:0036500 | ATF6-mediated unfolded protein response | 1月10日 | 10/18866 | 0.005289 | 0.028543 | 0.010493 | XBP1 | 1 |
| BP | GO:0045348 | positive regulation of MHC class II biosynthetic process | 1月10日 | 10/18866 | 0.005289 | 0.028543 | 0.010493 | XBP1 | 1 |
| BP | GO:1900222 | negative regulation of amyloid-beta clearance | 1月10日 | 10/18866 | 0.005289 | 0.028543 | 0.010493 | TNF | 1 |
| BP | GO:0006643 | membrane lipid metabolic process | 2月10日 | 212/18866 | 0.00533 | 0.028543 | 0.010493 | TNF,SIRT3 | 2 |
| BP | GO:0030278 | regulation of ossification | 2月10日 | 212/18866 | 0.00533 | 0.028543 | 0.010493 | TNF,MAPK3 | 2 |
| BP | GO:0006006 | glucose metabolic process | 2月10日 | 214/18866 | 0.005428 | 0.028948 | 0.010641 | TNF,G6PD | 2 |
| BP | GO:0002064 | epithelial cell development | 2月10日 | 221/18866 | 0.005778 | 0.029844 | 0.010971 | TNF,XBP1 | 2 |
| BP | GO:0031652 | positive regulation of heat generation | 1月10日 | 11/18866 | 0.005817 | 0.029844 | 0.010971 | TNF | 1 |
| BP | GO:0042789 | mRNA transcription by RNA polymerase II | 1月10日 | 11/18866 | 0.005817 | 0.029844 | 0.010971 | SREBF1 | 1 |
| BP | GO:0051024 | positive regulation of immunoglobulin secretion | 1月10日 | 11/18866 | 0.005817 | 0.029844 | 0.010971 | XBP1 | 1 |
| BP | GO:0060439 | trachea morphogenesis | 1月10日 | 11/18866 | 0.005817 | 0.029844 | 0.010971 | MAPK3 | 1 |
| BP | GO:0061307 | cardiac neural crest cell differentiation involved in heart development | 1月10日 | 11/18866 | 0.005817 | 0.029844 | 0.010971 | MAPK3 | 1 |
| BP | GO:0061308 | cardiac neural crest cell development involved in heart development | 1月10日 | 11/18866 | 0.005817 | 0.029844 | 0.010971 | MAPK3 | 1 |
| BP | GO:0071803 | positive regulation of podosome assembly | 1月10日 | 11/18866 | 0.005817 | 0.029844 | 0.010971 | TNF | 1 |
| BP | GO:0090205 | positive regulation of cholesterol metabolic process | 1月10日 | 11/18866 | 0.005817 | 0.029844 | 0.010971 | SREBF1 | 1 |
| BP | GO:0097529 | myeloid leukocyte migration | 2月10日 | 222/18866 | 0.005829 | 0.029844 | 0.010971 | MAPK3,DUSP1 | 2 |
| BP | GO:0002699 | positive regulation of immune effector process | 2月10日 | 223/18866 | 0.00588 | 0.029986 | 0.011023 | TNF,XBP1 | 2 |
| BP | GO:0032869 | cellular response to insulin stimulus | 2月10日 | 226/18866 | 0.006035 | 0.030531 | 0.011223 | SREBF1,XBP1 | 2 |
| BP | GO:0044706 | multi-multicellular organism process | 2月10日 | 226/18866 | 0.006035 | 0.030531 | 0.011223 | MAPK3,KRAS | 2 |
| BP | GO:0050920 | regulation of chemotaxis | 2月10日 | 229/18866 | 0.006191 | 0.030901 | 0.011359 | MAPK3,DUSP1 | 2 |
| BP | GO:0048738 | cardiac muscle tissue development | 2月10日 | 231/18866 | 0.006296 | 0.030901 | 0.011359 | NOX4,G6PD | 2 |
| BP | GO:0000185 | activation of MAPKKK activity | 1月10日 | 12/18866 | 0.006344 | 0.030901 | 0.011359 | TNF | 1 |
| BP | GO:0002863 | positive regulation of inflammatory response to antigenic stimulus | 1月10日 | 12/18866 | 0.006344 | 0.030901 | 0.011359 | TNF | 1 |
| BP | GO:0002923 | regulation of humoral immune response mediated by circulating immunoglobulin | 1月10日 | 12/18866 | 0.006344 | 0.030901 | 0.011359 | TNF | 1 |
| BP | GO:0042368 | vitamin D biosynthetic process | 1月10日 | 12/18866 | 0.006344 | 0.030901 | 0.011359 | TNF | 1 |
| BP | GO:0051798 | positive regulation of hair follicle development | 1月10日 | 12/18866 | 0.006344 | 0.030901 | 0.011359 | TNF | 1 |
| BP | GO:0061517 | macrophage proliferation | 1月10日 | 12/18866 | 0.006344 | 0.030901 | 0.011359 | MAPK3 | 1 |
| BP | GO:0072584 | caveolin-mediated endocytosis | 1月10日 | 12/18866 | 0.006344 | 0.030901 | 0.011359 | MAPK3 | 1 |
| BP | GO:0030595 | leukocyte chemotaxis | 2月10日 | 232/18866 | 0.006349 | 0.030901 | 0.011359 | MAPK3,DUSP1 | 2 |
| BP | GO:2001234 | negative regulation of apoptotic signaling pathway | 2月10日 | 233/18866 | 0.006402 | 0.031042 | 0.011411 | TNF,XBP1 | 2 |
| BP | GO:0071695 | anatomical structure maturation | 2月10日 | 235/18866 | 0.006509 | 0.031323 | 0.011514 | G6PD,XBP1 | 2 |
| BP | GO:1903039 | positive regulation of leukocyte cell-cell adhesion | 2月10日 | 235/18866 | 0.006509 | 0.031323 | 0.011514 | TNF,XBP1 | 2 |
| BP | GO:0030656 | regulation of vitamin metabolic process | 1月10日 | 13/18866 | 0.006871 | 0.032216 | 0.011842 | TNF | 1 |
| BP | GO:0031650 | regulation of heat generation | 1月10日 | 13/18866 | 0.006871 | 0.032216 | 0.011842 | TNF | 1 |
| BP | GO:0033127 | regulation of histone phosphorylation | 1月10日 | 13/18866 | 0.006871 | 0.032216 | 0.011842 | MAPK3 | 1 |
| BP | GO:0042635 | positive regulation of hair cycle | 1月10日 | 13/18866 | 0.006871 | 0.032216 | 0.011842 | TNF | 1 |
| BP | GO:0045579 | positive regulation of B cell differentiation | 1月10日 | 13/18866 | 0.006871 | 0.032216 | 0.011842 | XBP1 | 1 |
| BP | GO:0061052 | negative regulation of cell growth involved in cardiac muscle cell development | 1月10日 | 13/18866 | 0.006871 | 0.032216 | 0.011842 | G6PD | 1 |
| BP | GO:1900103 | positive regulation of endoplasmic reticulum unfolded protein response | 1月10日 | 13/18866 | 0.006871 | 0.032216 | 0.011842 | XBP1 | 1 |
| BP | GO:0042593 | glucose homeostasis | 2月10日 | 245/18866 | 0.007056 | 0.032964 | 0.012117 | NOX4,XBP1 | 2 |
| BP | GO:0033500 | carbohydrate homeostasis | 2月10日 | 246/18866 | 0.007112 | 0.033104 | 0.012169 | NOX4,XBP1 | 2 |
| BP | GO:0002833 | positive regulation of response to biotic stimulus | 2月10日 | 251/18866 | 0.007394 | 0.03346 | 0.0123 | MAPK3,KRAS | 2 |
| BP | GO:0032042 | mitochondrial DNA metabolic process | 1月10日 | 14/18866 | 0.007398 | 0.03346 | 0.0123 | LONP1 | 1 |
| BP | GO:0032933 | SREBP signaling pathway | 1月10日 | 14/18866 | 0.007398 | 0.03346 | 0.0123 | SREBF1 | 1 |
| BP | GO:0050667 | homocysteine metabolic process | 1月10日 | 14/18866 | 0.007398 | 0.03346 | 0.0123 | NOX4 | 1 |
| BP | GO:0090231 | regulation of spindle checkpoint | 1月10日 | 14/18866 | 0.007398 | 0.03346 | 0.0123 | DUSP1 | 1 |
| BP | GO:0090266 | regulation of mitotic cell cycle spindle assembly checkpoint | 1月10日 | 14/18866 | 0.007398 | 0.03346 | 0.0123 | DUSP1 | 1 |
| BP | GO:1903504 | regulation of mitotic spindle checkpoint | 1月10日 | 14/18866 | 0.007398 | 0.03346 | 0.0123 | DUSP1 | 1 |
| BP | GO:1903799 | negative regulation of production of miRNAs involved in gene silencing by miRNA | 1月10日 | 14/18866 | 0.007398 | 0.03346 | 0.0123 | TNF | 1 |
| BP | GO:0051896 | regulation of protein kinase B signaling | 2月10日 | 253/18866 | 0.007509 | 0.033698 | 0.012387 | TNF,NOX4 | 2 |
| BP | GO:0019318 | hexose metabolic process | 2月10日 | 254/18866 | 0.007566 | 0.033698 | 0.012387 | TNF,G6PD | 2 |
| BP | GO:2000027 | regulation of animal organ morphogenesis | 2月10日 | 254/18866 | 0.007566 | 0.033698 | 0.012387 | TNF,XBP1 | 2 |
| BP | GO:0048872 | homeostasis of number of cells | 2月10日 | 256/18866 | 0.007682 | 0.033698 | 0.012387 | G6PD,KRAS | 2 |
| BP | GO:0010867 | positive regulation of triglyceride biosynthetic process | 1月10日 | 15/18866 | 0.007924 | 0.033698 | 0.012387 | SREBF1 | 1 |
| BP | GO:0019321 | pentose metabolic process | 1月10日 | 15/18866 | 0.007924 | 0.033698 | 0.012387 | G6PD | 1 |
| BP | GO:0042362 | fat-soluble vitamin biosynthetic process | 1月10日 | 15/18866 | 0.007924 | 0.033698 | 0.012387 | TNF | 1 |
| BP | GO:0045346 | regulation of MHC class II biosynthetic process | 1月10日 | 15/18866 | 0.007924 | 0.033698 | 0.012387 | XBP1 | 1 |
| BP | GO:0048308 | organelle inheritance | 1月10日 | 15/18866 | 0.007924 | 0.033698 | 0.012387 | MAPK3 | 1 |
| BP | GO:0048313 | Golgi inheritance | 1月10日 | 15/18866 | 0.007924 | 0.033698 | 0.012387 | MAPK3 | 1 |
| BP | GO:0051044 | positive regulation of membrane protein ectodomain proteolysis | 1月10日 | 15/18866 | 0.007924 | 0.033698 | 0.012387 | TNF | 1 |
| BP | GO:0071501 | cellular response to sterol depletion | 1月10日 | 15/18866 | 0.007924 | 0.033698 | 0.012387 | SREBF1 | 1 |
| BP | GO:0071801 | regulation of podosome assembly | 1月10日 | 15/18866 | 0.007924 | 0.033698 | 0.012387 | TNF | 1 |
| BP | GO:0072567 | chemokine (C-X-C motif) ligand 2 production | 1月10日 | 15/18866 | 0.007924 | 0.033698 | 0.012387 | TNF | 1 |
| BP | GO:1901550 | regulation of endothelial cell development | 1月10日 | 15/18866 | 0.007924 | 0.033698 | 0.012387 | TNF | 1 |
| BP | GO:1903140 | regulation of establishment of endothelial barrier | 1月10日 | 15/18866 | 0.007924 | 0.033698 | 0.012387 | TNF | 1 |
| BP | GO:2000341 | regulation of chemokine (C-X-C motif) ligand 2 production | 1月10日 | 15/18866 | 0.007924 | 0.033698 | 0.012387 | TNF | 1 |
| BP | GO:2000345 | regulation of hepatocyte proliferation | 1月10日 | 15/18866 | 0.007924 | 0.033698 | 0.012387 | XBP1 | 1 |
| BP | GO:0001894 | tissue homeostasis | 2月10日 | 261/18866 | 0.007974 | 0.033796 | 0.012424 | NOX4,KRAS | 2 |
| BP | GO:0061448 | connective tissue development | 2月10日 | 262/18866 | 0.008033 | 0.033934 | 0.012474 | MAPK3,XBP1 | 2 |
| BP | GO:0050730 | regulation of peptidyl-tyrosine phosphorylation | 2月10日 | 263/18866 | 0.008092 | 0.034072 | 0.012525 | TNF,NOX4 | 2 |
| BP | GO:0002070 | epithelial cell maturation | 1月10日 | 16/18866 | 0.008451 | 0.03423 | 0.012583 | XBP1 | 1 |
| BP | GO:0006098 | pentose-phosphate shunt | 1月10日 | 16/18866 | 0.008451 | 0.03423 | 0.012583 | G6PD | 1 |
| BP | GO:0034116 | positive regulation of heterotypic cell-cell adhesion | 1月10日 | 16/18866 | 0.008451 | 0.03423 | 0.012583 | TNF | 1 |
| BP | GO:0043249 | erythrocyte maturation | 1月10日 | 16/18866 | 0.008451 | 0.03423 | 0.012583 | G6PD | 1 |
| BP | GO:0045342 | MHC class II biosynthetic process | 1月10日 | 16/18866 | 0.008451 | 0.03423 | 0.012583 | XBP1 | 1 |
| BP | GO:0046325 | negative regulation of glucose import | 1月10日 | 16/18866 | 0.008451 | 0.03423 | 0.012583 | TNF | 1 |
| BP | GO:0051447 | negative regulation of meiotic cell cycle | 1月10日 | 16/18866 | 0.008451 | 0.03423 | 0.012583 | DUSP1 | 1 |
| BP | GO:0070886 | positive regulation of calcineurin-NFAT signaling cascade | 1月10日 | 16/18866 | 0.008451 | 0.03423 | 0.012583 | TNF | 1 |
| BP | GO:0106058 | positive regulation of calcineurin-mediated signaling | 1月10日 | 16/18866 | 0.008451 | 0.03423 | 0.012583 | TNF | 1 |
| BP | GO:1902514 | regulation of calcium ion transmembrane transport via high voltage-gated calcium channel | 1月10日 | 16/18866 | 0.008451 | 0.03423 | 0.012583 | G6PD | 1 |
| BP | GO:1903358 | regulation of Golgi organization | 1月10日 | 16/18866 | 0.008451 | 0.03423 | 0.012583 | MAPK3 | 1 |
| BP | GO:1905050 | positive regulation of metallopeptidase activity | 1月10日 | 16/18866 | 0.008451 | 0.03423 | 0.012583 | MAPK3 | 1 |
| BP | GO:0051091 | positive regulation of DNA-binding transcription factor activity | 2月10日 | 270/18866 | 0.008513 | 0.034373 | 0.012635 | TNF,KRAS | 2 |
| BP | GO:0006991 | response to sterol depletion | 1月10日 | 17/18866 | 0.008977 | 0.035138 | 0.012917 | SREBF1 | 1 |
| BP | GO:0009299 | mRNA transcription | 1月10日 | 17/18866 | 0.008977 | 0.035138 | 0.012917 | SREBF1 | 1 |
| BP | GO:0030730 | sequestering of triglyceride | 1月10日 | 17/18866 | 0.008977 | 0.035138 | 0.012917 | TNF | 1 |
| BP | GO:0031065 | positive regulation of histone deacetylation | 1月10日 | 17/18866 | 0.008977 | 0.035138 | 0.012917 | SREBF1 | 1 |
| BP | GO:0031649 | heat generation | 1月10日 | 17/18866 | 0.008977 | 0.035138 | 0.012917 | TNF | 1 |
| BP | GO:0035020 | regulation of Rac protein signal transduction | 1月10日 | 17/18866 | 0.008977 | 0.035138 | 0.012917 | KRAS | 1 |
| BP | GO:0055089 | fatty acid homeostasis | 1月10日 | 17/18866 | 0.008977 | 0.035138 | 0.012917 | XBP1 | 1 |
| BP | GO:0071850 | mitotic cell cycle arrest | 1月10日 | 17/18866 | 0.008977 | 0.035138 | 0.012917 | DUSP1 | 1 |
| BP | GO:1904355 | positive regulation of telomere capping | 1月10日 | 17/18866 | 0.008977 | 0.035138 | 0.012917 | MAPK3 | 1 |
| BP | GO:1905331 | negative regulation of morphogenesis of an epithelium | 1月10日 | 17/18866 | 0.008977 | 0.035138 | 0.012917 | TNF | 1 |
| BP | GO:0043491 | protein kinase B signaling | 2月10日 | 278/18866 | 0.009005 | 0.035142 | 0.012918 | TNF,NOX4 | 2 |
| BP | GO:0022409 | positive regulation of cell-cell adhesion | 2月10日 | 279/18866 | 0.009068 | 0.035278 | 0.012968 | TNF,XBP1 | 2 |
| BP | GO:0032868 | response to insulin | 2月10日 | 283/18866 | 0.009319 | 0.036148 | 0.013288 | SREBF1,XBP1 | 2 |
| BP | GO:0006740 | NADPH regeneration | 1月10日 | 18/18866 | 0.009502 | 0.0362 | 0.013307 | G6PD | 1 |
| BP | GO:0010832 | negative regulation of myotube differentiation | 1月10日 | 18/18866 | 0.009502 | 0.0362 | 0.013307 | XBP1 | 1 |
| BP | GO:0061577 | calcium ion transmembrane transport via high voltage-gated calcium channel | 1月10日 | 18/18866 | 0.009502 | 0.0362 | 0.013307 | G6PD | 1 |
| BP | GO:0150078 | positive regulation of neuroinflammatory response | 1月10日 | 18/18866 | 0.009502 | 0.0362 | 0.013307 | TNF | 1 |
| BP | GO:1900221 | regulation of amyloid-beta clearance | 1月10日 | 18/18866 | 0.009502 | 0.0362 | 0.013307 | TNF | 1 |
| BP | GO:2000641 | regulation of early endosome to late endosome transport | 1月10日 | 18/18866 | 0.009502 | 0.0362 | 0.013307 | MAPK3 | 1 |
| BP | GO:0021700 | developmental maturation | 2月10日 | 287/18866 | 0.009574 | 0.036365 | 0.013368 | G6PD,XBP1 | 2 |
| BP | GO:0097193 | intrinsic apoptotic signaling pathway | 2月10日 | 290/18866 | 0.009768 | 0.03688 | 0.013557 | TNF,XBP1 | 2 |
| BP | GO:1902105 | regulation of leukocyte differentiation | 2月10日 | 290/18866 | 0.009768 | 0.03688 | 0.013557 | TNF,XBP1 | 2 |
| BP | GO:0002922 | positive regulation of humoral immune response | 1月10日 | 19/18866 | 0.010028 | 0.037205 | 0.013676 | TNF | 1 |
| BP | GO:0010759 | positive regulation of macrophage chemotaxis | 1月10日 | 19/18866 | 0.010028 | 0.037205 | 0.013676 | MAPK3 | 1 |
| BP | GO:0060252 | positive regulation of glial cell proliferation | 1月10日 | 19/18866 | 0.010028 | 0.037205 | 0.013676 | TNF | 1 |
| BP | GO:0060438 | trachea development | 1月10日 | 19/18866 | 0.010028 | 0.037205 | 0.013676 | MAPK3 | 1 |
| BP | GO:0071800 | podosome assembly | 1月10日 | 19/18866 | 0.010028 | 0.037205 | 0.013676 | TNF | 1 |
| BP | GO:2000010 | positive regulation of protein localization to cell surface | 1月10日 | 19/18866 | 0.010028 | 0.037205 | 0.013676 | TNF | 1 |
| BP | GO:0005996 | monosaccharide metabolic process | 2月10日 | 296/18866 | 0.010159 | 0.037584 | 0.013816 | TNF,G6PD | 2 |
| BP | GO:0003015 | heart process | 2月10日 | 297/18866 | 0.010225 | 0.03761 | 0.013826 | SREBF1,NOX4 | 2 |
| BP | GO:0071356 | cellular response to tumor necrosis factor | 2月10日 | 297/18866 | 0.010225 | 0.03761 | 0.013826 | TNF,MAPK3 | 2 |
| BP | GO:0002544 | chronic inflammatory response | 1月10日 | 20/18866 | 0.010553 | 0.037855 | 0.013916 | TNF | 1 |
| BP | GO:0002689 | negative regulation of leukocyte chemotaxis | 1月10日 | 20/18866 | 0.010553 | 0.037855 | 0.013916 | DUSP1 | 1 |
| BP | GO:0007252 | I-kappaB phosphorylation | 1月10日 | 20/18866 | 0.010553 | 0.037855 | 0.013916 | TNF | 1 |
| BP | GO:0060965 | negative regulation of gene silencing by miRNA | 1月10日 | 20/18866 | 0.010553 | 0.037855 | 0.013916 | TNF | 1 |
| BP | GO:0070262 | peptidyl-serine dephosphorylation | 1月10日 | 20/18866 | 0.010553 | 0.037855 | 0.013916 | DUSP1 | 1 |
| BP | GO:1902004 | positive regulation of amyloid-beta formation | 1月10日 | 20/18866 | 0.010553 | 0.037855 | 0.013916 | TNF | 1 |
| BP | GO:1902236 | negative regulation of endoplasmic reticulum stress-induced intrinsic apoptotic signaling pathway | 1月10日 | 20/18866 | 0.010553 | 0.037855 | 0.013916 | XBP1 | 1 |
| BP | GO:2000774 | positive regulation of cellular senescence | 1月10日 | 20/18866 | 0.010553 | 0.037855 | 0.013916 | KRAS | 1 |
| BP | GO:0050890 | cognition | 2月10日 | 302/18866 | 0.010558 | 0.037855 | 0.013916 | TNF,KRAS | 2 |
| BP | GO:0002440 | production of molecular mediator of immune response | 2月10日 | 306/18866 | 0.010828 | 0.038714 | 0.014231 | TNF,XBP1 | 2 |
| BP | GO:0009110 | vitamin biosynthetic process | 1月10日 | 21/18866 | 0.011078 | 0.038822 | 0.014271 | TNF | 1 |
| BP | GO:0010829 | negative regulation of glucose transmembrane transport | 1月10日 | 21/18866 | 0.011078 | 0.038822 | 0.014271 | TNF | 1 |
| BP | GO:0030878 | thyroid gland development | 1月10日 | 21/18866 | 0.011078 | 0.038822 | 0.014271 | MAPK3 | 1 |
| BP | GO:0051797 | regulation of hair follicle development | 1月10日 | 21/18866 | 0.011078 | 0.038822 | 0.014271 | TNF | 1 |
| BP | GO:0072574 | hepatocyte proliferation | 1月10日 | 21/18866 | 0.011078 | 0.038822 | 0.014271 | XBP1 | 1 |
| BP | GO:0072575 | epithelial cell proliferation involved in liver morphogenesis | 1月10日 | 21/18866 | 0.011078 | 0.038822 | 0.014271 | XBP1 | 1 |
| BP | GO:0090312 | positive regulation of protein deacetylation | 1月10日 | 21/18866 | 0.011078 | 0.038822 | 0.014271 | SREBF1 | 1 |
| BP | GO:0018105 | peptidyl-serine phosphorylation | 2月10日 | 310/18866 | 0.011101 | 0.038822 | 0.014271 | TNF,MAPK3 | 2 |
| BP | GO:0060326 | cell chemotaxis | 2月10日 | 311/18866 | 0.011169 | 0.038956 | 0.01432 | MAPK3,DUSP1 | 2 |
| BP | GO:0002313 | mature B cell differentiation involved in immune response | 1月10日 | 22/18866 | 0.011603 | 0.039803 | 0.014632 | XBP1 | 1 |
| BP | GO:0010888 | negative regulation of lipid storage | 1月10日 | 22/18866 | 0.011603 | 0.039803 | 0.014632 | TNF | 1 |
| BP | GO:0035970 | peptidyl-threonine dephosphorylation | 1月10日 | 22/18866 | 0.011603 | 0.039803 | 0.014632 | DUSP1 | 1 |
| BP | GO:0042359 | vitamin D metabolic process | 1月10日 | 22/18866 | 0.011603 | 0.039803 | 0.014632 | TNF | 1 |
| BP | GO:0051131 | chaperone-mediated protein complex assembly | 1月10日 | 22/18866 | 0.011603 | 0.039803 | 0.014632 | LONP1 | 1 |
| BP | GO:0072576 | liver morphogenesis | 1月10日 | 22/18866 | 0.011603 | 0.039803 | 0.014632 | XBP1 | 1 |
| BP | GO:0009416 | response to light stimulus | 2月10日 | 319/18866 | 0.011726 | 0.039803 | 0.014632 | KRAS,DUSP1 | 2 |
| BP | GO:0034612 | response to tumor necrosis factor | 2月10日 | 320/18866 | 0.011796 | 0.039803 | 0.014632 | TNF,MAPK3 | 2 |
| BP | GO:0006470 | protein dephosphorylation | 2月10日 | 323/18866 | 0.012009 | 0.039803 | 0.014632 | TNF,DUSP1 | 2 |
| BP | GO:0000002 | mitochondrial genome maintenance | 1月10日 | 23/18866 | 0.012127 | 0.039803 | 0.014632 | LONP1 | 1 |
| BP | GO:0010866 | regulation of triglyceride biosynthetic process | 1月10日 | 23/18866 | 0.012127 | 0.039803 | 0.014632 | SREBF1 | 1 |
| BP | GO:0031281 | positive regulation of cyclase activity | 1月10日 | 23/18866 | 0.012127 | 0.039803 | 0.014632 | MAPK3 | 1 |
| BP | GO:0051043 | regulation of membrane protein ectodomain proteolysis | 1月10日 | 23/18866 | 0.012127 | 0.039803 | 0.014632 | TNF | 1 |
| BP | GO:0060149 | negative regulation of posttranscriptional gene silencing | 1月10日 | 23/18866 | 0.012127 | 0.039803 | 0.014632 | TNF | 1 |
| BP | GO:0060445 | branching involved in salivary gland morphogenesis | 1月10日 | 23/18866 | 0.012127 | 0.039803 | 0.014632 | TNF | 1 |
| BP | GO:0060967 | negative regulation of gene silencing by RNA | 1月10日 | 23/18866 | 0.012127 | 0.039803 | 0.014632 | TNF | 1 |
| BP | GO:0090343 | positive regulation of cell aging | 1月10日 | 23/18866 | 0.012127 | 0.039803 | 0.014632 | KRAS | 1 |
| BP | GO:1900017 | positive regulation of cytokine production involved in inflammatory response | 1月10日 | 23/18866 | 0.012127 | 0.039803 | 0.014632 | TNF | 1 |
| BP | GO:1901522 | positive regulation of transcription from RNA polymerase II promoter involved in cellular response to chemical stimulus | 1月10日 | 23/18866 | 0.012127 | 0.039803 | 0.014632 | XBP1 | 1 |
| BP | GO:1903798 | regulation of production of miRNAs involved in gene silencing by miRNA | 1月10日 | 23/18866 | 0.012127 | 0.039803 | 0.014632 | TNF | 1 |
| BP | GO:1904996 | positive regulation of leukocyte adhesion to vascular endothelial cell | 1月10日 | 23/18866 | 0.012127 | 0.039803 | 0.014632 | TNF | 1 |
| BP | GO:2000726 | negative regulation of cardiac muscle cell differentiation | 1月10日 | 23/18866 | 0.012127 | 0.039803 | 0.014632 | G6PD | 1 |
| BP | GO:2000810 | regulation of bicellular tight junction assembly | 1月10日 | 23/18866 | 0.012127 | 0.039803 | 0.014632 | TNF | 1 |
| BP | GO:1903037 | regulation of leukocyte cell-cell adhesion | 2月10日 | 329/18866 | 0.012438 | 0.040486 | 0.014882 | TNF,XBP1 | 2 |
| BP | GO:0071375 | cellular response to peptide hormone stimulus | 2月10日 | 330/18866 | 0.012511 | 0.040486 | 0.014882 | SREBF1,XBP1 | 2 |
| BP | GO:0032147 | activation of protein kinase activity | 2月10日 | 331/18866 | 0.012583 | 0.040486 | 0.014882 | TNF,MAPK3 | 2 |
| BP | GO:0060562 | epithelial tube morphogenesis | 2月10日 | 331/18866 | 0.012583 | 0.040486 | 0.014882 | TNF,KRAS | 2 |
| BP | GO:0071214 | cellular response to abiotic stimulus | 2月10日 | 331/18866 | 0.012583 | 0.040486 | 0.014882 | MAPK3,NOX4 | 2 |
| BP | GO:0104004 | cellular response to environmental stimulus | 2月10日 | 331/18866 | 0.012583 | 0.040486 | 0.014882 | MAPK3,NOX4 | 2 |
| BP | GO:0002719 | negative regulation of cytokine production involved in immune response | 1月10日 | 24/18866 | 0.012652 | 0.040486 | 0.014882 | TNF | 1 |
| BP | GO:0050995 | negative regulation of lipid catabolic process | 1月10日 | 24/18866 | 0.012652 | 0.040486 | 0.014882 | TNF | 1 |
| BP | GO:0090208 | positive regulation of triglyceride metabolic process | 1月10日 | 24/18866 | 0.012652 | 0.040486 | 0.014882 | SREBF1 | 1 |
| BP | GO:1902993 | positive regulation of amyloid precursor protein catabolic process | 1月10日 | 24/18866 | 0.012652 | 0.040486 | 0.014882 | TNF | 1 |
| BP | GO:0018209 | peptidyl-serine modification | 2月10日 | 333/18866 | 0.012729 | 0.04063 | 0.014936 | TNF,MAPK3 | 2 |
| BP | GO:0045662 | negative regulation of myoblast differentiation | 1月10日 | 25/18866 | 0.013176 | 0.041437 | 0.015232 | TNF | 1 |
| BP | GO:0046697 | decidualization | 1月10日 | 25/18866 | 0.013176 | 0.041437 | 0.015232 | MAPK3 | 1 |
| BP | GO:0048169 | regulation of long-term neuronal synaptic plasticity | 1月10日 | 25/18866 | 0.013176 | 0.041437 | 0.015232 | KRAS | 1 |
| BP | GO:0061050 | regulation of cell growth involved in cardiac muscle cell development | 1月10日 | 25/18866 | 0.013176 | 0.041437 | 0.015232 | G6PD | 1 |
| BP | GO:0070920 | regulation of production of small RNA involved in gene silencing by RNA | 1月10日 | 25/18866 | 0.013176 | 0.041437 | 0.015232 | TNF | 1 |
| BP | GO:0071677 | positive regulation of mononuclear cell migration | 1月10日 | 25/18866 | 0.013176 | 0.041437 | 0.015232 | TNF | 1 |
| BP | GO:0045930 | negative regulation of mitotic cell cycle | 2月10日 | 341/18866 | 0.013318 | 0.041783 | 0.015359 | TNF,DUSP1 | 2 |
| BP | GO:0010721 | negative regulation of cell development | 2月10日 | 343/18866 | 0.013468 | 0.042148 | 0.015494 | TNF,G6PD | 2 |
| BP | GO:0000188 | inactivation of MAPK activity | 1月10日 | 26/18866 | 0.0137 | 0.042152 | 0.015495 | DUSP1 | 1 |
| BP | GO:0034114 | regulation of heterotypic cell-cell adhesion | 1月10日 | 26/18866 | 0.0137 | 0.042152 | 0.015495 | TNF | 1 |
| BP | GO:0051156 | glucose 6-phosphate metabolic process | 1月10日 | 26/18866 | 0.0137 | 0.042152 | 0.015495 | G6PD | 1 |
| BP | GO:0090025 | regulation of monocyte chemotaxis | 1月10日 | 26/18866 | 0.0137 | 0.042152 | 0.015495 | DUSP1 | 1 |
| BP | GO:1903649 | regulation of cytoplasmic transport | 1月10日 | 26/18866 | 0.0137 | 0.042152 | 0.015495 | MAPK3 | 1 |
| BP | GO:1904353 | regulation of telomere capping | 1月10日 | 26/18866 | 0.0137 | 0.042152 | 0.015495 | MAPK3 | 1 |
| BP | GO:1905523 | positive regulation of macrophage migration | 1月10日 | 26/18866 | 0.0137 | 0.042152 | 0.015495 | MAPK3 | 1 |
| BP | GO:0030336 | negative regulation of cell migration | 2月10日 | 350/18866 | 0.013996 | 0.042962 | 0.015793 | TNF,DUSP1 | 2 |
| BP | GO:0009651 | response to salt stress | 1月10日 | 27/18866 | 0.014223 | 0.043243 | 0.015896 | TNF | 1 |
| BP | GO:0045672 | positive regulation of osteoclast differentiation | 1月10日 | 27/18866 | 0.014223 | 0.043243 | 0.015896 | TNF | 1 |
| BP | GO:0048143 | astrocyte activation | 1月10日 | 27/18866 | 0.014223 | 0.043243 | 0.015896 | TNF | 1 |
| BP | GO:1905208 | negative regulation of cardiocyte differentiation | 1月10日 | 27/18866 | 0.014223 | 0.043243 | 0.015896 | G6PD | 1 |
| BP | GO:0032386 | regulation of intracellular transport | 2月10日 | 358/18866 | 0.014611 | 0.044308 | 0.016288 | MAPK3,SREBF1 | 2 |
| BP | GO:0001666 | response to hypoxia | 2月10日 | 359/18866 | 0.014689 | 0.044308 | 0.016288 | NOX4,LONP1 | 2 |
| BP | GO:0002335 | mature B cell differentiation | 1月10日 | 28/18866 | 0.014746 | 0.044308 | 0.016288 | XBP1 | 1 |
| BP | GO:0032800 | receptor biosynthetic process | 1月10日 | 28/18866 | 0.014746 | 0.044308 | 0.016288 | TNF | 1 |
| BP | GO:1900101 | regulation of endoplasmic reticulum unfolded protein response | 1月10日 | 28/18866 | 0.014746 | 0.044308 | 0.016288 | XBP1 | 1 |
| BP | GO:0007159 | leukocyte cell-cell adhesion | 2月10日 | 364/18866 | 0.01508 | 0.045034 | 0.016554 | TNF,XBP1 | 2 |
| BP | GO:2000146 | negative regulation of cell motility | 2月10日 | 365/18866 | 0.015159 | 0.045034 | 0.016554 | TNF,DUSP1 | 2 |
| BP | GO:0006515 | protein quality control for misfolded or incompletely synthesized proteins | 1月10日 | 29/18866 | 0.015269 | 0.045034 | 0.016554 | LONP1 | 1 |
| BP | GO:0031063 | regulation of histone deacetylation | 1月10日 | 29/18866 | 0.015269 | 0.045034 | 0.016554 | SREBF1 | 1 |
| BP | GO:0042634 | regulation of hair cycle | 1月10日 | 29/18866 | 0.015269 | 0.045034 | 0.016554 | TNF | 1 |
| BP | GO:0045577 | regulation of B cell differentiation | 1月10日 | 29/18866 | 0.015269 | 0.045034 | 0.016554 | XBP1 | 1 |
| BP | GO:0048873 | homeostasis of number of cells within a tissue | 1月10日 | 29/18866 | 0.015269 | 0.045034 | 0.016554 | KRAS | 1 |
| BP | GO:0050901 | leukocyte tethering or rolling | 1月10日 | 29/18866 | 0.015269 | 0.045034 | 0.016554 | TNF | 1 |
| BP | GO:0045862 | positive regulation of proteolysis | 2月10日 | 370/18866 | 0.015555 | 0.045772 | 0.016826 | TNF,MAPK3 | 2 |
| BP | GO:0006790 | sulfur compound metabolic process | 2月10日 | 371/18866 | 0.015635 | 0.045797 | 0.016835 | NOX4,G6PD | 2 |
| BP | GO:0036293 | response to decreased oxygen levels | 2月10日 | 371/18866 | 0.015635 | 0.045797 | 0.016835 | NOX4,LONP1 | 2 |
| BP | GO:0010758 | regulation of macrophage chemotaxis | 1月10日 | 30/18866 | 0.015792 | 0.045941 | 0.016888 | MAPK3 | 1 |
| BP | GO:0035066 | positive regulation of histone acetylation | 1月10日 | 30/18866 | 0.015792 | 0.045941 | 0.016888 | MAPK3 | 1 |
| BP | GO:0071480 | cellular response to gamma radiation | 1月10日 | 30/18866 | 0.015792 | 0.045941 | 0.016888 | NOX4 | 1 |
| BP | GO:0002675 | positive regulation of acute inflammatory response | 1月10日 | 31/18866 | 0.016315 | 0.047246 | 0.017367 | TNF | 1 |
| BP | GO:0045948 | positive regulation of translational initiation | 1月10日 | 31/18866 | 0.016315 | 0.047246 | 0.017367 | TNF | 1 |
| BP | GO:0006909 | phagocytosis | 2月10日 | 382/18866 | 0.016526 | 0.04775 | 0.017553 | TNF,MAPK3 | 2 |
| BP | GO:0051385 | response to mineralocorticoid | 1月10日 | 32/18866 | 0.016837 | 0.048105 | 0.017683 | KRAS | 1 |
| BP | GO:0071353 | cellular response to interleukin-4 | 1月10日 | 32/18866 | 0.016837 | 0.048105 | 0.017683 | XBP1 | 1 |
| BP | GO:1901889 | negative regulation of cell junction assembly | 1月10日 | 32/18866 | 0.016837 | 0.048105 | 0.017683 | TNF | 1 |
| BP | GO:1901976 | regulation of cell cycle checkpoint | 1月10日 | 32/18866 | 0.016837 | 0.048105 | 0.017683 | DUSP1 | 1 |
| BP | GO:1902235 | regulation of endoplasmic reticulum stress-induced intrinsic apoptotic signaling pathway | 1月10日 | 32/18866 | 0.016837 | 0.048105 | 0.017683 | XBP1 | 1 |
| BP | GO:0014706 | striated muscle tissue development | 2月10日 | 389/18866 | 0.017104 | 0.04876 | 0.017924 | NOX4,G6PD | 2 |
| BP | GO:0031331 | positive regulation of cellular catabolic process | 2月10日 | 390/18866 | 0.017187 | 0.048834 | 0.017951 | TNF,MAPK3 | 2 |
| BP | GO:0002861 | regulation of inflammatory response to antigenic stimulus | 1月10日 | 33/18866 | 0.017359 | 0.048834 | 0.017951 | TNF | 1 |
| BP | GO:0010039 | response to iron ion | 1月10日 | 33/18866 | 0.017359 | 0.048834 | 0.017951 | G6PD | 1 |
| BP | GO:1902003 | regulation of amyloid-beta formation | 1月10日 | 33/18866 | 0.017359 | 0.048834 | 0.017951 | TNF | 1 |
| BP | GO:1905048 | regulation of metallopeptidase activity | 1月10日 | 33/18866 | 0.017359 | 0.048834 | 0.017951 | MAPK3 | 1 |
| BP | GO:2000758 | positive regulation of peptidyl-lysine acetylation | 1月10日 | 33/18866 | 0.017359 | 0.048834 | 0.017951 | MAPK3 | 1 |
| BP | GO:0050678 | regulation of epithelial cell proliferation | 2月10日 | 395/18866 | 0.017606 | 0.048904 | 0.017977 | TNF,XBP1 | 2 |
| BP | GO:0070482 | response to oxygen levels | 2月10日 | 396/18866 | 0.017691 | 0.048904 | 0.017977 | NOX4,LONP1 | 2 |
| BP | GO:0040013 | negative regulation of locomotion | 2月10日 | 397/18866 | 0.017775 | 0.048904 | 0.017977 | TNF,DUSP1 | 2 |
| BP | GO:1901653 | cellular response to peptide | 2月10日 | 398/18866 | 0.01786 | 0.048904 | 0.017977 | SREBF1,XBP1 | 2 |
| BP | GO:0007094 | mitotic spindle assembly checkpoint | 1月10日 | 34/18866 | 0.017881 | 0.048904 | 0.017977 | DUSP1 | 1 |
| BP | GO:0031577 | spindle checkpoint | 1月10日 | 34/18866 | 0.017881 | 0.048904 | 0.017977 | DUSP1 | 1 |
| BP | GO:0032212 | positive regulation of telomere maintenance via telomerase | 1月10日 | 34/18866 | 0.017881 | 0.048904 | 0.017977 | MAPK3 | 1 |
| BP | GO:0048566 | embryonic digestive tract development | 1月10日 | 34/18866 | 0.017881 | 0.048904 | 0.017977 | TNF | 1 |
| BP | GO:0050715 | positive regulation of cytokine secretion | 1月10日 | 34/18866 | 0.017881 | 0.048904 | 0.017977 | TNF | 1 |
| BP | GO:0055022 | negative regulation of cardiac muscle tissue growth | 1月10日 | 34/18866 | 0.017881 | 0.048904 | 0.017977 | G6PD | 1 |
| BP | GO:0061117 | negative regulation of heart growth | 1月10日 | 34/18866 | 0.017881 | 0.048904 | 0.017977 | G6PD | 1 |
| BP | GO:0071173 | spindle assembly checkpoint | 1月10日 | 34/18866 | 0.017881 | 0.048904 | 0.017977 | DUSP1 | 1 |
| BP | GO:0071174 | mitotic spindle checkpoint | 1月10日 | 34/18866 | 0.017881 | 0.048904 | 0.017977 | DUSP1 | 1 |
| BP | GO:0051271 | negative regulation of cellular component movement | 2月10日 | 400/18866 | 0.01803 | 0.049132 | 0.018061 | TNF,DUSP1 | 2 |
| BP | GO:0045765 | regulation of angiogenesis | 2月10日 | 403/18866 | 0.018286 | 0.049132 | 0.018061 | TNF,XBP1 | 2 |
| BP | GO:0006471 | protein ADP-ribosylation | 1月10日 | 35/18866 | 0.018402 | 0.049132 | 0.018061 | SIRT3 | 1 |
| BP | GO:0006739 | NADP metabolic process | 1月10日 | 35/18866 | 0.018402 | 0.049132 | 0.018061 | G6PD | 1 |
| BP | GO:0007435 | salivary gland morphogenesis | 1月10日 | 35/18866 | 0.018402 | 0.049132 | 0.018061 | TNF | 1 |
| BP | GO:0032228 | regulation of synaptic transmission, GABAergic | 1月10日 | 35/18866 | 0.018402 | 0.049132 | 0.018061 | KRAS | 1 |
| BP | GO:0048821 | erythrocyte development | 1月10日 | 35/18866 | 0.018402 | 0.049132 | 0.018061 | G6PD | 1 |
| BP | GO:0060251 | regulation of glial cell proliferation | 1月10日 | 35/18866 | 0.018402 | 0.049132 | 0.018061 | TNF | 1 |
| BP | GO:0070670 | response to interleukin-4 | 1月10日 | 35/18866 | 0.018402 | 0.049132 | 0.018061 | XBP1 | 1 |
| BP | GO:0070884 | regulation of calcineurin-NFAT signaling cascade | 1月10日 | 35/18866 | 0.018402 | 0.049132 | 0.018061 | TNF | 1 |
| BP | GO:0110111 | negative regulation of animal organ morphogenesis | 1月10日 | 35/18866 | 0.018402 | 0.049132 | 0.018061 | TNF | 1 |
| BP | GO:0018205 | peptidyl-lysine modification | 2月10日 | 405/18866 | 0.018458 | 0.049132 | 0.018061 | MAPK3,SIRT3 | 2 |
| BP | GO:0007517 | muscle organ development | 2月10日 | 407/18866 | 0.01863 | 0.049132 | 0.018061 | G6PD,XBP1 | 2 |
| BP | GO:0002831 | regulation of response to biotic stimulus | 2月10日 | 409/18866 | 0.018804 | 0.049132 | 0.018061 | MAPK3,KRAS | 2 |
| BP | GO:0060537 | muscle tissue development | 2月10日 | 409/18866 | 0.018804 | 0.049132 | 0.018061 | NOX4,G6PD | 2 |
| BP | GO:0001893 | maternal placenta development | 1月10日 | 36/18866 | 0.018923 | 0.049132 | 0.018061 | MAPK3 | 1 |
| BP | GO:0002701 | negative regulation of production of molecular mediator of immune response | 1月10日 | 36/18866 | 0.018923 | 0.049132 | 0.018061 | TNF | 1 |
| BP | GO:0033762 | response to glucagon | 1月10日 | 36/18866 | 0.018923 | 0.049132 | 0.018061 | SREBF1 | 1 |
| BP | GO:0042554 | superoxide anion generation | 1月10日 | 36/18866 | 0.018923 | 0.049132 | 0.018061 | NOX4 | 1 |
| BP | GO:0045841 | negative regulation of mitotic metaphase/anaphase transition | 1月10日 | 36/18866 | 0.018923 | 0.049132 | 0.018061 | DUSP1 | 1 |
| BP | GO:0046676 | negative regulation of insulin secretion | 1月10日 | 36/18866 | 0.018923 | 0.049132 | 0.018061 | SREBF1 | 1 |
| BP | GO:0051973 | positive regulation of telomerase activity | 1月10日 | 36/18866 | 0.018923 | 0.049132 | 0.018061 | MAPK3 | 1 |
| BP | GO:0106056 | regulation of calcineurin-mediated signaling | 1月10日 | 36/18866 | 0.018923 | 0.049132 | 0.018061 | TNF | 1 |
| BP | GO:1901099 | negative regulation of signal transduction in absence of ligand | 1月10日 | 36/18866 | 0.018923 | 0.049132 | 0.018061 | TNF | 1 |
| BP | GO:2001240 | negative regulation of extrinsic apoptotic signaling pathway in absence of ligand | 1月10日 | 36/18866 | 0.018923 | 0.049132 | 0.018061 | TNF | 1 |
| BP | GO:0001503 | ossification | 2月10日 | 412/18866 | 0.019065 | 0.049398 | 0.018159 | TNF,MAPK3 | 2 |
| BP | GO:2001233 | regulation of apoptotic signaling pathway | 2月10日 | 413/18866 | 0.019152 | 0.049524 | 0.018205 | TNF,XBP1 | 2 |
| BP | GO:0010614 | negative regulation of cardiac muscle hypertrophy | 1月10日 | 37/18866 | 0.019444 | 0.049579 | 0.018225 | G6PD | 1 |
| BP | GO:0043243 | positive regulation of protein-containing complex disassembly | 1月10日 | 37/18866 | 0.019444 | 0.049579 | 0.018225 | TNF | 1 |
| BP | GO:0060969 | negative regulation of gene silencing | 1月10日 | 37/18866 | 0.019444 | 0.049579 | 0.018225 | TNF | 1 |
| BP | GO:0097242 | amyloid-beta clearance | 1月10日 | 37/18866 | 0.019444 | 0.049579 | 0.018225 | TNF | 1 |
| BP | GO:1902100 | negative regulation of metaphase/anaphase transition of cell cycle | 1月10日 | 37/18866 | 0.019444 | 0.049579 | 0.018225 | DUSP1 | 1 |
| BP | GO:1904358 | positive regulation of telomere maintenance via telomere lengthening | 1月10日 | 37/18866 | 0.019444 | 0.049579 | 0.018225 | MAPK3 | 1 |
| BP | GO:1904994 | regulation of leukocyte adhesion to vascular endothelial cell | 1月10日 | 37/18866 | 0.019444 | 0.049579 | 0.018225 | TNF | 1 |
| BP | GO:0003298 | physiological muscle hypertrophy | 1月10日 | 38/18866 | 0.019965 | 0.050306 | 0.018492 | G6PD | 1 |
| BP | GO:0003301 | physiological cardiac muscle hypertrophy | 1月10日 | 38/18866 | 0.019965 | 0.050306 | 0.018492 | G6PD | 1 |
| BP | GO:0006361 | transcription initiation from RNA polymerase I promoter | 1月10日 | 38/18866 | 0.019965 | 0.050306 | 0.018492 | MAPK3 | 1 |
| BP | GO:0061049 | cell growth involved in cardiac muscle cell development | 1月10日 | 38/18866 | 0.019965 | 0.050306 | 0.018492 | G6PD | 1 |
| BP | GO:0071276 | cellular response to cadmium ion | 1月10日 | 38/18866 | 0.019965 | 0.050306 | 0.018492 | MAPK3 | 1 |
| BP | GO:1905898 | positive regulation of response to endoplasmic reticulum stress | 1月10日 | 38/18866 | 0.019965 | 0.050306 | 0.018492 | XBP1 | 1 |
| BP | GO:0045785 | positive regulation of cell adhesion | 2月10日 | 428/18866 | 0.020483 | 0.050621 | 0.018608 | TNF,XBP1 | 2 |
| BP | GO:0014741 | negative regulation of muscle hypertrophy | 1月10日 | 39/18866 | 0.020486 | 0.050621 | 0.018608 | G6PD | 1 |
| BP | GO:0016601 | Rac protein signal transduction | 1月10日 | 39/18866 | 0.020486 | 0.050621 | 0.018608 | KRAS | 1 |
| BP | GO:0030866 | cortical actin cytoskeleton organization | 1月10日 | 39/18866 | 0.020486 | 0.050621 | 0.018608 | TNF | 1 |
| BP | GO:0034205 | amyloid-beta formation | 1月10日 | 39/18866 | 0.020486 | 0.050621 | 0.018608 | TNF | 1 |
| BP | GO:0045022 | early endosome to late endosome transport | 1月10日 | 39/18866 | 0.020486 | 0.050621 | 0.018608 | MAPK3 | 1 |
| BP | GO:0055090 | acylglycerol homeostasis | 1月10日 | 39/18866 | 0.020486 | 0.050621 | 0.018608 | XBP1 | 1 |
| BP | GO:0070328 | triglyceride homeostasis | 1月10日 | 39/18866 | 0.020486 | 0.050621 | 0.018608 | XBP1 | 1 |
| BP | GO:2000279 | negative regulation of DNA biosynthetic process | 1月10日 | 39/18866 | 0.020486 | 0.050621 | 0.018608 | DUSP1 | 1 |
| BP | GO:2000816 | negative regulation of mitotic sister chromatid separation | 1月10日 | 39/18866 | 0.020486 | 0.050621 | 0.018608 | DUSP1 | 1 |
| BP | GO:0030099 | myeloid cell differentiation | 2月10日 | 431/18866 | 0.020754 | 0.051117 | 0.018791 | TNF,G6PD | 2 |
| BP | GO:0032102 | negative regulation of response to external stimulus | 2月10日 | 433/18866 | 0.020935 | 0.051117 | 0.018791 | TNF,DUSP1 | 2 |
| BP | GO:0000096 | sulfur amino acid metabolic process | 1月10日 | 40/18866 | 0.021006 | 0.051117 | 0.018791 | NOX4 | 1 |
| BP | GO:0002714 | positive regulation of B cell mediated immunity | 1月10日 | 40/18866 | 0.021006 | 0.051117 | 0.018791 | TNF | 1 |
| BP | GO:0002891 | positive regulation of immunoglobulin mediated immune response | 1月10日 | 40/18866 | 0.021006 | 0.051117 | 0.018791 | TNF | 1 |
| BP | GO:0016572 | histone phosphorylation | 1月10日 | 40/18866 | 0.021006 | 0.051117 | 0.018791 | MAPK3 | 1 |
| BP | GO:1902991 | regulation of amyloid precursor protein catabolic process | 1月10日 | 40/18866 | 0.021006 | 0.051117 | 0.018791 | TNF | 1 |
| BP | GO:1905819 | negative regulation of chromosome separation | 1月10日 | 40/18866 | 0.021006 | 0.051117 | 0.018791 | DUSP1 | 1 |
| BP | GO:0022407 | regulation of cell-cell adhesion | 2月10日 | 439/18866 | 0.021484 | 0.051987 | 0.01911 | TNF,XBP1 | 2 |
| BP | GO:0033574 | response to testosterone | 1月10日 | 41/18866 | 0.021526 | 0.051987 | 0.01911 | DUSP1 | 1 |
| BP | GO:0038083 | peptidyl-tyrosine autophosphorylation | 1月10日 | 41/18866 | 0.021526 | 0.051987 | 0.01911 | MAPK3 | 1 |
| BP | GO:2000008 | regulation of protein localization to cell surface | 1月10日 | 41/18866 | 0.021526 | 0.051987 | 0.01911 | TNF | 1 |
| BP | GO:0001933 | negative regulation of protein phosphorylation | 2月10日 | 444/18866 | 0.021945 | 0.052451 | 0.019281 | SIRT3,DUSP1 | 2 |
| BP | GO:1901342 | regulation of vasculature development | 2月10日 | 444/18866 | 0.021945 | 0.052451 | 0.019281 | TNF,XBP1 | 2 |
| BP | GO:0006509 | membrane protein ectodomain proteolysis | 1月10日 | 42/18866 | 0.022046 | 0.052451 | 0.019281 | TNF | 1 |
| BP | GO:0033048 | negative regulation of mitotic sister chromatid segregation | 1月10日 | 42/18866 | 0.022046 | 0.052451 | 0.019281 | DUSP1 | 1 |
| BP | GO:0046621 | negative regulation of organ growth | 1月10日 | 42/18866 | 0.022046 | 0.052451 | 0.019281 | G6PD | 1 |
| BP | GO:0048246 | macrophage chemotaxis | 1月10日 | 42/18866 | 0.022046 | 0.052451 | 0.019281 | MAPK3 | 1 |
| BP | GO:0090278 | negative regulation of peptide hormone secretion | 1月10日 | 42/18866 | 0.022046 | 0.052451 | 0.019281 | SREBF1 | 1 |
| BP | GO:0098927 | vesicle-mediated transport between endosomal compartments | 1月10日 | 42/18866 | 0.022046 | 0.052451 | 0.019281 | MAPK3 | 1 |
| BP | GO:0001819 | positive regulation of cytokine production | 2月10日 | 447/18866 | 0.022224 | 0.05268 | 0.019365 | TNF,XBP1 | 2 |
| BP | GO:0043434 | response to peptide hormone | 2月10日 | 447/18866 | 0.022224 | 0.05268 | 0.019365 | SREBF1,XBP1 | 2 |
| BP | GO:0006417 | regulation of translation | 2月10日 | 450/18866 | 0.022505 | 0.052765 | 0.019396 | TNF,MAPK3 | 2 |
| BP | GO:0019432 | triglyceride biosynthetic process | 1月10日 | 43/18866 | 0.022565 | 0.052765 | 0.019396 | SREBF1 | 1 |
| BP | GO:0021762 | substantia nigra development | 1月10日 | 43/18866 | 0.022565 | 0.052765 | 0.019396 | G6PD | 1 |
| BP | GO:0050850 | positive regulation of calcium-mediated signaling | 1月10日 | 43/18866 | 0.022565 | 0.052765 | 0.019396 | TNF | 1 |
| BP | GO:0090207 | regulation of triglyceride metabolic process | 1月10日 | 43/18866 | 0.022565 | 0.052765 | 0.019396 | SREBF1 | 1 |
| BP | GO:0150077 | regulation of neuroinflammatory response | 1月10日 | 43/18866 | 0.022565 | 0.052765 | 0.019396 | TNF | 1 |
| BP | GO:1903146 | regulation of autophagy of mitochondrion | 1月10日 | 43/18866 | 0.022565 | 0.052765 | 0.019396 | SREBF1 | 1 |
| BP | GO:0048568 | embryonic organ development | 2月10日 | 451/18866 | 0.022599 | 0.052765 | 0.019396 | TNF,MAPK3 | 2 |
| BP | GO:0050673 | epithelial cell proliferation | 2月10日 | 453/18866 | 0.022787 | 0.052765 | 0.019396 | TNF,XBP1 | 2 |
| BP | GO:0009896 | positive regulation of catabolic process | 2月10日 | 454/18866 | 0.022881 | 0.052765 | 0.019396 | TNF,MAPK3 | 2 |
| BP | GO:0050804 | modulation of chemical synaptic transmission | 2月10日 | 454/18866 | 0.022881 | 0.052765 | 0.019396 | TNF,KRAS | 2 |
| BP | GO:0099177 | regulation of trans-synaptic signaling | 2月10日 | 455/18866 | 0.022976 | 0.052765 | 0.019396 | TNF,KRAS | 2 |
| BP | GO:0062012 | regulation of small molecule metabolic process | 2月10日 | 456/18866 | 0.02307 | 0.052765 | 0.019396 | TNF,SREBF1 | 2 |
| BP | GO:0031670 | cellular response to nutrient | 1月10日 | 44/18866 | 0.023085 | 0.052765 | 0.019396 | XBP1 | 1 |
| BP | GO:0033046 | negative regulation of sister chromatid segregation | 1月10日 | 44/18866 | 0.023085 | 0.052765 | 0.019396 | DUSP1 | 1 |
| BP | GO:0033173 | calcineurin-NFAT signaling cascade | 1月10日 | 44/18866 | 0.023085 | 0.052765 | 0.019396 | TNF | 1 |
| BP | GO:0045684 | positive regulation of epidermis development | 1月10日 | 44/18866 | 0.023085 | 0.052765 | 0.019396 | TNF | 1 |
| BP | GO:0060612 | adipose tissue development | 1月10日 | 44/18866 | 0.023085 | 0.052765 | 0.019396 | XBP1 | 1 |
| BP | GO:1901985 | positive regulation of protein acetylation | 1月10日 | 44/18866 | 0.023085 | 0.052765 | 0.019396 | MAPK3 | 1 |
| BP | GO:1905521 | regulation of macrophage migration | 1月10日 | 44/18866 | 0.023085 | 0.052765 | 0.019396 | MAPK3 | 1 |
| BP | GO:0002639 | positive regulation of immunoglobulin production | 1月10日 | 45/18866 | 0.023604 | 0.052912 | 0.01945 | XBP1 | 1 |
| BP | GO:0006775 | fat-soluble vitamin metabolic process | 1月10日 | 45/18866 | 0.023604 | 0.052912 | 0.01945 | TNF | 1 |
| BP | GO:0032722 | positive regulation of chemokine production | 1月10日 | 45/18866 | 0.023604 | 0.052912 | 0.01945 | TNF | 1 |
| BP | GO:0034198 | cellular response to amino acid starvation | 1月10日 | 45/18866 | 0.023604 | 0.052912 | 0.01945 | MAPK3 | 1 |
| BP | GO:0035094 | response to nicotine | 1月10日 | 45/18866 | 0.023604 | 0.052912 | 0.01945 | TNF | 1 |
| BP | GO:0045429 | positive regulation of nitric oxide biosynthetic process | 1月10日 | 45/18866 | 0.023604 | 0.052912 | 0.01945 | TNF | 1 |
| BP | GO:0051985 | negative regulation of chromosome segregation | 1月10日 | 45/18866 | 0.023604 | 0.052912 | 0.01945 | DUSP1 | 1 |
| BP | GO:0055026 | negative regulation of cardiac muscle tissue development | 1月10日 | 45/18866 | 0.023604 | 0.052912 | 0.01945 | G6PD | 1 |
| BP | GO:0070266 | necroptotic process | 1月10日 | 45/18866 | 0.023604 | 0.052912 | 0.01945 | TNF | 1 |
| BP | GO:0090311 | regulation of protein deacetylation | 1月10日 | 45/18866 | 0.023604 | 0.052912 | 0.01945 | SREBF1 | 1 |
| BP | GO:1903214 | regulation of protein targeting to mitochondrion | 1月10日 | 45/18866 | 0.023604 | 0.052912 | 0.01945 | SREBF1 | 1 |
| BP | GO:0002683 | negative regulation of immune system process | 2月10日 | 463/18866 | 0.023738 | 0.05312 | 0.019527 | TNF,DUSP1 | 2 |
| BP | GO:0052547 | regulation of peptidase activity | 2月10日 | 466/18866 | 0.024026 | 0.053328 | 0.019603 | TNF,MAPK3 | 2 |
| BP | GO:0032570 | response to progesterone | 1月10日 | 46/18866 | 0.024122 | 0.053328 | 0.019603 | SREBF1 | 1 |
| BP | GO:0043330 | response to exogenous dsRNA | 1月10日 | 46/18866 | 0.024122 | 0.053328 | 0.019603 | MAPK3 | 1 |
| BP | GO:0048538 | thymus development | 1月10日 | 46/18866 | 0.024122 | 0.053328 | 0.019603 | MAPK3 | 1 |
| BP | GO:0061028 | establishment of endothelial barrier | 1月10日 | 46/18866 | 0.024122 | 0.053328 | 0.019603 | TNF | 1 |
| BP | GO:1900744 | regulation of p38MAPK cascade | 1月10日 | 46/18866 | 0.024122 | 0.053328 | 0.019603 | DUSP1 | 1 |
| BP | GO:1904407 | positive regulation of nitric oxide metabolic process | 1月10日 | 46/18866 | 0.024122 | 0.053328 | 0.019603 | TNF | 1 |
| BP | GO:0002697 | regulation of immune effector process | 2月10日 | 470/18866 | 0.024413 | 0.053878 | 0.019805 | TNF,XBP1 | 2 |
| BP | GO:0006953 | acute-phase response | 1月10日 | 47/18866 | 0.024641 | 0.0541 | 0.019887 | TNF | 1 |
| BP | GO:0008542 | visual learning | 1月10日 | 47/18866 | 0.024641 | 0.0541 | 0.019887 | KRAS | 1 |
| BP | GO:0070849 | response to epidermal growth factor | 1月10日 | 47/18866 | 0.024641 | 0.0541 | 0.019887 | MAPK3 | 1 |
| BP | GO:0014911 | positive regulation of smooth muscle cell migration | 1月10日 | 48/18866 | 0.025159 | 0.054583 | 0.020065 | NOX4 | 1 |
| BP | GO:0045601 | regulation of endothelial cell differentiation | 1月10日 | 48/18866 | 0.025159 | 0.054583 | 0.020065 | TNF | 1 |
| BP | GO:0051932 | synaptic transmission, GABAergic | 1月10日 | 48/18866 | 0.025159 | 0.054583 | 0.020065 | KRAS | 1 |
| BP | GO:0051972 | regulation of telomerase activity | 1月10日 | 48/18866 | 0.025159 | 0.054583 | 0.020065 | MAPK3 | 1 |
| BP | GO:0060324 | face development | 1月10日 | 48/18866 | 0.025159 | 0.054583 | 0.020065 | MAPK3 | 1 |
| BP | GO:1990928 | response to amino acid starvation | 1月10日 | 48/18866 | 0.025159 | 0.054583 | 0.020065 | MAPK3 | 1 |
| BP | GO:2001239 | regulation of extrinsic apoptotic signaling pathway in absence of ligand | 1月10日 | 48/18866 | 0.025159 | 0.054583 | 0.020065 | TNF | 1 |
| BP | GO:0002429 | immune response-activating cell surface receptor signaling pathway | 2月10日 | 481/18866 | 0.025491 | 0.054804 | 0.020146 | MAPK3,KRAS | 2 |
| BP | GO:0002757 | immune response-activating signal transduction | 2月10日 | 481/18866 | 0.025491 | 0.054804 | 0.020146 | MAPK3,KRAS | 2 |
| BP | GO:0002673 | regulation of acute inflammatory response | 1月10日 | 49/18866 | 0.025677 | 0.054804 | 0.020146 | TNF | 1 |
| BP | GO:0002686 | negative regulation of leukocyte migration | 1月10日 | 49/18866 | 0.025677 | 0.054804 | 0.020146 | DUSP1 | 1 |
| BP | GO:0007595 | lactation | 1月10日 | 49/18866 | 0.025677 | 0.054804 | 0.020146 | XBP1 | 1 |
| BP | GO:0031279 | regulation of cyclase activity | 1月10日 | 49/18866 | 0.025677 | 0.054804 | 0.020146 | MAPK3 | 1 |
| BP | GO:0035196 | production of miRNAs involved in gene silencing by miRNA | 1月10日 | 49/18866 | 0.025677 | 0.054804 | 0.020146 | TNF | 1 |
| BP | GO:0042149 | cellular response to glucose starvation | 1月10日 | 49/18866 | 0.025677 | 0.054804 | 0.020146 | XBP1 | 1 |
| BP | GO:0097720 | calcineurin-mediated signaling | 1月10日 | 49/18866 | 0.025677 | 0.054804 | 0.020146 | TNF | 1 |
| BP | GO:0051051 | negative regulation of transport | 2月10日 | 483/18866 | 0.025689 | 0.054804 | 0.020146 | TNF,SREBF1 | 2 |
| BP | GO:0042326 | negative regulation of phosphorylation | 2月10日 | 484/18866 | 0.025789 | 0.054877 | 0.020173 | SIRT3,DUSP1 | 2 |
| BP | GO:0001774 | microglial cell activation | 1月10日 | 50/18866 | 0.026195 | 0.054877 | 0.020173 | TNF | 1 |
| BP | GO:0002269 | leukocyte activation involved in inflammatory response | 1月10日 | 50/18866 | 0.026195 | 0.054877 | 0.020173 | TNF | 1 |
| BP | GO:0014009 | glial cell proliferation | 1月10日 | 50/18866 | 0.026195 | 0.054877 | 0.020173 | TNF | 1 |
| BP | GO:0031648 | protein destabilization | 1月10日 | 50/18866 | 0.026195 | 0.054877 | 0.020173 | XBP1 | 1 |
| BP | GO:0042987 | amyloid precursor protein catabolic process | 1月10日 | 50/18866 | 0.026195 | 0.054877 | 0.020173 | TNF | 1 |
| BP | GO:0045540 | regulation of cholesterol biosynthetic process | 1月10日 | 50/18866 | 0.026195 | 0.054877 | 0.020173 | SREBF1 | 1 |
| BP | GO:0097300 | programmed necrotic cell death | 1月10日 | 50/18866 | 0.026195 | 0.054877 | 0.020173 | TNF | 1 |
| BP | GO:0106118 | regulation of sterol biosynthetic process | 1月10日 | 50/18866 | 0.026195 | 0.054877 | 0.020173 | SREBF1 | 1 |
| BP | GO:1904707 | positive regulation of vascular associated smooth muscle cell proliferation | 1月10日 | 50/18866 | 0.026195 | 0.054877 | 0.020173 | TNF | 1 |
| BP | GO:2000725 | regulation of cardiac muscle cell differentiation | 1月10日 | 50/18866 | 0.026195 | 0.054877 | 0.020173 | G6PD | 1 |
| BP | GO:0016311 | dephosphorylation | 2月10日 | 492/18866 | 0.026589 | 0.055238 | 0.020305 | TNF,DUSP1 | 2 |
| BP | GO:0032206 | positive regulation of telomere maintenance | 1月10日 | 51/18866 | 0.026713 | 0.055238 | 0.020305 | MAPK3 | 1 |
| BP | GO:0032873 | negative regulation of stress-activated MAPK cascade | 1月10日 | 51/18866 | 0.026713 | 0.055238 | 0.020305 | DUSP1 | 1 |
| BP | GO:0046460 | neutral lipid biosynthetic process | 1月10日 | 51/18866 | 0.026713 | 0.055238 | 0.020305 | SREBF1 | 1 |
| BP | GO:0046463 | acylglycerol biosynthetic process | 1月10日 | 51/18866 | 0.026713 | 0.055238 | 0.020305 | SREBF1 | 1 |
| BP | GO:0050435 | amyloid-beta metabolic process | 1月10日 | 51/18866 | 0.026713 | 0.055238 | 0.020305 | TNF | 1 |
| BP | GO:0070303 | negative regulation of stress-activated protein kinase signaling cascade | 1月10日 | 51/18866 | 0.026713 | 0.055238 | 0.020305 | DUSP1 | 1 |
| BP | GO:1903573 | negative regulation of response to endoplasmic reticulum stress | 1月10日 | 51/18866 | 0.026713 | 0.055238 | 0.020305 | XBP1 | 1 |
| BP | GO:1903706 | regulation of hemopoiesis | 2月10日 | 498/18866 | 0.027196 | 0.0555 | 0.020402 | TNF,XBP1 | 2 |
| BP | GO:0000186 | activation of MAPKK activity | 1月10日 | 52/18866 | 0.02723 | 0.0555 | 0.020402 | MAPK3 | 1 |
| BP | GO:0010883 | regulation of lipid storage | 1月10日 | 52/18866 | 0.02723 | 0.0555 | 0.020402 | TNF | 1 |
| BP | GO:0038066 | p38MAPK cascade | 1月10日 | 52/18866 | 0.02723 | 0.0555 | 0.020402 | DUSP1 | 1 |
| BP | GO:0045839 | negative regulation of mitotic nuclear division | 1月10日 | 52/18866 | 0.02723 | 0.0555 | 0.020402 | DUSP1 | 1 |
| BP | GO:0051445 | regulation of meiotic cell cycle | 1月10日 | 52/18866 | 0.02723 | 0.0555 | 0.020402 | DUSP1 | 1 |
| BP | GO:0051496 | positive regulation of stress fiber assembly | 1月10日 | 52/18866 | 0.02723 | 0.0555 | 0.020402 | NOX4 | 1 |
| BP | GO:0071622 | regulation of granulocyte chemotaxis | 1月10日 | 52/18866 | 0.02723 | 0.0555 | 0.020402 | MAPK3 | 1 |
| BP | GO:2001238 | positive regulation of extrinsic apoptotic signaling pathway | 1月10日 | 52/18866 | 0.02723 | 0.0555 | 0.020402 | TNF | 1 |
| BP | GO:0007632 | visual behavior | 1月10日 | 53/18866 | 0.027747 | 0.055581 | 0.020431 | KRAS | 1 |
| BP | GO:0032210 | regulation of telomere maintenance via telomerase | 1月10日 | 53/18866 | 0.027747 | 0.055581 | 0.020431 | MAPK3 | 1 |
| BP | GO:0032731 | positive regulation of interleukin-1 beta production | 1月10日 | 53/18866 | 0.027747 | 0.055581 | 0.020431 | TNF | 1 |
| BP | GO:0043331 | response to dsRNA | 1月10日 | 53/18866 | 0.027747 | 0.055581 | 0.020431 | MAPK3 | 1 |
| BP | GO:0045599 | negative regulation of fat cell differentiation | 1月10日 | 53/18866 | 0.027747 | 0.055581 | 0.020431 | TNF | 1 |
| BP | GO:0045661 | regulation of myoblast differentiation | 1月10日 | 53/18866 | 0.027747 | 0.055581 | 0.020431 | TNF | 1 |
| BP | GO:0045668 | negative regulation of osteoblast differentiation | 1月10日 | 53/18866 | 0.027747 | 0.055581 | 0.020431 | TNF | 1 |
| BP | GO:0048168 | regulation of neuronal synaptic plasticity | 1月10日 | 53/18866 | 0.027747 | 0.055581 | 0.020431 | KRAS | 1 |
| BP | GO:0061756 | leukocyte adhesion to vascular endothelial cell | 1月10日 | 53/18866 | 0.027747 | 0.055581 | 0.020431 | TNF | 1 |
| BP | GO:0071320 | cellular response to cAMP | 1月10日 | 53/18866 | 0.027747 | 0.055581 | 0.020431 | NOX4 | 1 |
| BP | GO:2000772 | regulation of cellular senescence | 1月10日 | 53/18866 | 0.027747 | 0.055581 | 0.020431 | KRAS | 1 |
| BP | GO:0006749 | glutathione metabolic process | 1月10日 | 54/18866 | 0.028264 | 0.056264 | 0.020682 | G6PD | 1 |
| BP | GO:0031050 | dsRNA processing | 1月10日 | 54/18866 | 0.028264 | 0.056264 | 0.020682 | TNF | 1 |
| BP | GO:0032757 | positive regulation of interleukin-8 production | 1月10日 | 54/18866 | 0.028264 | 0.056264 | 0.020682 | TNF | 1 |
| BP | GO:0070918 | production of small RNA involved in gene silencing by RNA | 1月10日 | 54/18866 | 0.028264 | 0.056264 | 0.020682 | TNF | 1 |
| BP | GO:0001706 | endoderm formation | 1月10日 | 55/18866 | 0.02878 | 0.057026 | 0.020963 | DUSP1 | 1 |
| BP | GO:0016233 | telomere capping | 1月10日 | 55/18866 | 0.02878 | 0.057026 | 0.020963 | MAPK3 | 1 |
| BP | GO:0035065 | regulation of histone acetylation | 1月10日 | 55/18866 | 0.02878 | 0.057026 | 0.020963 | MAPK3 | 1 |
| BP | GO:0002712 | regulation of B cell mediated immunity | 1月10日 | 56/18866 | 0.029297 | 0.057603 | 0.021175 | TNF | 1 |
| BP | GO:0002889 | regulation of immunoglobulin mediated immune response | 1月10日 | 56/18866 | 0.029297 | 0.057603 | 0.021175 | TNF | 1 |
| BP | GO:0010332 | response to gamma radiation | 1月10日 | 56/18866 | 0.029297 | 0.057603 | 0.021175 | NOX4 | 1 |
| BP | GO:0030071 | regulation of mitotic metaphase/anaphase transition | 1月10日 | 56/18866 | 0.029297 | 0.057603 | 0.021175 | DUSP1 | 1 |
| BP | GO:0060688 | regulation of morphogenesis of a branching structure | 1月10日 | 56/18866 | 0.029297 | 0.057603 | 0.021175 | TNF | 1 |
| BP | GO:0048016 | inositol phosphate-mediated signaling | 1月10日 | 57/18866 | 0.029813 | 0.058349 | 0.021449 | TNF | 1 |
| BP | GO:0071398 | cellular response to fatty acid | 1月10日 | 57/18866 | 0.029813 | 0.058349 | 0.021449 | SREBF1 | 1 |
| BP | GO:1905517 | macrophage migration | 1月10日 | 57/18866 | 0.029813 | 0.058349 | 0.021449 | MAPK3 | 1 |
| BP | GO:0002763 | positive regulation of myeloid leukocyte differentiation | 1月10日 | 58/18866 | 0.030328 | 0.058998 | 0.021687 | TNF | 1 |
| BP | GO:0007091 | metaphase/anaphase transition of mitotic cell cycle | 1月10日 | 58/18866 | 0.030328 | 0.058998 | 0.021687 | DUSP1 | 1 |
| BP | GO:0019369 | arachidonic acid metabolic process | 1月10日 | 58/18866 | 0.030328 | 0.058998 | 0.021687 | MAPK3 | 1 |
| BP | GO:1902099 | regulation of metaphase/anaphase transition of cell cycle | 1月10日 | 58/18866 | 0.030328 | 0.058998 | 0.021687 | DUSP1 | 1 |
| BP | GO:0043525 | positive regulation of neuron apoptotic process | 1月10日 | 59/18866 | 0.030844 | 0.059819 | 0.021989 | TNF | 1 |
| BP | GO:0050707 | regulation of cytokine secretion | 1月10日 | 59/18866 | 0.030844 | 0.059819 | 0.021989 | TNF | 1 |
| BP | GO:0030865 | cortical cytoskeleton organization | 1月10日 | 60/18866 | 0.031359 | 0.059823 | 0.021991 | TNF | 1 |
| BP | GO:0032515 | negative regulation of phosphoprotein phosphatase activity | 1月10日 | 60/18866 | 0.031359 | 0.059823 | 0.021991 | TNF | 1 |
| BP | GO:0032732 | positive regulation of interleukin-1 production | 1月10日 | 60/18866 | 0.031359 | 0.059823 | 0.021991 | TNF | 1 |
| BP | GO:0044784 | metaphase/anaphase transition of cell cycle | 1月10日 | 60/18866 | 0.031359 | 0.059823 | 0.021991 | DUSP1 | 1 |
| BP | GO:0050994 | regulation of lipid catabolic process | 1月10日 | 60/18866 | 0.031359 | 0.059823 | 0.021991 | TNF | 1 |
| BP | GO:0051784 | negative regulation of nuclear division | 1月10日 | 60/18866 | 0.031359 | 0.059823 | 0.021991 | DUSP1 | 1 |
| BP | GO:0061098 | positive regulation of protein tyrosine kinase activity | 1月10日 | 60/18866 | 0.031359 | 0.059823 | 0.021991 | NOX4 | 1 |
| BP | GO:0061900 | glial cell activation | 1月10日 | 60/18866 | 0.031359 | 0.059823 | 0.021991 | TNF | 1 |
| BP | GO:1903428 | positive regulation of reactive oxygen species biosynthetic process | 1月10日 | 60/18866 | 0.031359 | 0.059823 | 0.021991 | TNF | 1 |
| BP | GO:2000242 | negative regulation of reproductive process | 1月10日 | 60/18866 | 0.031359 | 0.059823 | 0.021991 | DUSP1 | 1 |
| BP | GO:0010965 | regulation of mitotic sister chromatid separation | 1月10日 | 61/18866 | 0.031874 | 0.059823 | 0.021991 | DUSP1 | 1 |
| BP | GO:0033619 | membrane protein proteolysis | 1月10日 | 61/18866 | 0.031874 | 0.059823 | 0.021991 | TNF | 1 |
| BP | GO:0045071 | negative regulation of viral genome replication | 1月10日 | 61/18866 | 0.031874 | 0.059823 | 0.021991 | TNF | 1 |
| BP | GO:0045843 | negative regulation of striated muscle tissue development | 1月10日 | 61/18866 | 0.031874 | 0.059823 | 0.021991 | G6PD | 1 |
| BP | GO:0046324 | regulation of glucose import | 1月10日 | 61/18866 | 0.031874 | 0.059823 | 0.021991 | TNF | 1 |
| BP | GO:0046888 | negative regulation of hormone secretion | 1月10日 | 61/18866 | 0.031874 | 0.059823 | 0.021991 | SREBF1 | 1 |
| BP | GO:0061912 | selective autophagy | 1月10日 | 61/18866 | 0.031874 | 0.059823 | 0.021991 | MAPK3 | 1 |
| BP | GO:0090303 | positive regulation of wound healing | 1月10日 | 61/18866 | 0.031874 | 0.059823 | 0.021991 | XBP1 | 1 |
| BP | GO:0090342 | regulation of cell aging | 1月10日 | 61/18866 | 0.031874 | 0.059823 | 0.021991 | KRAS | 1 |
| BP | GO:1903078 | positive regulation of protein localization to plasma membrane | 1月10日 | 61/18866 | 0.031874 | 0.059823 | 0.021991 | TNF | 1 |
| BP | GO:1904356 | regulation of telomere maintenance via telomere lengthening | 1月10日 | 61/18866 | 0.031874 | 0.059823 | 0.021991 | MAPK3 | 1 |
| BP | GO:2000756 | regulation of peptidyl-lysine acetylation | 1月10日 | 61/18866 | 0.031874 | 0.059823 | 0.021991 | MAPK3 | 1 |
| BP | GO:0010803 | regulation of tumor necrosis factor-mediated signaling pathway | 1月10日 | 62/18866 | 0.032389 | 0.060172 | 0.022119 | TNF | 1 |
| BP | GO:0032233 | positive regulation of actin filament bundle assembly | 1月10日 | 62/18866 | 0.032389 | 0.060172 | 0.022119 | NOX4 | 1 |
| BP | GO:0034113 | heterotypic cell-cell adhesion | 1月10日 | 62/18866 | 0.032389 | 0.060172 | 0.022119 | TNF | 1 |
| BP | GO:0048635 | negative regulation of muscle organ development | 1月10日 | 62/18866 | 0.032389 | 0.060172 | 0.022119 | G6PD | 1 |
| BP | GO:0048857 | neural nucleus development | 1月10日 | 62/18866 | 0.032389 | 0.060172 | 0.022119 | G6PD | 1 |
| BP | GO:0055081 | anion homeostasis | 1月10日 | 62/18866 | 0.032389 | 0.060172 | 0.022119 | XBP1 | 1 |
| BP | GO:2000351 | regulation of endothelial cell apoptotic process | 1月10日 | 62/18866 | 0.032389 | 0.060172 | 0.022119 | TNF | 1 |
| BP | GO:0010830 | regulation of myotube differentiation | 1月10日 | 63/18866 | 0.032904 | 0.060951 | 0.022405 | XBP1 | 1 |
| BP | GO:0070059 | intrinsic apoptotic signaling pathway in response to endoplasmic reticulum stress | 1月10日 | 63/18866 | 0.032904 | 0.060951 | 0.022405 | XBP1 | 1 |
| BP | GO:0001885 | endothelial cell development | 1月10日 | 64/18866 | 0.033418 | 0.061195 | 0.022495 | TNF | 1 |
| BP | GO:0002437 | inflammatory response to antigenic stimulus | 1月10日 | 64/18866 | 0.033418 | 0.061195 | 0.022495 | TNF | 1 |
| BP | GO:0045453 | bone resorption | 1月10日 | 64/18866 | 0.033418 | 0.061195 | 0.022495 | NOX4 | 1 |
| BP | GO:0046686 | response to cadmium ion | 1月10日 | 64/18866 | 0.033418 | 0.061195 | 0.022495 | MAPK3 | 1 |
| BP | GO:0051306 | mitotic sister chromatid separation | 1月10日 | 64/18866 | 0.033418 | 0.061195 | 0.022495 | DUSP1 | 1 |
| BP | GO:0060135 | maternal process involved in female pregnancy | 1月10日 | 64/18866 | 0.033418 | 0.061195 | 0.022495 | MAPK3 | 1 |
| BP | GO:0070265 | necrotic cell death | 1月10日 | 64/18866 | 0.033418 | 0.061195 | 0.022495 | TNF | 1 |
| BP | GO:1901862 | negative regulation of muscle tissue development | 1月10日 | 64/18866 | 0.033418 | 0.061195 | 0.022495 | G6PD | 1 |
| BP | GO:0010573 | vascular endothelial growth factor production | 1月10日 | 65/18866 | 0.033932 | 0.061607 | 0.022647 | TNF | 1 |
| BP | GO:0032729 | positive regulation of interferon-gamma production | 1月10日 | 65/18866 | 0.033932 | 0.061607 | 0.022647 | TNF | 1 |
| BP | GO:0035308 | negative regulation of protein dephosphorylation | 1月10日 | 65/18866 | 0.033932 | 0.061607 | 0.022647 | TNF | 1 |
| BP | GO:0048645 | animal organ formation | 1月10日 | 65/18866 | 0.033932 | 0.061607 | 0.022647 | MAPK3 | 1 |
| BP | GO:0090181 | regulation of cholesterol metabolic process | 1月10日 | 65/18866 | 0.033932 | 0.061607 | 0.022647 | SREBF1 | 1 |
| BP | GO:1905953 | negative regulation of lipid localization | 1月10日 | 65/18866 | 0.033932 | 0.061607 | 0.022647 | TNF | 1 |
| BP | GO:0006360 | transcription by RNA polymerase I | 1月10日 | 66/18866 | 0.034446 | 0.062013 | 0.022796 | MAPK3 | 1 |
| BP | GO:0032715 | negative regulation of interleukin-6 production | 1月10日 | 66/18866 | 0.034446 | 0.062013 | 0.022796 | TNF | 1 |
| BP | GO:0034394 | protein localization to cell surface | 1月10日 | 66/18866 | 0.034446 | 0.062013 | 0.022796 | TNF | 1 |
| BP | GO:0050922 | negative regulation of chemotaxis | 1月10日 | 66/18866 | 0.034446 | 0.062013 | 0.022796 | DUSP1 | 1 |
| BP | GO:1905207 | regulation of cardiocyte differentiation | 1月10日 | 66/18866 | 0.034446 | 0.062013 | 0.022796 | G6PD | 1 |
| BP | GO:1905818 | regulation of chromosome separation | 1月10日 | 66/18866 | 0.034446 | 0.062013 | 0.022796 | DUSP1 | 1 |
| BP | GO:0002548 | monocyte chemotaxis | 1月10日 | 67/18866 | 0.03496 | 0.062498 | 0.022974 | DUSP1 | 1 |
| BP | GO:0036498 | IRE1-mediated unfolded protein response | 1月10日 | 67/18866 | 0.03496 | 0.062498 | 0.022974 | XBP1 | 1 |
| BP | GO:0045123 | cellular extravasation | 1月10日 | 67/18866 | 0.03496 | 0.062498 | 0.022974 | TNF | 1 |
| BP | GO:0045670 | regulation of osteoclast differentiation | 1月10日 | 67/18866 | 0.03496 | 0.062498 | 0.022974 | TNF | 1 |
| BP | GO:1900015 | regulation of cytokine production involved in inflammatory response | 1月10日 | 67/18866 | 0.03496 | 0.062498 | 0.022974 | TNF | 1 |
| BP | GO:0016239 | positive regulation of macroautophagy | 1月10日 | 68/18866 | 0.035473 | 0.062801 | 0.023086 | MAPK3 | 1 |
| BP | GO:0032642 | regulation of chemokine production | 1月10日 | 68/18866 | 0.035473 | 0.062801 | 0.023086 | TNF | 1 |
| BP | GO:0045428 | regulation of nitric oxide biosynthetic process | 1月10日 | 68/18866 | 0.035473 | 0.062801 | 0.023086 | TNF | 1 |
| BP | GO:0050766 | positive regulation of phagocytosis | 1月10日 | 68/18866 | 0.035473 | 0.062801 | 0.023086 | TNF | 1 |
| BP | GO:0071479 | cellular response to ionizing radiation | 1月10日 | 68/18866 | 0.035473 | 0.062801 | 0.023086 | NOX4 | 1 |
| BP | GO:0072577 | endothelial cell apoptotic process | 1月10日 | 68/18866 | 0.035473 | 0.062801 | 0.023086 | TNF | 1 |
| BP | GO:1904377 | positive regulation of protein localization to cell periphery | 1月10日 | 68/18866 | 0.035473 | 0.062801 | 0.023086 | TNF | 1 |
| BP | GO:0007004 | telomere maintenance via telomerase | 1月10日 | 69/18866 | 0.035986 | 0.063622 | 0.023387 | MAPK3 | 1 |
| BP | GO:0002534 | cytokine production involved in inflammatory response | 1月10日 | 70/18866 | 0.036499 | 0.064439 | 0.023688 | TNF | 1 |
| BP | GO:0042531 | positive regulation of tyrosine phosphorylation of STAT protein | 1月10日 | 71/18866 | 0.037012 | 0.065075 | 0.023922 | TNF | 1 |
| BP | GO:0042982 | amyloid precursor protein metabolic process | 1月10日 | 71/18866 | 0.037012 | 0.065075 | 0.023922 | TNF | 1 |
| BP | GO:0046579 | positive regulation of Ras protein signal transduction | 1月10日 | 71/18866 | 0.037012 | 0.065075 | 0.023922 | KRAS | 1 |
| BP | GO:0033047 | regulation of mitotic sister chromatid segregation | 1月10日 | 72/18866 | 0.037524 | 0.065526 | 0.024087 | DUSP1 | 1 |
| BP | GO:0045600 | positive regulation of fat cell differentiation | 1月10日 | 72/18866 | 0.037524 | 0.065526 | 0.024087 | XBP1 | 1 |
| BP | GO:0050663 | cytokine secretion | 1月10日 | 72/18866 | 0.037524 | 0.065526 | 0.024087 | TNF | 1 |
| BP | GO:0061515 | myeloid cell development | 1月10日 | 72/18866 | 0.037524 | 0.065526 | 0.024087 | G6PD | 1 |
| BP | GO:1904427 | positive regulation of calcium ion transmembrane transport | 1月10日 | 72/18866 | 0.037524 | 0.065526 | 0.024087 | G6PD | 1 |
| BP | GO:0010611 | regulation of cardiac muscle hypertrophy | 1月10日 | 73/18866 | 0.038036 | 0.06606 | 0.024284 | G6PD | 1 |
| BP | GO:0051966 | regulation of synaptic transmission, glutamatergic | 1月10日 | 73/18866 | 0.038036 | 0.06606 | 0.024284 | TNF | 1 |
| BP | GO:1902117 | positive regulation of organelle assembly | 1月10日 | 73/18866 | 0.038036 | 0.06606 | 0.024284 | TNF | 1 |
| BP | GO:1903747 | regulation of establishment of protein localization to mitochondrion | 1月10日 | 73/18866 | 0.038036 | 0.06606 | 0.024284 | SREBF1 | 1 |
| BP | GO:0032024 | positive regulation of insulin secretion | 1月10日 | 74/18866 | 0.038548 | 0.066677 | 0.024511 | SIRT3 | 1 |
| BP | GO:0043507 | positive regulation of JUN kinase activity | 1月10日 | 74/18866 | 0.038548 | 0.066677 | 0.024511 | TNF | 1 |
| BP | GO:1903036 | positive regulation of response to wounding | 1月10日 | 74/18866 | 0.038548 | 0.066677 | 0.024511 | XBP1 | 1 |
| BP | GO:0032602 | chemokine production | 1月10日 | 75/18866 | 0.03906 | 0.067109 | 0.024669 | TNF | 1 |
| BP | GO:0038034 | signal transduction in absence of ligand | 1月10日 | 75/18866 | 0.03906 | 0.067109 | 0.024669 | TNF | 1 |
| BP | GO:0046323 | glucose import | 1月10日 | 75/18866 | 0.03906 | 0.067109 | 0.024669 | TNF | 1 |
| BP | GO:0097192 | extrinsic apoptotic signaling pathway in absence of ligand | 1月10日 | 75/18866 | 0.03906 | 0.067109 | 0.024669 | TNF | 1 |
| BP | GO:1901983 | regulation of protein acetylation | 1月10日 | 75/18866 | 0.03906 | 0.067109 | 0.024669 | MAPK3 | 1 |
| BP | GO:0006278 | RNA-dependent DNA biosynthetic process | 1月10日 | 76/18866 | 0.039571 | 0.067715 | 0.024892 | MAPK3 | 1 |
| BP | GO:0008306 | associative learning | 1月10日 | 76/18866 | 0.039571 | 0.067715 | 0.024892 | KRAS | 1 |
| BP | GO:0014743 | regulation of muscle hypertrophy | 1月10日 | 76/18866 | 0.039571 | 0.067715 | 0.024892 | G6PD | 1 |
| BP | GO:0014015 | positive regulation of gliogenesis | 1月10日 | 77/18866 | 0.040082 | 0.068316 | 0.025113 | TNF | 1 |
| BP | GO:0070830 | bicellular tight junction assembly | 1月10日 | 77/18866 | 0.040082 | 0.068316 | 0.025113 | TNF | 1 |
| BP | GO:0150076 | neuroinflammatory response | 1月10日 | 77/18866 | 0.040082 | 0.068316 | 0.025113 | TNF | 1 |
| BP | GO:0051057 | positive regulation of small GTPase mediated signal transduction | 1月10日 | 78/18866 | 0.040593 | 0.068775 | 0.025281 | KRAS | 1 |
| BP | GO:0061045 | negative regulation of wound healing | 1月10日 | 78/18866 | 0.040593 | 0.068775 | 0.025281 | TNF | 1 |
| BP | GO:0000422 | autophagy of mitochondrion | 1月10日 | 79/18866 | 0.041104 | 0.068775 | 0.025281 | SREBF1 | 1 |
| BP | GO:0006446 | regulation of translational initiation | 1月10日 | 79/18866 | 0.041104 | 0.068775 | 0.025281 | TNF | 1 |
| BP | GO:0006809 | nitric oxide biosynthetic process | 1月10日 | 79/18866 | 0.041104 | 0.068775 | 0.025281 | TNF | 1 |
| BP | GO:0007492 | endoderm development | 1月10日 | 79/18866 | 0.041104 | 0.068775 | 0.025281 | DUSP1 | 1 |
| BP | GO:0010827 | regulation of glucose transmembrane transport | 1月10日 | 79/18866 | 0.041104 | 0.068775 | 0.025281 | TNF | 1 |
| BP | GO:0031016 | pancreas development | 1月10日 | 79/18866 | 0.041104 | 0.068775 | 0.025281 | XBP1 | 1 |
| BP | GO:0043407 | negative regulation of MAP kinase activity | 1月10日 | 79/18866 | 0.041104 | 0.068775 | 0.025281 | DUSP1 | 1 |
| BP | GO:0061726 | mitochondrion disassembly | 1月10日 | 79/18866 | 0.041104 | 0.068775 | 0.025281 | SREBF1 | 1 |
| BP | GO:0071260 | cellular response to mechanical stimulus | 1月10日 | 79/18866 | 0.041104 | 0.068775 | 0.025281 | MAPK3 | 1 |
| BP | GO:0090398 | cellular senescence | 1月10日 | 79/18866 | 0.041104 | 0.068775 | 0.025281 | KRAS | 1 |
| BP | GO:0120192 | tight junction assembly | 1月10日 | 79/18866 | 0.041104 | 0.068775 | 0.025281 | TNF | 1 |
| BP | GO:1900034 | regulation of cellular response to heat | 1月10日 | 79/18866 | 0.041104 | 0.068775 | 0.025281 | MAPK3 | 1 |
| BP | GO:0002312 | B cell activation involved in immune response | 1月10日 | 80/18866 | 0.041614 | 0.069177 | 0.025429 | XBP1 | 1 |
| BP | GO:0014032 | neural crest cell development | 1月10日 | 80/18866 | 0.041614 | 0.069177 | 0.025429 | MAPK3 | 1 |
| BP | GO:0032204 | regulation of telomere maintenance | 1月10日 | 80/18866 | 0.041614 | 0.069177 | 0.025429 | MAPK3 | 1 |
| BP | GO:0055021 | regulation of cardiac muscle tissue growth | 1月10日 | 80/18866 | 0.041614 | 0.069177 | 0.025429 | G6PD | 1 |
| BP | GO:1901224 | positive regulation of NIK/NF-kappaB signaling | 1月10日 | 80/18866 | 0.041614 | 0.069177 | 0.025429 | TNF | 1 |
| BP | GO:0001937 | negative regulation of endothelial cell proliferation | 1月10日 | 81/18866 | 0.042124 | 0.069663 | 0.025608 | TNF | 1 |
| BP | GO:0010833 | telomere maintenance via telomere lengthening | 1月10日 | 81/18866 | 0.042124 | 0.069663 | 0.025608 | MAPK3 | 1 |
| BP | GO:0019915 | lipid storage | 1月10日 | 81/18866 | 0.042124 | 0.069663 | 0.025608 | TNF | 1 |
| BP | GO:0043242 | negative regulation of protein-containing complex disassembly | 1月10日 | 81/18866 | 0.042124 | 0.069663 | 0.025608 | TNF | 1 |
| BP | GO:0120193 | tight junction organization | 1月10日 | 82/18866 | 0.042634 | 0.070324 | 0.025851 | TNF | 1 |
| BP | GO:1903533 | regulation of protein targeting | 1月10日 | 82/18866 | 0.042634 | 0.070324 | 0.025851 | SREBF1 | 1 |
| BP | GO:0002718 | regulation of cytokine production involved in immune response | 1月10日 | 83/18866 | 0.043144 | 0.070982 | 0.026093 | TNF | 1 |
| BP | GO:0055013 | cardiac muscle cell development | 1月10日 | 83/18866 | 0.043144 | 0.070982 | 0.026093 | G6PD | 1 |
| BP | GO:0014031 | mesenchymal cell development | 1月10日 | 84/18866 | 0.043653 | 0.07127 | 0.026199 | MAPK3 | 1 |
| BP | GO:0030279 | negative regulation of ossification | 1月10日 | 84/18866 | 0.043653 | 0.07127 | 0.026199 | TNF | 1 |
| BP | GO:0033045 | regulation of sister chromatid segregation | 1月10日 | 84/18866 | 0.043653 | 0.07127 | 0.026199 | DUSP1 | 1 |
| BP | GO:0046209 | nitric oxide metabolic process | 1月10日 | 84/18866 | 0.043653 | 0.07127 | 0.026199 | TNF | 1 |
| BP | GO:0048864 | stem cell development | 1月10日 | 84/18866 | 0.043653 | 0.07127 | 0.026199 | MAPK3 | 1 |
| BP | GO:0050886 | endocrine process | 1月10日 | 84/18866 | 0.043653 | 0.07127 | 0.026199 | KRAS | 1 |
| BP | GO:0006970 | response to osmotic stress | 1月10日 | 85/18866 | 0.044162 | 0.071736 | 0.02637 | TNF | 1 |
| BP | GO:0043154 | negative regulation of cysteine-type endopeptidase activity involved in apoptotic process | 1月10日 | 85/18866 | 0.044162 | 0.071736 | 0.02637 | TNF | 1 |
| BP | GO:0043297 | apical junction assembly | 1月10日 | 85/18866 | 0.044162 | 0.071736 | 0.02637 | TNF | 1 |
| BP | GO:0110110 | positive regulation of animal organ morphogenesis | 1月10日 | 85/18866 | 0.044162 | 0.071736 | 0.02637 | XBP1 | 1 |
| BP | GO:0006919 | activation of cysteine-type endopeptidase activity involved in apoptotic process | 1月10日 | 86/18866 | 0.044671 | 0.072105 | 0.026506 | TNF | 1 |
| BP | GO:0014910 | regulation of smooth muscle cell migration | 1月10日 | 86/18866 | 0.044671 | 0.072105 | 0.026506 | NOX4 | 1 |
| BP | GO:0042509 | regulation of tyrosine phosphorylation of STAT protein | 1月10日 | 86/18866 | 0.044671 | 0.072105 | 0.026506 | TNF | 1 |
| BP | GO:0045445 | myoblast differentiation | 1月10日 | 86/18866 | 0.044671 | 0.072105 | 0.026506 | TNF | 1 |
| BP | GO:1903901 | negative regulation of viral life cycle | 1月10日 | 86/18866 | 0.044671 | 0.072105 | 0.026506 | TNF | 1 |
| BP | GO:0001942 | hair follicle development | 1月10日 | 87/18866 | 0.04518 | 0.072378 | 0.026606 | TNF | 1 |
| BP | GO:0009060 | aerobic respiration | 1月10日 | 87/18866 | 0.04518 | 0.072378 | 0.026606 | SIRT3 | 1 |
| BP | GO:0060420 | regulation of heart growth | 1月10日 | 87/18866 | 0.04518 | 0.072378 | 0.026606 | G6PD | 1 |
| BP | GO:1904705 | regulation of vascular associated smooth muscle cell proliferation | 1月10日 | 87/18866 | 0.04518 | 0.072378 | 0.026606 | TNF | 1 |
| BP | GO:1990874 | vascular associated smooth muscle cell proliferation | 1月10日 | 87/18866 | 0.04518 | 0.072378 | 0.026606 | TNF | 1 |
| BP | GO:2001057 | reactive nitrogen species metabolic process | 1月10日 | 87/18866 | 0.04518 | 0.072378 | 0.026606 | TNF | 1 |
| BP | GO:0014068 | positive regulation of phosphatidylinositol 3-kinase signaling | 1月10日 | 88/18866 | 0.045688 | 0.073101 | 0.026872 | TNF | 1 |
| BP | GO:0007260 | tyrosine phosphorylation of STAT protein | 1月10日 | 89/18866 | 0.046197 | 0.073183 | 0.026902 | TNF | 1 |
| BP | GO:0008625 | extrinsic apoptotic signaling pathway via death domain receptors | 1月10日 | 89/18866 | 0.046197 | 0.073183 | 0.026902 | TNF | 1 |
| BP | GO:0014033 | neural crest cell differentiation | 1月10日 | 89/18866 | 0.046197 | 0.073183 | 0.026902 | MAPK3 | 1 |
| BP | GO:0022404 | molting cycle process | 1月10日 | 89/18866 | 0.046197 | 0.073183 | 0.026902 | TNF | 1 |
| BP | GO:0022405 | hair cycle process | 1月10日 | 89/18866 | 0.046197 | 0.073183 | 0.026902 | TNF | 1 |
| BP | GO:0043506 | regulation of JUN kinase activity | 1月10日 | 89/18866 | 0.046197 | 0.073183 | 0.026902 | TNF | 1 |
| BP | GO:0043537 | negative regulation of blood vessel endothelial cell migration | 1月10日 | 89/18866 | 0.046197 | 0.073183 | 0.026902 | TNF | 1 |
| BP | GO:0055006 | cardiac cell development | 1月10日 | 89/18866 | 0.046197 | 0.073183 | 0.026902 | G6PD | 1 |
| BP | GO:0046427 | positive regulation of receptor signaling pathway via JAK-STAT | 1月10日 | 90/18866 | 0.046705 | 0.073623 | 0.027064 | TNF | 1 |
| BP | GO:0070542 | response to fatty acid | 1月10日 | 90/18866 | 0.046705 | 0.073623 | 0.027064 | SREBF1 | 1 |
| BP | GO:0098773 | skin epidermis development | 1月10日 | 90/18866 | 0.046705 | 0.073623 | 0.027064 | TNF | 1 |
| BP | GO:1903351 | cellular response to dopamine | 1月10日 | 90/18866 | 0.046705 | 0.073623 | 0.027064 | MAPK3 | 1 |
| BP | GO:0030901 | midbrain development | 1月10日 | 91/18866 | 0.047212 | 0.073968 | 0.027191 | G6PD | 1 |
| BP | GO:0045682 | regulation of epidermis development | 1月10日 | 91/18866 | 0.047212 | 0.073968 | 0.027191 | TNF | 1 |
| BP | GO:0046849 | bone remodeling | 1月10日 | 91/18866 | 0.047212 | 0.073968 | 0.027191 | NOX4 | 1 |
| BP | GO:0051492 | regulation of stress fiber assembly | 1月10日 | 91/18866 | 0.047212 | 0.073968 | 0.027191 | NOX4 | 1 |
| BP | GO:1903350 | response to dopamine | 1月10日 | 91/18866 | 0.047212 | 0.073968 | 0.027191 | MAPK3 | 1 |
| BP | GO:0045582 | positive regulation of T cell differentiation | 1月10日 | 92/18866 | 0.04772 | 0.07458 | 0.027416 | XBP1 | 1 |
| BP | GO:1905897 | regulation of response to endoplasmic reticulum stress | 1月10日 | 92/18866 | 0.04772 | 0.07458 | 0.027416 | XBP1 | 1 |
| BP | GO:0002532 | production of molecular mediator involved in inflammatory response | 1月10日 | 93/18866 | 0.048227 | 0.074734 | 0.027472 | TNF | 1 |
| BP | GO:0014909 | smooth muscle cell migration | 1月10日 | 93/18866 | 0.048227 | 0.074734 | 0.027472 | NOX4 | 1 |
| BP | GO:0032677 | regulation of interleukin-8 production | 1月10日 | 93/18866 | 0.048227 | 0.074734 | 0.027472 | TNF | 1 |
| BP | GO:0061097 | regulation of protein tyrosine kinase activity | 1月10日 | 93/18866 | 0.048227 | 0.074734 | 0.027472 | NOX4 | 1 |
| BP | GO:1903035 | negative regulation of response to wounding | 1月10日 | 93/18866 | 0.048227 | 0.074734 | 0.027472 | TNF | 1 |
| BP | GO:1904894 | positive regulation of receptor signaling pathway via STAT | 1月10日 | 93/18866 | 0.048227 | 0.074734 | 0.027472 | TNF | 1 |
| BP | GO:2000117 | negative regulation of cysteine-type endopeptidase activity | 1月10日 | 93/18866 | 0.048227 | 0.074734 | 0.027472 | TNF | 1 |
| BP | GO:0030641 | regulation of cellular pH | 1月10日 | 94/18866 | 0.048734 | 0.075247 | 0.027661 | MAPK3 | 1 |
| BP | GO:0051304 | chromosome separation | 1月10日 | 94/18866 | 0.048734 | 0.075247 | 0.027661 | DUSP1 | 1 |
| BP | GO:1904035 | regulation of epithelial cell apoptotic process | 1月10日 | 94/18866 | 0.048734 | 0.075247 | 0.027661 | TNF | 1 |
| BP | GO:0007589 | body fluid secretion | 1月10日 | 95/18866 | 0.049241 | 0.075846 | 0.027881 | XBP1 | 1 |
| BP | GO:0050709 | negative regulation of protein secretion | 1月10日 | 95/18866 | 0.049241 | 0.075846 | 0.027881 | SREBF1 | 1 |
| BP | GO:0002690 | positive regulation of leukocyte chemotaxis | 1月10日 | 96/18866 | 0.049747 | 0.076442 | 0.0281 | MAPK3 | 1 |
| BP | GO:0035249 | synaptic transmission, glutamatergic | 1月10日 | 96/18866 | 0.049747 | 0.076442 | 0.0281 | TNF | 1 |
| CC | GO:0045121 | membrane raft | 3月10日 | 329/19559 | 0.000518 | 0.007806 | 0.003287 | TNF,MAPK3,KRAS | 3 |
| CC | GO:0098857 | membrane microdomain | 3月10日 | 330/19559 | 0.000523 | 0.007806 | 0.003287 | TNF,MAPK3,KRAS | 3 |
| CC | GO:0098589 | membrane region | 3月10日 | 343/19559 | 0.000585 | 0.007806 | 0.003287 | TNF,MAPK3,KRAS | 3 |
| CC | GO:0005925 | focal adhesion | 3月10日 | 415/19559 | 0.001018 | 0.00861 | 0.003625 | MAPK3,NOX4,KRAS | 3 |
| CC | GO:0030055 | cell-substrate junction | 3月10日 | 423/19559 | 0.001076 | 0.00861 | 0.003625 | MAPK3,NOX4,KRAS | 3 |
| CC | GO:0009898 | cytoplasmic side of plasma membrane | 2月10日 | 164/19559 | 0.003009 | 0.020058 | 0.008445 | G6PD,KRAS | 2 |
| CC | GO:0098562 | cytoplasmic side of membrane | 2月10日 | 188/19559 | 0.003931 | 0.022463 | 0.009458 | G6PD,KRAS | 2 |
| CC | GO:0043020 | NADPH oxidase complex | 1月10日 | 12/19559 | 0.00612 | 0.030599 | 0.012884 | NOX4 | 1 |
| CC | GO:0031143 | pseudopodium | 1月10日 | 18/19559 | 0.009167 | 0.040742 | 0.017155 | MAPK3 | 1 |
| CC | GO:0097038 | perinuclear endoplasmic reticulum | 1月10日 | 22/19559 | 0.011194 | 0.044775 | 0.018853 | NOX4 | 1 |
| CC | GO:0001891 | phagocytic cup | 1月10日 | 27/19559 | 0.013722 | 0.049898 | 0.02101 | TNF | 1 |
| CC | GO:0009295 | nucleoid | 1月10日 | 43/19559 | 0.021773 | 0.061585 | 0.025931 | LONP1 | 1 |
| CC | GO:0042645 | mitochondrial nucleoid | 1月10日 | 43/19559 | 0.021773 | 0.061585 | 0.025931 | LONP1 | 1 |
| CC | GO:0005635 | nuclear envelope | 2月10日 | 473/19559 | 0.023095 | 0.061585 | 0.025931 | MAPK3,SREBF1 | 2 |
| CC | GO:0005759 | mitochondrial matrix | 2月10日 | 473/19559 | 0.023095 | 0.061585 | 0.025931 | SIRT3,LONP1 | 2 |
| CC | GO:0012507 | ER to Golgi transport vesicle membrane | 1月10日 | 62/19559 | 0.031258 | 0.076079 | 0.032033 | SREBF1 | 1 |
| CC | GO:0001725 | stress fiber | 1月10日 | 68/19559 | 0.034235 | 0.076079 | 0.032033 | NOX4 | 1 |
| CC | GO:0097517 | contractile actin filament bundle | 1月10日 | 68/19559 | 0.034235 | 0.076079 | 0.032033 | NOX4 | 1 |
| CC | GO:0032432 | actin filament bundle | 1月10日 | 76/19559 | 0.038193 | 0.078384 | 0.033004 | NOX4 | 1 |
| CC | GO:0042641 | actomyosin | 1月10日 | 79/19559 | 0.039673 | 0.078384 | 0.033004 | NOX4 | 1 |
| CC | GO:0005901 | caveola | 1月10日 | 82/19559 | 0.041152 | 0.078384 | 0.033004 | MAPK3 | 1 |
| CC | GO:0030134 | COPII-coated ER to Golgi transport vesicle | 1月10日 | 95/19559 | 0.047534 | 0.082483 | 0.03473 | SREBF1 | 1 |
| CC | GO:0034451 | centriolar satellite | 1月10日 | 95/19559 | 0.047534 | 0.082483 | 0.03473 | G6PD | 1 |
| CC | GO:0031234 | extrinsic component of cytoplasmic side of plasma membrane | 1月10日 | 99/19559 | 0.04949 | 0.082483 | 0.03473 | KRAS | 1 |
| MF | GO:0008330 | protein tyrosine/threonine phosphatase activity | 1月10日 | 10/18352 | 0.005437 | 0.05496 | 0.027404 | DUSP1 | 1 |
| MF | GO:0051880 | G-quadruplex DNA binding | 1月10日 | 10/18352 | 0.005437 | 0.05496 | 0.027404 | LONP1 | 1 |
| MF | GO:0005536 | glucose binding | 1月10日 | 11/18352 | 0.005979 | 0.05496 | 0.027404 | G6PD | 1 |
| MF | GO:0140299 | small molecule sensor activity | 1月10日 | 11/18352 | 0.005979 | 0.05496 | 0.027404 | NOX4 | 1 |
| MF | GO:0016175 | superoxide-generating NAD(P)H oxidase activity | 1月10日 | 13/18352 | 0.007063 | 0.05496 | 0.027404 | NOX4 | 1 |
| MF | GO:0017017 | MAP kinase tyrosine/serine/threonine phosphatase activity | 1月10日 | 13/18352 | 0.007063 | 0.05496 | 0.027404 | DUSP1 | 1 |
| MF | GO:0033549 | MAP kinase phosphatase activity | 1月10日 | 14/18352 | 0.007604 | 0.05496 | 0.027404 | DUSP1 | 1 |
| MF | GO:0070403 | NAD+ binding | 1月10日 | 14/18352 | 0.007604 | 0.05496 | 0.027404 | SIRT3 | 1 |
| MF | GO:0017136 | NAD-dependent histone deacetylase activity | 1月10日 | 15/18352 | 0.008145 | 0.05496 | 0.027404 | SIRT3 | 1 |
| MF | GO:0034979 | NAD-dependent protein deacetylase activity | 1月10日 | 16/18352 | 0.008686 | 0.05496 | 0.027404 | SIRT3 | 1 |
| MF | GO:0030275 | LRR domain binding | 1月10日 | 17/18352 | 0.009227 | 0.05496 | 0.027404 | KRAS | 1 |
| MF | GO:0004708 | MAP kinase kinase activity | 1月10日 | 18/18352 | 0.009767 | 0.05496 | 0.027404 | MAPK3 | 1 |
| MF | GO:0050664 | oxidoreductase activity, acting on NAD(P)H, oxygen as acceptor | 1月10日 | 19/18352 | 0.010308 | 0.05496 | 0.027404 | NOX4 | 1 |
| MF | GO:0004707 | MAP kinase activity | 1月10日 | 20/18352 | 0.010847 | 0.05496 | 0.027404 | MAPK3 | 1 |
| MF | GO:0070182 | DNA polymerase binding | 1月10日 | 20/18352 | 0.010847 | 0.05496 | 0.027404 | LONP1 | 1 |
| MF | GO:0004407 | histone deacetylase activity | 1月10日 | 25/18352 | 0.013543 | 0.06295 | 0.031388 | SIRT3 | 1 |
| MF | GO:0033558 | protein deacetylase activity | 1月10日 | 26/18352 | 0.014081 | 0.06295 | 0.031388 | SIRT3 | 1 |
| MF | GO:0005164 | tumor necrosis factor receptor binding | 1月10日 | 31/18352 | 0.016768 | 0.070799 | 0.035301 | TNF | 1 |
| MF | GO:0051019 | mitogen-activated protein kinase binding | 1月10日 | 33/18352 | 0.017841 | 0.071365 | 0.035584 | DUSP1 | 1 |
| MF | GO:0019825 | oxygen binding | 1月10日 | 36/18352 | 0.019449 | 0.072508 | 0.036154 | NOX4 | 1 |
| MF | GO:0043531 | ADP binding | 1月10日 | 38/18352 | 0.020519 | 0.072508 | 0.036154 | LONP1 | 1 |
| MF | GO:0019213 | deacetylase activity | 1月10日 | 41/18352 | 0.022123 | 0.072508 | 0.036154 | SIRT3 | 1 |
| MF | GO:0001784 | phosphotyrosine residue binding | 1月10日 | 42/18352 | 0.022657 | 0.072508 | 0.036154 | MAPK3 | 1 |
| MF | GO:0004712 | protein serine/threonine/tyrosine kinase activity | 1月10日 | 45/18352 | 0.024258 | 0.072508 | 0.036154 | MAPK3 | 1 |
| MF | GO:0008138 | protein tyrosine/serine/threonine phosphatase activity | 1月10日 | 45/18352 | 0.024258 | 0.072508 | 0.036154 | DUSP1 | 1 |
| MF | GO:0032813 | tumor necrosis factor receptor superfamily binding | 1月10日 | 48/18352 | 0.025856 | 0.072508 | 0.036154 | TNF | 1 |
| MF | GO:0004879 | nuclear receptor activity | 1月10日 | 52/18352 | 0.027983 | 0.072508 | 0.036154 | SREBF1 | 1 |
| MF | GO:0098531 | ligand-activated transcription factor activity | 1月10日 | 52/18352 | 0.027983 | 0.072508 | 0.036154 | SREBF1 | 1 |
| MF | GO:0045309 | protein phosphorylated amino acid binding | 1月10日 | 53/18352 | 0.028514 | 0.072508 | 0.036154 | MAPK3 | 1 |
| MF | GO:0050661 | NADP binding | 1月10日 | 54/18352 | 0.029045 | 0.072508 | 0.036154 | G6PD | 1 |
| MF | GO:0051287 | NAD binding | 1月10日 | 55/18352 | 0.029576 | 0.072508 | 0.036154 | SIRT3 | 1 |
| MF | GO:0097110 | scaffold protein binding | 1月10日 | 60/18352 | 0.032225 | 0.076534 | 0.038161 | MAPK3 | 1 |
| MF | GO:0016811 | hydrolase activity, acting on carbon-nitrogen (but not peptide) bonds, in linear amides | 1月10日 | 69/18352 | 0.036977 | 0.082655 | 0.041213 | SIRT3 | 1 |
| MF | GO:0048029 | monosaccharide binding | 1月10日 | 69/18352 | 0.036977 | 0.082655 | 0.041213 | G6PD | 1 |
| MF | GO:0019003 | GDP binding | 1月10日 | 74/18352 | 0.039608 | 0.084727 | 0.042246 | KRAS | 1 |
| MF | GO:0004722 | protein serine/threonine phosphatase activity | 1月10日 | 75/18352 | 0.040134 | 0.084727 | 0.042246 | DUSP1 | 1 |
| MF | GO:0050660 | flavin adenine dinucleotide binding | 1月10日 | 81/18352 | 0.043281 | 0.088901 | 0.044328 | NOX4 | 1 |
| MF | GO:0051219 | phosphoprotein binding | 1月10日 | 85/18352 | 0.045374 | 0.090748 | 0.045248 | MAPK3 | 1 |
| MF | GO:0003727 | single-stranded RNA binding | 1月10日 | 90/18352 | 0.047984 | 0.09117 | 0.045459 | LONP1 | 1 |
| MF | GO:0072341 | modified amino acid binding | 1月10日 | 90/18352 | 0.047984 | 0.09117 | 0.045459 | NOX4 | 1 |
