# Supplementary material for: Identification and characterization of ferroptosis-related genes in therapy-resistant gastric cancer
Source: Medicine (Baltimore). 2024 May 17;103(20):e38193. doi: 10.1097/MD.0000000000038193 (PMC11098190; doi:10.1097/MD.0000000000038193)
Supplement: Supplementary file 3 [file medi-103-e38193-s003.docx]

**Table S2** Kyoto Encyclopedia of Genes and Genomes enrichment analysis

| ID | Description | GeneRatio | BgRatio | pvalue | p.adjust | qvalue | geneID | Count |
| --- | --- | --- | --- | --- | --- | --- | --- | --- |
| hsa05230 | Central carbon metabolism in cancer | 4月9日 | 70/8112 | 6.20E-07 | 9.80E-05 | 3.20E-05 | 5595/23410/2539/3845 | 4 |
| hsa04933 | AGE-RAGE signaling pathway in diabetic complications | 4月9日 | 100/8112 | 2.61E-06 | 0.000206 | 6.74E-05 | 7124/5595/50507/3845 | 4 |
| hsa05010 | Alzheimer disease | 5月9日 | 384/8112 | 2.49E-05 | 0.001313 | 0.000429 | 7124/5595/50507/3845/7494 | 5 |
| hsa04664 | Fc epsilon RI signaling pathway | 3月9日 | 68/8112 | 4.57E-05 | 0.001721 | 0.000562 | 7124/5595/3845 | 3 |
| hsa05417 | Lipid and atherosclerosis | 4月9日 | 215/8112 | 5.45E-05 | 0.001721 | 0.000562 | 7124/5595/3845/7494 | 4 |
| hsa05022 | Pathways of neurodegeneration - multiple diseases | 5月9日 | 476/8112 | 7.05E-05 | 0.001856 | 0.000606 | 7124/5595/50507/3845/7494 | 5 |
| hsa04625 | C-type lectin receptor signaling pathway | 3月9日 | 104/8112 | 0.000163 | 0.003211 | 0.001048 | 7124/5595/3845 | 3 |
| hsa04660 | T cell receptor signaling pathway | 3月9日 | 104/8112 | 0.000163 | 0.003211 | 0.001048 | 7124/5595/3845 | 3 |
| hsa04010 | MAPK signaling pathway | 4月9日 | 294/8112 | 0.000184 | 0.003237 | 0.001057 | 7124/5595/3845/1843 | 4 |
| hsa04726 | Serotonergic synapse | 3月9日 | 115/8112 | 0.000219 | 0.003462 | 0.00113 | 5595/3845/1843 | 3 |
| hsa04071 | Sphingolipid signaling pathway | 3月9日 | 119/8112 | 0.000242 | 0.003482 | 0.001137 | 7124/5595/3845 | 3 |
| hsa04650 | Natural killer cell mediated cytotoxicity | 3月9日 | 131/8112 | 0.000322 | 0.004147 | 0.001354 | 7124/5595/3845 | 3 |
| hsa04210 | Apoptosis | 3月9日 | 136/8112 | 0.00036 | 0.004147 | 0.001354 | 7124/5595/3845 | 3 |
| hsa04910 | Insulin signaling pathway | 3月9日 | 137/8112 | 0.000367 | 0.004147 | 0.001354 | 5595/6720/3845 | 3 |
| hsa04936 | Alcoholic liver disease | 3月9日 | 142/8112 | 0.000408 | 0.004301 | 0.001404 | 7124/6720/50507 | 3 |
| hsa04150 | mTOR signaling pathway | 3月9日 | 155/8112 | 0.000528 | 0.004814 | 0.001571 | 7124/5595/3845 | 3 |
| hsa04932 | Non-alcoholic fatty liver disease | 3月9日 | 155/8112 | 0.000528 | 0.004814 | 0.001571 | 7124/6720/7494 | 3 |
| hsa05160 | Hepatitis C | 3月9日 | 157/8112 | 0.000548 | 0.004814 | 0.001571 | 7124/5595/3845 | 3 |
| hsa05161 | Hepatitis B | 3月9日 | 162/8112 | 0.000601 | 0.004999 | 0.001632 | 7124/5595/3845 | 3 |
| hsa04960 | Aldosterone-regulated sodium reabsorption | 2月9日 | 37/8112 | 0.000714 | 0.005374 | 0.001754 | 5595/3845 | 2 |
| hsa05216 | Thyroid cancer | 2月9日 | 37/8112 | 0.000714 | 0.005374 | 0.001754 | 5595/3845 | 2 |
| hsa05219 | Bladder cancer | 2月9日 | 41/8112 | 0.000877 | 0.006301 | 0.002057 | 5595/3845 | 2 |
| hsa04930 | Type II diabetes mellitus | 2月9日 | 46/8112 | 0.001104 | 0.007586 | 0.002476 | 7124/5595 | 2 |
| hsa05205 | Proteoglycans in cancer | 3月9日 | 205/8112 | 0.001194 | 0.007859 | 0.002566 | 7124/5595/3845 | 3 |
| hsa05170 | Human immunodeficiency virus 1 infection | 3月9日 | 212/8112 | 0.001316 | 0.008316 | 0.002715 | 7124/5595/3845 | 3 |
| hsa05166 | Human T-cell leukemia virus 1 infection | 3月9日 | 222/8112 | 0.001503 | 0.008819 | 0.002879 | 7124/5595/3845 | 3 |
| hsa05208 | Chemical carcinogenesis - reactive oxygen species | 3月9日 | 223/8112 | 0.001523 | 0.008819 | 0.002879 | 5595/50507/3845 | 3 |
| hsa05163 | Human cytomegalovirus infection | 3月9日 | 225/8112 | 0.001563 | 0.008819 | 0.002879 | 7124/5595/3845 | 3 |
| hsa05213 | Endometrial cancer | 2月9日 | 58/8112 | 0.001751 | 0.009542 | 0.003115 | 5595/3845 | 2 |
| hsa04370 | VEGF signaling pathway | 2月9日 | 59/8112 | 0.001812 | 0.009542 | 0.003115 | 5595/3845 | 2 |
| hsa04730 | Long-term depression | 2月9日 | 60/8112 | 0.001873 | 0.009548 | 0.003117 | 5595/3845 | 2 |
| hsa04929 | GnRH secretion | 2月9日 | 64/8112 | 0.002129 | 0.01051 | 0.003431 | 5595/3845 | 2 |
| hsa04720 | Long-term potentiation | 2月9日 | 67/8112 | 0.002331 | 0.01083 | 0.003536 | 5595/3845 | 2 |
| hsa05221 | Acute myeloid leukemia | 2月9日 | 67/8112 | 0.002331 | 0.01083 | 0.003536 | 5595/3845 | 2 |
| hsa05211 | Renal cell carcinoma | 2月9日 | 69/8112 | 0.00247 | 0.01115 | 0.00364 | 5595/3845 | 2 |
| hsa04917 | Prolactin signaling pathway | 2月9日 | 70/8112 | 0.002541 | 0.011153 | 0.003641 | 5595/3845 | 2 |
| hsa05218 | Melanoma | 2月9日 | 72/8112 | 0.002686 | 0.01117 | 0.003646 | 5595/3845 | 2 |
| hsa05223 | Non-small cell lung cancer | 2月9日 | 72/8112 | 0.002686 | 0.01117 | 0.003646 | 5595/3845 | 2 |
| hsa05214 | Glioma | 2月9日 | 75/8112 | 0.002912 | 0.011243 | 0.00367 | 5595/3845 | 2 |
| hsa05133 | Pertussis | 2月9日 | 76/8112 | 0.002989 | 0.011243 | 0.00367 | 7124/5595 | 2 |
| hsa05212 | Pancreatic cancer | 2月9日 | 76/8112 | 0.002989 | 0.011243 | 0.00367 | 5595/3845 | 2 |
| hsa05220 | Chronic myeloid leukemia | 2月9日 | 76/8112 | 0.002989 | 0.011243 | 0.00367 | 5595/3845 | 2 |
| hsa05140 | Leishmaniasis | 2月9日 | 77/8112 | 0.003066 | 0.011268 | 0.003678 | 7124/5595 | 2 |
| hsa01521 | EGFR tyrosine kinase inhibitor resistance | 2月9日 | 79/8112 | 0.003225 | 0.011581 | 0.003781 | 5595/3845 | 2 |
| hsa04662 | B cell receptor signaling pathway | 2月9日 | 82/8112 | 0.00347 | 0.012185 | 0.003978 | 5595/3845 | 2 |
| hsa04012 | ErbB signaling pathway | 2月9日 | 85/8112 | 0.003724 | 0.012792 | 0.004176 | 5595/3845 | 2 |
| hsa05210 | Colorectal cancer | 2月9日 | 86/8112 | 0.003811 | 0.01281 | 0.004182 | 5595/3845 | 2 |
| hsa04540 | Gap junction | 2月9日 | 88/8112 | 0.003986 | 0.013122 | 0.004284 | 5595/3845 | 2 |
| hsa05235 | PD-L1 expression and PD-1 checkpoint pathway in cancer | 2月9日 | 89/8112 | 0.004076 | 0.013142 | 0.00429 | 5595/3845 | 2 |
| hsa04912 | GnRH signaling pathway | 2月9日 | 93/8112 | 0.004442 | 0.013783 | 0.004499 | 5595/3845 | 2 |
| hsa04350 | TGF-beta signaling pathway | 2月9日 | 94/8112 | 0.004536 | 0.013783 | 0.004499 | 7124/5595 | 2 |
| hsa04657 | IL-17 signaling pathway | 2月9日 | 94/8112 | 0.004536 | 0.013783 | 0.004499 | 7124/5595 | 2 |
| hsa05165 | Human papillomavirus infection | 3月9日 | 331/8112 | 0.004707 | 0.013885 | 0.004533 | 7124/5595/3845 | 3 |
| hsa05215 | Prostate cancer | 2月9日 | 97/8112 | 0.004824 | 0.013885 | 0.004533 | 5595/3845 | 2 |
| hsa01522 | Endocrine resistance | 2月9日 | 98/8112 | 0.004921 | 0.013885 | 0.004533 | 5595/3845 | 2 |
| hsa05231 | Choline metabolism in cancer | 2月9日 | 98/8112 | 0.004921 | 0.013885 | 0.004533 | 5595/3845 | 2 |
| hsa04916 | Melanogenesis | 2月9日 | 101/8112 | 0.00522 | 0.01425 | 0.004652 | 5595/3845 | 2 |
| hsa04914 | Progesterone-mediated oocyte maturation | 2月9日 | 102/8112 | 0.005321 | 0.01425 | 0.004652 | 5595/3845 | 2 |
| hsa05142 | Chagas disease | 2月9日 | 102/8112 | 0.005321 | 0.01425 | 0.004652 | 7124/5595 | 2 |
| hsa04620 | Toll-like receptor signaling pathway | 2月9日 | 104/8112 | 0.005526 | 0.014553 | 0.004751 | 7124/5595 | 2 |
| hsa04931 | Insulin resistance | 2月9日 | 108/8112 | 0.005948 | 0.015407 | 0.00503 | 7124/6720 | 2 |
| hsa04668 | TNF signaling pathway | 2月9日 | 112/8112 | 0.006384 | 0.016011 | 0.005227 | 7124/5595 | 2 |
| hsa05145 | Toxoplasmosis | 2月9日 | 112/8112 | 0.006384 | 0.016011 | 0.005227 | 7124/5595 | 2 |
| hsa04725 | Cholinergic synapse | 2月9日 | 113/8112 | 0.006496 | 0.016036 | 0.005235 | 5595/3845 | 2 |
| hsa04722 | Neurotrophin signaling pathway | 2月9日 | 119/8112 | 0.007182 | 0.017193 | 0.005613 | 5595/3845 | 2 |
| hsa04935 | Growth hormone synthesis, secretion and action | 2月9日 | 119/8112 | 0.007182 | 0.017193 | 0.005613 | 5595/3845 | 2 |
| hsa04919 | Thyroid hormone signaling pathway | 2月9日 | 121/8112 | 0.007418 | 0.017493 | 0.005711 | 5595/3845 | 2 |
| hsa04380 | Osteoclast differentiation | 2月9日 | 128/8112 | 0.008271 | 0.019219 | 0.006274 | 7124/5595 | 2 |
| hsa04926 | Relaxin signaling pathway | 2月9日 | 129/8112 | 0.008397 | 0.019227 | 0.006277 | 5595/3845 | 2 |
| hsa04068 | FoxO signaling pathway | 2月9日 | 131/8112 | 0.00865 | 0.019525 | 0.006374 | 5595/3845 | 2 |
| hsa05135 | Yersinia infection | 2月9日 | 137/8112 | 0.009431 | 0.020707 | 0.00676 | 7124/5595 | 2 |
| hsa04915 | Estrogen signaling pathway | 2月9日 | 138/8112 | 0.009564 | 0.020707 | 0.00676 | 5595/3845 | 2 |
| hsa04371 | Apelin signaling pathway | 2月9日 | 139/8112 | 0.009698 | 0.020707 | 0.00676 | 5595/3845 | 2 |
| hsa05418 | Fluid shear stress and atherosclerosis | 2月9日 | 139/8112 | 0.009698 | 0.020707 | 0.00676 | 7124/1843 | 2 |
| hsa04140 | Autophagy - animal | 2月9日 | 141/8112 | 0.009969 | 0.021001 | 0.006856 | 5595/3845 | 2 |
| hsa04550 | Signaling pathways regulating pluripotency of stem cells | 2月9日 | 143/8112 | 0.010243 | 0.021295 | 0.006952 | 5595/3845 | 2 |
| hsa05224 | Breast cancer | 2月9日 | 147/8112 | 0.010801 | 0.022164 | 0.007235 | 5595/3845 | 2 |
| hsa04072 | Phospholipase D signaling pathway | 2月9日 | 148/8112 | 0.010943 | 0.022166 | 0.007236 | 5595/3845 | 2 |
| hsa05226 | Gastric cancer | 2月9日 | 149/8112 | 0.011085 | 0.022171 | 0.007238 | 5595/3845 | 2 |
| hsa04921 | Oxytocin signaling pathway | 2月9日 | 154/8112 | 0.01181 | 0.023325 | 0.007615 | 5595/3845 | 2 |
| hsa04218 | Cellular senescence | 2月9日 | 156/8112 | 0.012106 | 0.023614 | 0.007709 | 5595/3845 | 2 |
| hsa05225 | Hepatocellular carcinoma | 2月9日 | 168/8112 | 0.01395 | 0.026879 | 0.008775 | 5595/3845 | 2 |
| hsa05164 | Influenza A | 2月9日 | 171/8112 | 0.014429 | 0.027467 | 0.008967 | 7124/5595 | 2 |
| hsa05152 | Tuberculosis | 2月9日 | 180/8112 | 0.015909 | 0.029925 | 0.009769 | 7124/5595 | 2 |
| hsa04360 | Axon guidance | 2月9日 | 182/8112 | 0.016247 | 0.030201 | 0.009859 | 5595/3845 | 2 |
| hsa04621 | NOD-like receptor signaling pathway | 2月9日 | 184/8112 | 0.016588 | 0.030476 | 0.009949 | 7124/5595 | 2 |
| hsa05034 | Alcoholism | 2月9日 | 187/8112 | 0.017105 | 0.031065 | 0.010141 | 5595/3845 | 2 |
| hsa04062 | Chemokine signaling pathway | 2月9日 | 192/8112 | 0.017983 | 0.032287 | 0.01054 | 5595/3845 | 2 |
| hsa05167 | Kaposi sarcoma-associated herpesvirus infection | 2月9日 | 194/8112 | 0.018339 | 0.032557 | 0.010628 | 5595/3845 | 2 |
| hsa05130 | Pathogenic Escherichia coli infection | 2月9日 | 197/8112 | 0.018879 | 0.033144 | 0.01082 | 7124/5595 | 2 |
| hsa05203 | Viral carcinogenesis | 2月9日 | 204/8112 | 0.020167 | 0.035015 | 0.011431 | 5595/3845 | 2 |
| hsa04015 | Rap1 signaling pathway | 2月9日 | 210/8112 | 0.021299 | 0.03658 | 0.011941 | 5595/3845 | 2 |
| hsa05207 | Chemical carcinogenesis - receptor activation | 2月9日 | 212/8112 | 0.021683 | 0.036838 | 0.012026 | 5595/3845 | 2 |
| hsa04810 | Regulation of actin cytoskeleton | 2月9日 | 218/8112 | 0.022851 | 0.03841 | 0.012539 | 5595/3845 | 2 |
| hsa04014 | Ras signaling pathway | 2月9日 | 232/8112 | 0.025679 | 0.042264 | 0.013797 | 5595/3845 | 2 |
| hsa05171 | Coronavirus disease - COVID-19 | 2月9日 | 232/8112 | 0.025679 | 0.042264 | 0.013797 | 7124/5595 | 2 |
| hsa05131 | Shigellosis | 2月9日 | 247/8112 | 0.028864 | 0.047015 | 0.015348 | 7124/5595 | 2 |
| hsa05132 | Salmonella infection | 2月9日 | 249/8112 | 0.0293 | 0.047239 | 0.015421 | 7124/5595 | 2 |
| hsa00030 | Pentose phosphate pathway | 1月9日 | 30/8112 | 0.032812 | 0.05181 | 0.016913 | 2539 | 1 |
| hsa01523 | Antifolate resistance | 1月9日 | 30/8112 | 0.032812 | 0.05181 | 0.016913 | 7124 | 1 |
| hsa05012 | Parkinson disease | 2月9日 | 266/8112 | 0.033119 | 0.05181 | 0.016913 | 1843/7494 | 2 |
| hsa05310 | Asthma | 1月9日 | 31/8112 | 0.033889 | 0.052495 | 0.017137 | 7124 | 1 |
| hsa05020 | Prion disease | 2月9日 | 273/8112 | 0.034747 | 0.053302 | 0.0174 | 7124/5595 | 2 |
| hsa00760 | Nicotinate and nicotinamide metabolism | 1月9日 | 35/8112 | 0.038186 | 0.058014 | 0.018939 | 23410 | 1 |
| hsa05143 | African trypanosomiasis | 1月9日 | 37/8112 | 0.040329 | 0.060685 | 0.019811 | 7124 | 1 |
| hsa05330 | Allograft rejection | 1月9日 | 38/8112 | 0.041398 | 0.061707 | 0.020144 | 7124 | 1 |
| hsa05206 | MicroRNAs in cancer | 2月9日 | 310/8112 | 0.043875 | 0.064787 | 0.02115 | 5595/3845 | 2 |
| hsa05332 | Graft-versus-host disease | 1月9日 | 42/8112 | 0.045666 | 0.066808 | 0.021809 | 7124 | 1 |
| hsa04940 | Type I diabetes mellitus | 1月9日 | 43/8112 | 0.046731 | 0.067738 | 0.022113 | 7124 | 1 |
