# Supplementary material for: Identification and characterization of ferroptosis-related genes in therapy-resistant gastric cancer
Source: Medicine (Baltimore). 2024 May 17;103(20):e38193. doi: 10.1097/MD.0000000000038193 (PMC11098190; doi:10.1097/MD.0000000000038193)
Supplement: Supplementary file 4 [file medi-103-e38193-s004.docx]

**Table S3** Gene set enrichment analysis of the GSE31811 dataset

| **setSize** | **enrichmentScore** | **NES** | **pvalue** | **qvalues** | **p.adjust** | **rank** | **leading_edge** | **core_enrichment** |
| --- | --- | --- | --- | --- | --- | --- | --- | --- |
| 124 | 0.322332 | 2.420639 | 3.39E-06 | 0.002549 | 0.002441 | 644 | tags=71%, list=48%, signal=41% | RALY/CPSF4/SF1/HABP4/RPS2/RPL7L1/UBTF/LUC7L3/SKIV2L/EIF4B/SUGP2/NUDT1/DDX17/THOC1/SOX2/SNRPN/RPL35/ARF1/USP10/RBM42/RPS9/RPS14/RPS2/GOLGB1/WDR33/NBPF10/C7orf50/FUS/GNL3L/ARL6IP4/RBM4/CDK11B/TRMT1/NSUN5P2/PPP5C/EIF4B/LARP4/DDX51/SOX4/EEF1D/SNRPA/MRPL1/MRPL11/TUFM/EMG1/DIDO1/GOT2/CSTF3/ARHGEF1/EEF1D/PPP1R8/NBPF10/TRMT6/RPS2/NBPF10/RPL13A/RPS5/STXBP1/STIP1/SNUPN/FXR1/RBM38/DIS3/CIRBP/NCOA5/TRIM28/NHP2/MYH9/RPL18A/MCRS1/RCC1L/LARP1/RPS14/RPL22/SPEN/HSPA1A/DDX31/MTRF1/SRRM2/TCP1/DIS3L/CARHSP1/EXOSC6/RBM5/REPIN1/EEF1A1/NR0B1/METTL1/ADAT1/DDX11/HMGB3/FAM50A/CIRBP/WDR46/ADAT1/CIRBP/LONP1/PPIE/P4HB |
| 68 | 0.38981 | 2.566642 | 3.78E-06 | 0.002549 | 0.002441 | 509 | tags=65%, list=38%, signal=43% | RALY/CPSF4/TXNL4B/SF1/HABP4/RPS2/LUC7L3/AKT1/SKIV2L/SUGP2/DDX17/THOC1/SNRPN/RPL35/MAPKAPK2/TTF2/RBM42/RPS9/RPS14/RPS2/WDR33/FUS/PSMC4/ARL6IP4/RBM4/CDK11B/PSMB3/SNRPA/CSTF3/TIRAP/PPP1R8/TRMT6/RPS2/RPL13A/NBAS/RPS5/XAB2/PQBP1/SNUPN/FXR1/RBM38/DIS3/CIRBP/RPL18A/LARP1/RPS14/RPL22/SPEN/HSPA1A |
| 40 | 0.418398 | 2.35712 | 0.00018 | 0.055782 | 0.053407 | 428 | tags=60%, list=32%, signal=42% | RALY/CPSF4/TXNL4B/SF1/HABP4/LUC7L3/SUGP2/DDX17/THOC1/SNRPN/TTF2/RBM42/WDR33/FUS/ARL6IP4/RBM4/CDK11B/SNRPA/CSTF3/PPP1R8/XAB2/PQBP1/SNUPN/FXR1/RBM38/CIRBP |
| 11 | 0.636707 | 2.233079 | 0.000207 | 0.055782 | 0.053407 | 492 | tags=93%, list=37%, signal=60% | RPS2/RPL7L1/RPL35/RPS9/RPS14/RPS2/LARP4/MRPL1/RPS2/RPL13A/RPS5/RPL18A/RPS14/RPL22 |
| 11 | 0.636707 | 2.233079 | 0.000207 | 0.055782 | 0.053407 | 492 | tags=93%, list=37%, signal=60% | RPS2/RPL7L1/RPL35/RPS9/RPS14/RPS2/MRPL1/MRPL11/RPS2/RPL13A/RPS5/RPL18A/RPS14/RPL22 |
| 14 | 0.583338 | 2.277778 | 0.000471 | 0.083376 | 0.079827 | 492 | tags=81%, list=37%, signal=52% | POM121/POM121/RPS2/RPL35/TRIM62/RPS9/RPS14/RPS2/RPS2/SEC13/RPL13A/RPS5/NUP210/RPL18A/NUP93/RPS14/RPL22 |
| 10 | 0.636226 | 2.132259 | 0.000559 | 0.083376 | 0.079827 | 492 | tags=93%, list=37%, signal=59% | RPS2/SKIV2L/RPL35/RPS9/RPS14/RPS2/RPS2/RPL13A/NBAS/RPS5/RPL18A/RPS14/RPL22 |
| 77 | 0.319649 | 2.165343 | 0.00057 | 0.083376 | 0.079827 | 622 | tags=69%, list=47%, signal=39% | RALY/CPSF4/TXNL4B/SF1/HABP4/RPS2/RPL7L1/LUC7L3/SUGP2/DDX17/THOC1/SNRPN/RPL35/TTF2/RBM42/RPS9/RPS14/RPS2/WDR33/FUS/ARL6IP4/RBM4/CDK11B/TRMT1/NSUN5P2/DDX51/SNRPA/MRPL1/EMG1/CSTF3/PPP1R8/TRMT6/RPS2/XAB2/PQBP1/SNUPN/FXR1/URM1/RBM38/DIS3/CIRBP/WDR74/NHP2/THADA/RPS14/METTL15/SPEN/HSPA1A/SRRM2/CDKAL1/CDK5RAP1/EXOSC6/RBM5/METTL1/ADAT1/CIRBP/WDR46/ADAT1/CIRBP |
| 13 | 0.585161 | 2.220757 | 0.000675 | 0.083376 | 0.079827 | 492 | tags=88%, list=37%, signal=56% | RPS2/RPL7L1/RPL35/RPS9/RPS14/RPS2/LARP4/MRPL1/MRPL11/RPS2/RPL13A/RPS5/RPL18A/RPS14/RPL22 |
| 32 | 0.412373 | 2.175534 | 0.000779 | 0.083376 | 0.079827 | 638 | tags=82%, list=48%, signal=44% | RPS2/AKT1/SKIV2L/RPL35/MAPKAPK2/RPS9/RPS14/RPS2/FUS/PSMC4/PSMB3/TIRAP/PPP1R8/RPS2/RPL13A/NBAS/RPS5/FXR1/DIS3/CIRBP/RPL18A/LARP1/RPS14/RPL22/HSPA1A/DIS3L/CARHSP1/EXOSC6/CIRBP/CIRBP/FEN1 |
| 12 | 0.594213 | 2.166703 | 0.000787 | 0.083376 | 0.079827 | 143 | tags=58%, list=11%, signal=53% | ITGB8/FUT1/CX3CL1/XBP1/GHRL/PDCL3/GPER1 |
| 18 | 0.509816 | 2.187637 | 0.000796 | 0.083376 | 0.079827 | 492 | tags=82%, list=37%, signal=53% | SF1/RPS2/RPL7L1/RPL35/RPS9/RPS14/RPS2/LARP4/MRPL1/MRPL11/RPS2/RPL13A/RPS5/MTG2/RPL18A/LARP1/RPS14/RPL22 |
| 130 | 0.256643 | 1.936079 | 0.000803 | 0.083376 | 0.079827 | 325 | tags=40%, list=24%, signal=34% | E4F1/TXNL4B/MCM8/CDK16/ZNF503/NABP2/GOLGA8A/UNC119/CTBP1/SYCP2/AKT1/AKT2/THOC1/SGSM3/SOX2/UBD/BEX2/CCNF/KLHL42/ARF1/MCM8/ACTR1A/STAT5B/PKD1/GPER1/LIG1/MDM4/OFD1/ACTR1A/NCAPH2/PMF1/PSMC4/USP22/CDK11B/MAD2L2/CDK5RAP3/PPP5C/GIT1/NSFL1C/MAX/ILK/TUBGCP3/MGA/PPME1/SPC24/SOX4/AKAP9/PSMB3/NAA10/CDC42/NSFL1C/BIRC5/PPP1R13B/RASSF4/CCSAP |
| 75 | 0.302751 | 2.043371 | 0.000913 | 0.087972 | 0.084227 | 448 | tags=52%, list=34%, signal=37% | VCX/HABP4/NEPRO/VCX3A/RPL7L1/UBTF/DDX17/RPL35/UBD/ZNF593/RPS9/RPS14/MPHOSPH8/WDR33/GNL3L/NARF/FAM193B/ARL6IP4/RBM4/MAD2L2/CDK5RAP3/NSUN5P2/KIAA0319L/AFF4/DDX51/SPC24/EEF1D/ABHD14B/NAA10/EMG1/EEF1D/RPL13A/MLLT1/NIN/SP140/FXR1/DCTN3/NFIC/ARL2/WDR74/NHP2/MCRS1 |
| 83 | 0.295469 | 2.039274 | 0.001105 | 0.099361 | 0.095132 | 716 | tags=74%, list=54%, signal=37% | MAGEA2B/RPS2/XBP1/AKT1/SKIV2L/WWP2/RPL35/UBD/CCNF/KLHL42/USP10/PDCL3/PKD1/MAPKAPK2/VGLL4/RPS9/RPS14/RPS2/FUS/PSMC4/USP22/CDK5RAP3/NSFL1C/PSMB3/NSFL1C/TIRAP/PPP1R8/BCAP31/DDA1/RPS2/PSAP/RPL13A/NBAS/RPS5/NFE2L1/UBE2D4/ATP13A2/FXR1/DIS3/CIRBP/RPL18A/LARP1/AP2S1/RPS14/RNF121/RPL22/UBXN1/USP5/HSPA1A/PINK1/RGN/ASB2/DIS3L/DFFB/CARHSP1/EXOSC6/GID4/TMUB2/CIRBP/CIRBP/LONP1/FEN1/ARAF/AMFR/TMUB2/UBA7/UBXN11/KLHL22/PSAP/UBE2B |
| 42 | 0.367914 | 2.110715 | 0.001265 | 0.106637 | 0.102098 | 428 | tags=56%, list=32%, signal=39% | RALY/CPSF4/TXNL4B/SF1/HABP4/LUC7L3/SUGP2/DDX17/THOC1/SNRPN/TTF2/RBM42/WDR33/FUS/ARL6IP4/RBM4/SNRPA/CSTF3/PPP1R8/XAB2/PQBP1/SNUPN/FXR1/RBM38/CIRBP |
| 74 | 0.295806 | 1.987556 | 0.001613 | 0.127981 | 0.122534 | 300 | tags=43%, list=22%, signal=36% | E4F1/MCM8/CDK16/NABP2/UNC119/AKT1/UBD/CCNF/ARF1/MCM8/ACTR1A/STAT5B/PKD1/LIG1/MDM4/OFD1/ACTR1A/NCAPH2/PSMC4/USP22/CDK11B/MAD2L2/CDK5RAP3/PPP5C/NSFL1C/MAX/TUBGCP3/PPME1/SOX4/AKAP9/PSMB3/NAA10/CDC42/NSFL1C/BIRC5 |
| 82 | 0.281468 | 1.941128 | 0.001936 | 0.145102 | 0.138925 | 525 | tags=58%, list=39%, signal=38% | CD74/TOM1/CPSF4/POM121/POM121/RPS2/AKT1/AKT2/THOC1/SGSM3/RPL35/ARF1/PKD1/SNX12/RPS9/RPS14/RILPL2/RPS2/NUTF2/WDR33/CDC42/PPP1R13B/LSG1/TMED9/BCAP31/STX4/RPS2/SEC13/PSAP/RPL13A/RPS5/MIEF1/STXBP1/NUDT19/SNUPN/ATP13A2/PMM1/NUP210/SNX17/TRIM28/RPL18A/APPBP2/DHRS4/GRIPAP1/AP2S1/NUP93/SEC23B/RPS14/RPL22/MYO1C/PINK1/SYNGR1 |
| 53 | 0.330989 | 2.043184 | 0.0021 | 0.149082 | 0.142736 | 637 | tags=72%, list=48%, signal=39% | RALY/TXNL4B/SF1/RPS2/RPL7L1/LUC7L3/DDX17/SNRPN/RPL35/TTF2/RBM42/RPS9/RPS14/RPS2/FUS/GNL3L/LARP4/SNRPA/MRPL1/MRPL11/EMG1/PPP1R8/RPS2/RPL13A/RPS5/XAB2/FXR1/CIRBP/WDR74/NHP2/RPL18A/MCRS1/LARP1/RPS14/RPL22/HSPA1A/SRRM2/RBM5/NR0B1/CIRBP/WDR46/CIRBP/PPIE |
| 29 | 0.401761 | 2.063374 | 0.00225 | 0.151791 | 0.14533 | 622 | tags=75%, list=47%, signal=41% | CPSF4/SF1/AKT1/DDX17/MAPKAPK2/RBM42/FUS/PSMC4/RBM4/CDK11B/PSMB3/SNRPA/TIRAP/NBAS/FXR1/DIS3/CIRBP/LARP1/HSPA1A/CARHSP1/EXOSC6/RBM5/CIRBP/CIRBP |
| 28 | 0.415763 | 2.108592 | 0.002537 | 0.16297 | 0.156033 | 522 | tags=73%, list=39%, signal=45% | VCX/RPS2/RPL7L1/DDX17/RPL35/RPS9/RPS14/RPS2/GNL3L/NSUN5P2/DDX51/MRPL1/MRPL11/EMG1/LSG1/RPS2/RPS5/DIS3/MTG2/WDR74/NHP2/RPS14/METTL15/DDX31 |
| 29 | 0.387112 | 1.988138 | 0.003579 | 0.208635 | 0.199754 | 670 | tags=78%, list=50%, signal=40% | RALY/CPSF4/TXNL4B/SF1/LUC7L3/DDX17/SNRPN/RBM42/WDR33/FUS/RBM4/SNRPA/CSTF3/XAB2/PQBP1/SNUPN/FXR1/CIRBP/SPEN/SRRM2/RBM5/CIRBP/CIRBP/PPIE/DBR1 |
| 81 | 0.283543 | 1.944109 | 0.003615 | 0.208635 | 0.199754 | 509 | tags=53%, list=38%, signal=35% | E4F1/CD74/CPSF4/POM121/POM121/RPS2/ZNF683/PI4KB/CTBP1/THOC1/WWP2/RPL35/ARF1/TRIM62/RPS9/RPS14/RPS2/MPHOSPH8/ATF7IP/ANPEP/UCKL1/CDK5RAP3/KIAA0319L/FN1/PSMB3/F2/CDC42/TUFM/PGLYRP1/IGF2R/BCAP31/RPS2/SEC13/RPL13A/ITGAL/RPS5/NUP210/RBM38/TRIM28/KLC1/RPL18A/MCRS1/LARP1/AP2S1/NUP93/RPS14/RPL22/UBXN1/SPEN/HSPA1A |
| 41 | 0.363153 | 2.065851 | 0.003712 | 0.208635 | 0.199754 | 437 | tags=60%, list=33%, signal=42% | VCX/SF1/RPS2/RPL7L1/LUC7L3/EIF4B/DDX17/RPL35/RPS9/RPS14/RPS2/GNL3L/NSUN5P2/EIF4B/DDX51/MRPL1/MRPL11/EMG1/LSG1/RPS2/RPL13A/RPS5/XAB2/SNUPN/DIS3/MTG2/WDR74/NHP2 |
| 14 | 0.516624 | 2.017278 | 0.003966 | 0.214022 | 0.204912 | 229 | tags=53%, list=17%, signal=45% | SF1/SYCP2/COL9A3/NUDT1/BMPR1B/STAT5B/PKD1/HOXA10 |
| 13 | 0.516428 | 1.959906 | 0.00415 | 0.21489 | 0.205743 | 492 | tags=82%, list=37%, signal=53% | RPS2/SKIV2L/RPL35/RPS9/RPS14/RPS2/RPS2/RPL13A/NBAS/RPS5/DIS3/RPL18A/RPS14/RPL22 |
| 34 | 0.3621 | 1.945479 | 0.004301 | 0.21489 | 0.205743 | 137 | tags=33%, list=10%, signal=31% | ITGB8/SF1/SYCP2/COL9A3/AKT1/NUDT1/ASCL2/BMPR1B/CCNF/GHRL/STAT5B/PKD1 |
| 104 | 0.255658 | 1.858613 | 0.004772 | 0.226649 | 0.217001 | 669 | tags=66%, list=50%, signal=36% | MAGEA2B/FMOD/RPS2/XBP1/AKT1/SKIV2L/SGSM3/WWP2/RPL35/UBD/CCNF/KLHL42/USP10/PDCL3/PKD1/MAPKAPK2/VGLL4/SNX12/RPS9/RPS14/MDM4/RPS2/ATG4B/FUS/PSMC4/USP22/MAD2L2/CDK5RAP3/NSFL1C/PSMB3/NSFL1C/PGLYRP1/TIRAP/PPP1R8/BCAP31/DDA1/RPS2/PSAP/RPL13A/NBAS/RPS5/NFE2L1/UBE2D4/ATP13A2/FXR1/AGRN/DIS3/CIRBP/RPL18A/LARP1/AP2S1/RPS14/RNF121/RPL22/UBXN1/USP5/HSPA1A/PINK1/RGN/ASB2/DIS3L/DFFB/CARHSP1/EXOSC6/EEF1A1/GID4/TMUB2/CIRBP/CIRBP/LONP1/FEN1/ARAF/AMFR/HSPG2/TMUB2/UBA7 |
| 49 | 0.321892 | 1.932404 | 0.004872 | 0.226649 | 0.217001 | 638 | tags=70%, list=48%, signal=38% | RPS2/ACOT7/AKT1/SKIV2L/NUDT1/RPL35/MAPKAPK2/RPS9/RPS14/RPS2/FUS/PSMC4/PSMB3/OGG1/AKR7A3/TIRAP/PPP1R8/RPS2/RPL13A/NBAS/RPS5/FXR1/SNX17/DIS3/CIRBP/RPL18A/LARP1/RPS14/RPL22/CYP27A1/HSPA1A/RGN/DIS3L/DFFB/CARHSP1/EXOSC6/CIRBP/CIRBP/FEN1 |
| 85 | 0.259285 | 1.799378 | 0.005913 | 0.265895 | 0.254577 | 325 | tags=42%, list=24%, signal=34% | E4F1/CDK16/NABP2/CTBP1/AKT1/AKT2/THOC1/SGSM3/SOX2/UBD/BEX2/CCNF/ACTR1A/STAT5B/PKD1/GPER1/MDM4/OFD1/ACTR1A/PSMC4/USP22/CDK11B/MAD2L2/CDK5RAP3/GIT1/NSFL1C/MAX/ILK/MGA/SOX4/AKAP9/PSMB3/NAA10/CDC42/NSFL1C/PPP1R13B/CCSAP |
| 64 | 0.296623 | 1.923701 | 0.006269 | 0.270576 | 0.259058 | 521 | tags=58%, list=39%, signal=37% | E4F1/MAGEA2B/POM121/POM121/RPS2/SOCS7/AKT1/PPIL2/WWP2/UBD/CCNF/KLHL42/TRIM62/RPS2/GNL3L/DCAF7/PSMC4/USP22/MAD2L2/CDK5RAP3/AKTIP/SOX4/DCAF8/PSMB3/CDC42/MED10/DDA1/RPS2/MIB2/NFE2L1/UBE2D4/NUP210/URM1/TRIM28/NUP93/RNF121/BCL11A/UBXN1/USP5/HSPA1A/PINK1 |
| 29 | 0.375538 | 1.928697 | 0.006418 | 0.270576 | 0.259058 | 481 | tags=62%, list=36%, signal=41% | CD74/HLA-DQA1/HLA-DQA1/ARF1/ACTR1A/ACTR1A/PSMC4/HLA-DQA2/PSMB3/HLA-DQB1/HLA-DRB5/DYNC1LI2/HLA-DMA/BCAP31/SEC13/PSAP/DCTN3/KLC1/HLA-DRB4/TAPBP/AP2S1 |
| 85 | 0.255839 | 1.775463 | 0.007459 | 0.304917 | 0.291938 | 699 | tags=69%, list=52%, signal=35% | ITGB8/PAIP2B/HMGCS2/HABP4/RPS2/ACOT7/AKT1/EIF4B/AKT2/RPL35/GSTM4/PDPR/ACLY/NARS2/RPS9/RPS14/RPS2/GSTM2/ANPEP/ACOT11/RBM4/EIF4B/LARP4/CNDP2/SOX4/EEF1D/MRPL1/MRPL11/TUFM/CTNS/EEF1D/RPS2/PSAP/PDPR/RPL13A/RPS5/NFE2L1/ACACB/FXR1/CIRBP/MTG2/ACSBG1/RPL18A/GSTM1/DLST/RCC1L/LARP1/TAPBP/RPS14/RPL22/PDP2/PINK1/MTRF1/CDKAL1/CDK5RAP1/EEF1A1/RPS6KB2/CIRBP/CIRBP/ASAH1/SIRT3/AASS/FOLR1/PMVK/PPP1R15A/OGDH/ASRGL1/EEF1D |
| 63 | 0.285067 | 1.836898 | 0.0078 | 0.309474 | 0.296301 | 586 | tags=62%, list=44%, signal=37% | PAIP2B/HABP4/RPS2/AKT1/EIF4B/AKT2/RPL35/GSTM4/NARS2/RPS9/RPS14/RPS2/GSTM2/ANPEP/RBM4/EIF4B/LARP4/CNDP2/SOX4/EEF1D/MRPL1/MRPL11/TUFM/CTNS/EEF1D/RPS2/RPL13A/RPS5/NFE2L1/FXR1/CIRBP/MTG2/RPL18A/GSTM1/RCC1L/LARP1/TAPBP/RPS14/RPL22/PINK1/MTRF1/CDKAL1/CDK5RAP1/EEF1A1/RPS6KB2 |
| 10 | 0.528913 | 1.772608 | 0.008487 | 0.327129 | 0.313204 | 229 | tags=60%, list=17%, signal=50% | SF1/COL9A3/NUDT1/BMPR1B/STAT5B/HOXA10 |
| 45 | 0.315101 | 1.846795 | 0.008854 | 0.331141 | 0.317046 | 638 | tags=71%, list=48%, signal=38% | RPS2/ACOT7/AKT1/SKIV2L/NUDT1/RPL35/MAPKAPK2/RPS9/RPS14/RPS2/FUS/PSMC4/PSMB3/OGG1/TIRAP/PPP1R8/RPS2/RPL13A/NBAS/RPS5/FXR1/DIS3/CIRBP/RPL18A/LARP1/RPS14/RPL22/HSPA1A/RGN/DIS3L/DFFB/CARHSP1/EXOSC6/CIRBP/CIRBP/FEN1 |
| 11 | 0.515647 | 1.808493 | 0.009266 | 0.331141 | 0.317046 | 492 | tags=80%, list=37%, signal=51% | RPS2/RPL35/RPS9/RPS14/RPS2/RPS2/RPL13A/RPS5/PMM1/RPL18A/RPS14/RPL22 |
| 23 | 0.401394 | 1.891731 | 0.009328 | 0.331141 | 0.317046 | 637 | tags=86%, list=48%, signal=46% | SF1/RPS2/LUC7L3/RPL35/RPS14/RPS2/FUS/RBM4/CSTF3/PPP1R8/RPS2/RPL13A/RPS5/FXR1/RBM38/CIRBP/LARP1/RPS14/SRRM2/CARHSP1/RBM5/CIRBP/CIRBP/PPIE |
| 100 | 0.249632 | 1.801856 | 0.009928 | 0.343396 | 0.328779 | 300 | tags=38%, list=22%, signal=32% | E4F1/MCM8/CDK16/ZNF503/NABP2/GOLGA8A/UNC119/SYCP2/AKT1/AKT2/SGSM3/SOX2/UBD/CCNF/ARF1/MCM8/ACTR1A/PKD1/GPER1/LIG1/MDM4/OFD1/ACTR1A/NCAPH2/PSMC4/MAD2L2/CDK5RAP3/GIT1/NSFL1C/MAX/ILK/TUBGCP3/MGA/PPME1/SOX4/AKAP9/PSMB3/NAA10/CDC42/NSFL1C/BIRC5 |
| 20 | 0.411764 | 1.843639 | 0.010192 | 0.343728 | 0.329097 | 513 | tags=71%, list=38%, signal=44% | RPS2/AKT2/RPL35/RPS9/RPS14/RILPL2/RPS2/PPP1R13B/RPS2/RPL13A/RPS5/MIEF1/RPL18A/GRIPAP1/RPS14/RPL22/MYO1C |
| 51 | 0.295555 | 1.793901 | 0.010558 | 0.347373 | 0.332587 | 622 | tags=66%, list=47%, signal=37% | PAIP2B/HABP4/RPS2/AKT1/EIF4B/AKT2/RPL35/NARS2/RPS9/RPS14/RPS2/RBM4/EIF4B/LARP4/CNDP2/SOX4/EEF1D/MRPL1/MRPL11/TUFM/EEF1D/RPS2/RPL13A/RPS5/FXR1/CIRBP/MTG2/RPL18A/RCC1L/LARP1/RPS14/RPL22/PINK1/MTRF1/CDKAL1/CDK5RAP1/EEF1A1/RPS6KB2/CIRBP/CIRBP |
| 90 | -0.13923 | -1.83347 | 0.011356 | 0.357324 | 0.342115 | 723 | tags=74%, list=54%, signal=36% | ATP1A3/ITGAL/ITGA5/SLC6A13/FOLR1/SLC25A11/P2RY8/PALM/LTB4R2/CLDN3/HLA-DRB3/ERBB3/OBSCN/ULBP2/HLA-DRB1/NOS1AP/SLC2A10/CD6/GRIA4/ADRA2C/ADGRG3/NCR3/OPN1SW/SLC5A6/LILRA4/CHRNE/STRA6/SSTR5/PSEN1/IGF2R/ULBP2/SLC39A2/IL27RA/FXYD2/EPHB3/FXYD6/TMC2/TNFSF11/GALR2/SEMA3D/EREG/SCN3A/FOLR3/LRFN1/APLNR/FGFR4/SYT7/EFNA5/STAB2/KCNH6/CSPG5/TNF/CNTN2/ABHD17A/KCNQ3/ADCY7/SCNN1B/SLC26A6/LIFR/SEMA6A/ADCY5/GJD3/KCNA7/LGR4/MMP24/ADCY9/NPFFR1/EDA2R/SLC6A20/COL17A1 |
| 73 | 0.264833 | 1.775587 | 0.011526 | 0.357324 | 0.342115 | 521 | tags=55%, list=39%, signal=36% | E4F1/MAGEA2B/POM121/POM121/RPS2/SOCS7/AKT1/PPIL2/WWP2/UBD/CCNF/KLHL42/USP10/TRIM62/MDM4/RPS2/GNL3L/DCAF7/PSMC4/USP22/MAD2L2/CDK5RAP3/AKTIP/SOX4/DCAF8/PSMB3/CDC42/MED10/DDA1/RPS2/MIB2/NFE2L1/UBE2D4/NUP210/URM1/TRIM28/MCRS1/NUP93/RNF121/BCL11A/UBXN1/USP5/HSPA1A/PINK1 |
| 109 | 0.23275 | 1.703383 | 0.011655 | 0.357324 | 0.342115 | 549 | tags=51%, list=41%, signal=33% | SP5/SP5/E4F1/PAIP2B/MAGEA2B/MAGEA1/SF1/ZNF503/CTBP1/MST1/ASCL2/SOX2/WWP2/SMARCA2/GHRL/ZNF337/CIITA/GPER1/VGLL4/RPS14/MDM4/MPHOSPH8/ATF7IP/GNL3L/ZNF764/RBM4/MAD2L2/MAX/MGA/JAK3/BIRC5/CTNS/SIRPA/ATN1/RPL13A/HES5/FXR1/SCML1/NFIC/CIRBP/HOXB4/BMP7/TRIM28/MFSD12/LARP1/SAP130/KDM2A/SCMH1/RPS14/ZMYND8/WWOX/KLF7/BCL11A/SPEN/E2F2/HSPA1A/SMAD9/RGN/MAGED1/MTA3/CHD8 |
| 22 | 0.404194 | 1.866348 | 0.012437 | 0.372822 | 0.356953 | 532 | tags=70%, list=40%, signal=43% | ACOT7/GSTM4/VTN/GSTM2/ACOT11/PNPLA3/FN1/F2/ACACB/HADHA/AGRN/BMP7/GSTM1/NAV2/RPL22/FGF7 |
| 142 | 0.217778 | 1.664199 | 0.012934 | 0.379295 | 0.36315 | 544 | tags=53%, list=41%, signal=35% | CD74/TOM1/CPSF4/MCM8/POM121/POM121/RPS2/NABP2/PTPRU/AKT1/AKT2/RHOQ/THOC1/SGSM3/PPIL2/RPL35/ARF1/MCM8/PKD1/GPER1/CCT7/SNX12/RPS9/RPS14/RILPL2/RPS2/NUTF2/GOLGB1/WDR33/GNL3L/CDK5RAP3/NSFL1C/F2/CDC42/NSFL1C/PPP1R13B/LSG1/TMED9/BCAP31/STX4/RPS2/SEC13/PSAP/RPL13A/SZT2/ITGAL/RPS5/MIEF1/STXBP1/NUDT19/SNUPN/ATP13A2/PMM1/NUP210/AGRN/SNX17/ARL2/BMP7/TRIM28/GPHN/RPL18A/MCRS1/APPBP2/CEP192/DHRS4/GRIPAP1/AP2S1/NUP93/SEC23B/RPS14/ZMYND8/RPL22/MYO1C/CNPY4/PINK1/SYNGR1/SORBS1/FGF7/TCP1/TOMM40/MAGED1 |
| 60 | 0.27874 | 1.76959 | 0.013695 | 0.393085 | 0.376353 | 646 | tags=67%, list=48%, signal=36% | PAIP2B/HABP4/RPS2/AKT1/EIF4B/AKT2/RPL35/PDPR/ACLY/NARS2/RPS9/RPS14/RPS2/RBM4/EIF4B/LARP4/CNDP2/SOX4/EEF1D/MRPL1/MRPL11/TUFM/EEF1D/RPS2/PDPR/RPL13A/RPS5/ACACB/FXR1/CIRBP/MTG2/ACSBG1/RPL18A/RCC1L/LARP1/RPS14/RPL22/PDP2/PINK1/MTRF1/CDKAL1/CDK5RAP1/EEF1A1/RPS6KB2/CIRBP/CIRBP/ASAH1/SIRT3 |
| 15 | 0.463335 | 1.860806 | 0.014152 | 0.397732 | 0.380802 | 460 | tags=65%, list=34%, signal=43% | HMGCS2/GPAT2/CIITA/ACLY/USP22/MBOAT7/PNPLA3/NAA10/HADHA/MCRS1/DLST |
| 45 | 0.304197 | 1.782891 | 0.014646 | 0.403221 | 0.386058 | 310 | tags=43%, list=23%, signal=34% | E4F1/NABP2/AKT2/THOC1/SGSM3/SOX2/CCNF/PKD1/GPER1/MDM4/PSMC4/MAD2L2/CDK5RAP3/MAX/ILK/MGA/SOX4/PSMB3/NAA10/PPP1R13B |
| 21 | 0.393064 | 1.799747 | 0.015964 | 0.427199 | 0.409015 | 733 | tags=83%, list=55%, signal=38% | HP1BP3/MCM8/MCM8/GPER1/MPHOSPH8/ATF7IP/NCAPH2/NAA10/CABIN1/CENPN/TRIM28/NAV2/CHD8/DFFB/DDX11/HMGB3/RUVBL1/CENPO/MCM2/CHMP1A |
| 13 | 0.466529 | 1.770533 | 0.01637 | 0.427199 | 0.409015 | 492 | tags=76%, list=37%, signal=49% | RPS2/RPL35/RPS9/RPS14/RPS2/BCAP31/RPS2/RPL13A/RPS5/PMM1/RPL18A/RPS14/RPL22 |
| 31 | -0.24325 | -1.71176 | 0.016653 | 0.427199 | 0.409015 | 718 | tags=84%, list=54%, signal=40% | ITGA5/P4HB/FOLR1/FLOT1/ULBP2/HLA-DRB1/CD6/MICB/AZGP1/NCAM1/ULBP2/IL27RA/BTN2A3P/CXCR1/FOLR3/EFNA5/STAB2/TNF/CD3D/SCNN1B/LIFR/BTNL3/ICOSLG/SCUBE1/FGB/ANTXR2/BTNL8 |
| 35 | 0.319799 | 1.736961 | 0.016784 | 0.427199 | 0.409015 | 618 | tags=71%, list=46%, signal=39% | CPSF4/RPS2/RPL7L1/DDX17/RPL35/RPS9/RPS14/RPS2/TRMT1/NSUN5P2/DDX51/MRPL1/EMG1/TRMT6/RPS2/URM1/DIS3/WDR74/NHP2/THADA/RPS14/METTL15/CDKAL1/CDK5RAP1/EXOSC6/METTL1/ADAT1/WDR46/ADAT1 |
| 21 | 0.387928 | 1.776233 | 0.017241 | 0.430714 | 0.41238 | 481 | tags=64%, list=36%, signal=42% | CD74/HLA-DQA1/HLA-DQA1/ARF1/ACTR1A/ACTR1A/HLA-DQA2/HLA-DQB1/HLA-DRB5/DYNC1LI2/HLA-DMA/SEC13/DCTN3/KLC1/HLA-DRB4/AP2S1 |
| 20 | 0.395565 | 1.771109 | 0.018233 | 0.445299 | 0.426344 | 385 | tags=60%, list=29%, signal=43% | MST1/AKT1/AKT2/PKD1/MAPKAPK2/SPOCK2/MAD2L2/ILK/AKAP9/NAA10/PRKX/STK38 |
| 28 | 0.364949 | 1.850882 | 0.018485 | 0.445299 | 0.426344 | 481 | tags=62%, list=36%, signal=41% | CD74/HLA-DQA1/HLA-DQA1/ARF1/ACTR1A/ACTR1A/PSMC4/HLA-DQA2/PSMB3/HLA-DQB1/HLA-DRB5/DYNC1LI2/HLA-DMA/BCAP31/SEC13/DCTN3/KLC1/HLA-DRB4/TAPBP/AP2S1 |
| 21 | 0.381427 | 1.746466 | 0.019619 | 0.464305 | 0.444541 | 535 | tags=71%, list=40%, signal=43% | AKT1/PDCL3/PKD1/VGLL4/PSMC4/CDK5RAP3/BCAP31/DDA1/NFE2L1/ATP13A2/UBXN1/USP5/HSPA1A/PINK1/RGN |
| 22 | 0.385319 | 1.779193 | 0.020182 | 0.469408 | 0.449427 | 381 | tags=50%, list=29%, signal=36% | HLA-DQA1/HLA-DQA1/CX3CL1/UBD/TRIM62/CIITA/HLA-DQA2/HLA-DQB1/HLA-DRB5/CDC42/STX4/SIRPA/RPL13A/STXBP1 |
| 81 | 0.247976 | 1.700248 | 0.021814 | 0.498773 | 0.477542 | 680 | tags=65%, list=51%, signal=34% | ITGB8/HP1BP3/CD74/ESPNL/MCM8/CX3CL1/MYH3/EIF4B/UBD/MCM8/CIITA/VTN/SYNE2/GNAS/FMNL1/PPP5C/EIF4B/GIT1/MAX/ILK/FN1/KRAS/CIB2/CAMSAP1/SNRPA/NAA10/IGF2R/HLA-DMA/ARPC1B/BCAP31/ITGAL/NFE2L1/PQBP1/CABIN1/HADHA/ANTXR1/MYH9/LARP1/TAPBP/ATG101/UBXN1/MYO1C/PINK1/SORBS1/MTA3/PI4K2A/CDK5RAP1/TAB1/ITGAL/ITGA5/P4HB/AMFR/VPS33B/GCHFR/RELL2/GNB3/RUVBL1 |
| 37 | 0.315481 | 1.742822 | 0.022493 | 0.505713 | 0.484187 | 346 | tags=45%, list=26%, signal=35% | CD74/FUT1/HLA-DQA1/HLA-DQA1/GOLGA8A/ARF1/VPS52/GPER1/GOLGB1/NSFL1C/HLA-DQA2/GPR89B/AKAP9/HLA-DQB1/HLA-DRB5/NSFL1C/IGF2R/BCAP31/STX4 |
| 19 | 0.397815 | 1.746535 | 0.023379 | 0.506321 | 0.484769 | 492 | tags=71%, list=37%, signal=46% | PAIP2B/HABP4/RPS2/EIF4B/RPL35/RPS9/RPS14/RPS2/RBM4/EIF4B/RPS2/RPL13A/RPS5/RPL18A/LARP1/RPS14/RPL22 |
| 13 | 0.450697 | 1.710448 | 0.024127 | 0.506321 | 0.484769 | 622 | tags=75%, list=47%, signal=41% | CPSF4/SF1/DDX17/RBM42/RBM4/CDK11B/SNRPA/FXR1/CIRBP/RBM5/CIRBP/CIRBP |
| 13 | -0.35631 | -1.60055 | 0.024162 | 0.506321 | 0.484769 | 463 | tags=71%, list=35%, signal=47% | KRTAP10-10/ZBED2/EREG/KRTAP1-1/LCE2A/KRT13/KRTAP5-8/SHARPIN/GRHL1/LCE1F |
| 11 | 0.47082 | 1.651276 | 0.024465 | 0.506321 | 0.484769 | 131 | tags=45%, list=10%, signal=41% | XBP1/MST1/AKT1/AKT2/STAT5B |
| 11 | 0.472586 | 1.657469 | 0.024465 | 0.506321 | 0.484769 | 243 | tags=55%, list=18%, signal=45% | SF1/CX3CL1/AKT1/GPER1/VGLL4/ILK |
| 42 | 0.300161 | 1.72202 | 0.025002 | 0.506321 | 0.484769 | 618 | tags=67%, list=46%, signal=38% | CPSF4/RPS2/GPAT2/RPL7L1/DDX17/RPL35/NARS2/RPS9/RPS14/RPS2/TRMT1/NSUN5P2/DDX51/MRPL1/EMG1/TRMT6/RPS2/URM1/DIS3/WDR74/NHP2/THADA/RPS14/METTL15/DIS3L/CDKAL1/CDK5RAP1/EXOSC6/MYBL1/METTL1/ADAT1/WDR46/ADAT1 |
| 37 | 0.311172 | 1.719016 | 0.025199 | 0.506321 | 0.484769 | 460 | tags=56%, list=34%, signal=38% | HMGCS2/FMOD/ACOT7/GSTM4/STAT5B/PDPR/ACLY/GSTM2/XYLT2/SPOCK2/CIAO1/ACOT11/CNDP2/ABHD14B/SLC25A19/CTNS/PDPR/NFE2L1/ACACB/ACSBG1/GSTM1/DLST |
| 22 | 0.374472 | 1.729106 | 0.025737 | 0.506321 | 0.484769 | 707 | tags=83%, list=53%, signal=40% | CPSF4/POM121/POM121/THOC1/CCT7/NUTF2/WDR33/LSG1/SEC13/SNUPN/NUP210/NHP2/NUP93/MYO1C/TCP1/RUVBL1/DDX19B/RANBP17/FLOT1/THOC5 |
| 30 | 0.337401 | 1.753701 | 0.025898 | 0.506321 | 0.484769 | 778 | tags=86%, list=58%, signal=37% | CD74/HLA-DQA1/POM121/POM121/HLA-DQA1/ACOT7/FKBP5/GSTM4/GSTM2/ANPEP/ACOT11/PNPLA3/HLA-DQB1/HLA-DRB5/PSAP/ACACB/HADHA/GSTM1/TAPBP/HLA-DPB1/RPS6KB2/ATP1A3/PPIE/HSPG2/FOLR1/HLA-DRB5/PSAP/HLA-DRB3/HLA-DRB1/GRIA4 |
| 13 | 0.444936 | 1.688585 | 0.02652 | 0.509204 | 0.487529 | 521 | tags=80%, list=39%, signal=49% | MAGEA2B/RPS2/AKT1/RPS2/GNL3L/MAD2L2/CDK5RAP3/SOX4/RPS2/UBXN1/HSPA1A/PINK1 |
| 23 | -0.28445 | -1.79324 | 0.027702 | 0.509204 | 0.487529 | 580 | tags=79%, list=43%, signal=46% | IFT140/SMO/FOXJ1/HEY2/PBX3/PSEN1/TCTN1/ZBTB16/PAX8/HOXA4/CDX1/TCF15/DLX2/FOXJ1/MESP1/HOXC5/MDFI/CHRD/HES6 |
| 11 | 0.465564 | 1.632841 | 0.027811 | 0.509204 | 0.487529 | 332 | tags=58%, list=25%, signal=44% | XBP1/PRKCSH/PKD1/VTN/CDK5RAP3/KRAS/IGF2R |
| 113 | 0.218932 | 1.615831 | 0.028513 | 0.509204 | 0.487529 | 684 | tags=62%, list=51%, signal=33% | MCM8/CDK16/PRPS1/MYH3/PI4KB/ACOT7/COASY/NME4/SPG7/AKT1/SKIV2L/AKT2/PIP4K2A/DDX17/BMPR1B/SMARCA2/MCM8/ACTR1A/MAPKAPK2/CIITA/ACLY/NARS2/TTF2/CCT7/LIG1/ACTR1A/PSMC4/UCKL1/ACOT11/CDK11B/PPP5C/ILK/PNPLA3/DNAH3/DDX51/JAK3/SLC27A3/DGKZ/DYNC1LI2/MIEF1/PRKX/STK38/ACACB/UBE2D4/ATP13A2/HADHA/MYH9/GPHN/ACSBG1/KIF21B/NAV2/SCN8A/HSPA1A/MYO1C/PINK1/DDX31/TCP1/MAP3K4/CHD8/PTK7/PI4K2A/DCLK2/RPS6KB2/FLAD1/FLAD1/DDX11/ATP1A3/LONP1/ARAF/PMVK/MMAB/FN3K/UBA7/RUVBL1/DDX19B |
| 140 | 0.204285 | 1.560157 | 0.029263 | 0.509204 | 0.487529 | 689 | tags=62%, list=52%, signal=34% | MCM8/CDK16/PRPS1/MYH3/PI4KB/ACOT7/COASY/NME4/SPG7/AKT1/SKIV2L/AKT2/RHOQ/PIP4K2A/DDX17/BMPR1B/ARF1/SMARCA2/MCM8/ACTR1A/MAPKAPK2/CIITA/ACLY/NARS2/TTF2/CCT7/LIG1/ACTR1A/GNL3L/PSMC4/UCKL1/GNAS/ACOT11/CDK11B/PPP5C/ILK/PNPLA3/DNAH3/DDX51/KRAS/JAK3/SLC27A3/DGKZ/CDC42/TUFM/LSG1/DYNC1LI2/MIEF1/PRKX/STK38/ACACB/NIN/UBE2D4/TUBA3D/ATP13A2/HADHA/ARL2/MTG2/MYH9/GPHN/ACSBG1/KIF21B/RCC1L/NAV2/SCN8A/HSPA1A/MYO1C/PINK1/DDX31/TCP1/MAP3K4/CHD8/PTK7/PI4K2A/DCLK2/EEF1A1/RPS6KB2/FLAD1/FLAD1/DDX11/ATP1A3/LONP1/ARAF/NDUFV1/PMVK/MMAB/FN3K/UBA7/RUVBL1/DDX19B/RANBP17 |
| 11 | -0.41466 | -1.72547 | 0.02934 | 0.509204 | 0.487529 | 261 | tags=73%, list=20%, signal=59% | MAVS/RBCK1/ADAR/TNF/SHARPIN/LIFR/PYDC1/CASP8 |
| 40 | 0.306229 | 1.725197 | 0.030337 | 0.509204 | 0.487529 | 546 | tags=60%, list=41%, signal=36% | BCL9/RALY/SF1/CTBP1/DDX17/SMARCA2/SUPT20H/CIITA/ATF7IP/FUS/PMF1/USP22/MED13L/SOX4/MED10/ATN1/PQBP1/TRIM28/ZMYND8/WWOX/BCL11A/SPEN/HSPA1A/MAGED1/MTA3 |
| 24 | -0.27079 | -1.74934 | 0.030764 | 0.509204 | 0.487529 | 981 | tags=100%, list=73%, signal=27% | HES5/KLK5/KLF7/FGF7/ASAH1/PSAP/HLA-DRB1/SMO/HEY2/KRTAP10-10/ZBED2/EREG/KRTAP1-1/LHFPL5/LCE2A/TNF/KRT13/KRTAP5-8/SHARPIN/GRHL1/LGR4/LCE1F/EDA2R/COL17A1 |
| 41 | 0.301594 | 1.715666 | 0.031355 | 0.509204 | 0.487529 | 383 | tags=44%, list=29%, signal=33% | CX3CL1/AKT1/BMPR1B/GHRL/STAT5B/MAPKAPK2/CIITA/GPER1/CXCR3/CXCL5/APOL3/FN1/F2/OGG1/PGLYRP1/TIRAP/SAA2/SIRPA/ITGAL/NFE2L1 |
| 16 | 0.402726 | 1.66381 | 0.031766 | 0.509204 | 0.487529 | 494 | tags=71%, list=37%, signal=45% | FMOD/GSTM4/PDPR/ACLY/GSTM2/XYLT2/CNDP2/PDPR/ACACB/ACSBG1/GSTM1/PDP2 |
| 29 | 0.331279 | 1.701391 | 0.032407 | 0.509204 | 0.487529 | 768 | tags=87%, list=58%, signal=38% | MCM8/MYH3/SKIV2L/DDX17/SMARCA2/MCM8/TTF2/ATF7IP/PSMC4/DNAH3/DDX51/ATP13A2/MYH9/KIF21B/NAV2/HSPA1A/MYO1C/DDX31/CHD8/DDX11/LONP1/RUVBL1/DDX19B/MCM2/DDX56/EP400 |
| 103 | 0.222458 | 1.616477 | 0.032787 | 0.509204 | 0.487529 | 326 | tags=35%, list=24%, signal=29% | HP1BP3/CD74/SF1/FUT1/ZNF503/CX3CL1/MALAT1/XBP1/CTBP1/PTPRU/MST1/AKT1/AKT2/ASCL2/BMPR1B/SOX2/SMARCA2/GHRL/STAT5B/PDCL3/GPER1/VGLL4/RPS9/MDM4/CXCL5/CIAO1/ILK/FN1/KRAS/SOX4/JAK3/F2/CDC42/BIRC5/DLG5/WDR13/TIRAP |
| 14 | 0.425649 | 1.662044 | 0.032852 | 0.509204 | 0.487529 | 322 | tags=53%, list=24%, signal=41% | CD74/CX3CL1/THOC1/GPER1/JAK3/F2/DLG5/PGLYRP1 |
| 14 | 0.424678 | 1.658253 | 0.032852 | 0.509204 | 0.487529 | 774 | tags=100%, list=58%, signal=43% | MCM8/MCM8/PDCL3/CCT7/MDM4/ATF7IP/SOX4/STXBP1/HSPA1A/PINK1/TCP1/FLOT1/UBE2B/DNLZ/SMO |
| 19 | 0.371086 | 1.629186 | 0.03287 | 0.509204 | 0.487529 | 263 | tags=45%, list=20%, signal=37% | E4F1/AKT2/SGSM3/SOX2/PKD1/GPER1/MDM4/ILK/SOX4 |
| 59 | 0.259616 | 1.643796 | 0.033049 | 0.509204 | 0.487529 | 300 | tags=35%, list=22%, signal=29% | CD74/HLA-DQA1/HLA-DQA1/CX3CL1/AKT1/SOX2/ARF1/STAT5B/TRIM62/CIITA/CXCR3/CXCL5/PSMC4/ILK/FN1/HLA-DQA2/KRAS/JAK3/PSMB3/HLA-DQB1/HLA-DRB5/CDC42/BIRC5 |
| 59 | 0.25887 | 1.639076 | 0.033049 | 0.509204 | 0.487529 | 346 | tags=40%, list=26%, signal=31% | CD74/CX3CL1/MALAT1/XBP1/MST1/AKT1/AKT2/GHRL/STAT5B/PDCL3/GPER1/RPS9/CXCL5/CIAO1/ILK/FN1/KRAS/SOX4/JAK3/F2/CDC42/BIRC5/TIRAP/STX4 |
| 15 | 0.43145 | 1.73275 | 0.033088 | 0.509204 | 0.487529 | 513 | tags=74%, list=38%, signal=46% | RPS2/AKT2/RPL35/RPS9/RPS14/RPS2/RPS2/RPL13A/RPS5/MIEF1/RPL18A/RPS14/RPL22/MYO1C |
| 22 | 0.366517 | 1.692375 | 0.033513 | 0.509204 | 0.487529 | 613 | tags=74%, list=46%, signal=41% | RPS2/RPL7L1/DDX17/RPL35/RPS9/RPS14/RPS2/NSUN5P2/DDX51/MRPL1/EMG1/RPS2/DIS3/WDR74/NHP2/RPS14/METTL15/DIS3L/EXOSC6/WDR46 |
| 41 | 0.298338 | 1.697141 | 0.033595 | 0.509204 | 0.487529 | 272 | tags=43%, list=20%, signal=36% | MCM8/CDK16/AKT1/UBD/CCNF/MCM8/ACTR1A/PKD1/MDM4/OFD1/ACTR1A/PSMC4/MAD2L2/CDK5RAP3/MAX/PPME1/SOX4/AKAP9/PSMB3 |
| 17 | 0.387811 | 1.636013 | 0.034615 | 0.518846 | 0.496761 | 707 | tags=84%, list=53%, signal=40% | CPSF4/POM121/POM121/THOC1/NUTF2/WDR33/LSG1/SEC13/SNUPN/NUP210/NUP93/MYO1C/DDX19B/RANBP17/FLOT1/THOC5 |
| 34 | 0.30589 | 1.643476 | 0.035469 | 0.522514 | 0.500273 | 535 | tags=60%, list=40%, signal=37% | AKT1/SGSM3/WWP2/PDCL3/PKD1/VGLL4/SNX12/MDM4/ATG4B/PSMC4/MAD2L2/CDK5RAP3/BCAP31/DDA1/NFE2L1/ATP13A2/UBXN1/USP5/HSPA1A/PINK1/RGN |
| 43 | 0.289704 | 1.671949 | 0.035635 | 0.522514 | 0.500273 | 595 | tags=62%, list=45%, signal=35% | HMGCS2/GPAT2/PRPS1/PI4KB/ACOT7/COASY/NME4/PIP4K2A/ARF1/PDPR/ACLY/GPER1/UCKL1/MBOAT7/PNPLA3/DGKZ/PIGX/PDPR/ACACB/MTMR1/SACM1L/GPHN/ACSBG1/PDP2/PLA2G2E/PINK1/PI4K2A/FLAD1/FLAD1 |
| 17 | -0.29266 | -1.56493 | 0.036728 | 0.532245 | 0.50959 | 506 | tags=76%, list=38%, signal=48% | CES1/KLK3/PSEN1/ANGPTL8/AFG3L2/FKRP/BAD/CNTN2/AEBP1/KLK2/CASP8/FGB/SPON1 |
| 18 | 0.383177 | 1.644223 | 0.037323 | 0.532245 | 0.50959 | 410 | tags=55%, list=31%, signal=39% | HMGCS2/GPAT2/PI4KB/PIP4K2A/ARF1/MBOAT7/PNPLA3/DGKZ/PIGX/MTMR1/SACM1L |
| 16 | 0.395141 | 1.632475 | 0.038119 | 0.532245 | 0.50959 | 483 | tags=67%, list=36%, signal=43% | CPSF4/POM121/POM121/THOC1/WDR33/BIRC5/LSG1/SEC13/NUP210/GPHN/GRIPAP1/NUP93 |
| 49 | 0.271686 | 1.631001 | 0.038504 | 0.532245 | 0.50959 | 492 | tags=56%, list=37%, signal=37% | FMOD/POM121/POM121/RPS2/RPL7L1/COL9A3/RPL35/RPS9/RPS14/RPS2/NUTF2/VTN/CLDN4/TUBGCP3/FN1/MRPL1/CRYBB2/MRPL11/ARPC1B/RPS2/SEC13/RPL13A/RPS5/BFSP1/TUBA3D/DCTN3/AGRN/RPL18A/NUP93/RPS14/RPL22 |
| 16 | 0.40871 | 1.688536 | 0.039548 | 0.532245 | 0.50959 | 146 | tags=41%, list=11%, signal=37% | EYA2/CD74/EYA2/CX3CL1/XBP1/AKT1/NDUFS3 |
| 17 | 0.383503 | 1.617838 | 0.039744 | 0.532245 | 0.50959 | 264 | tags=42%, list=20%, signal=34% | CD74/ZNF683/XBP1/STAT5B/NCAPH2/GPR89B/SOX4/JAK3 |
| 106 | 0.219544 | 1.598718 | 0.039837 | 0.532245 | 0.50959 | 549 | tags=50%, list=41%, signal=32% | SP5/SP5/E4F1/MAGEA2B/MAGEA1/SF1/ZNF503/CTBP1/THOC1/ASCL2/SOX2/WWP2/SMARCA2/ZNF337/MAPKAPK2/CIITA/GPER1/RBM42/VGLL4/RPS14/MDM4/MPHOSPH8/ATF7IP/FUS/GNL3L/ZNF764/MAD2L2/MAX/MGA/OGG1/BIRC5/TIRAP/ATN1/NBAS/HES5/SCML1/NFIC/CIRBP/HOXB4/BMP7/TRIM28/LARP1/SAP130/KDM2A/SCMH1/RPS14/ZMYND8/WWOX/KLF7/BCL11A/SPEN/E2F2/HSPA1A/SMAD9/RGN/MAGED1/MTA3/CHD8 |
| 15 | 0.409886 | 1.646147 | 0.039897 | 0.532245 | 0.50959 | 733 | tags=82%, list=55%, signal=38% | HP1BP3/GPER1/MPHOSPH8/ATF7IP/NCAPH2/NAA10/CABIN1/CENPN/TRIM28/DFFB/RUVBL1/CENPO/MCM2/CHMP1A |
| 18 | -0.31323 | -1.73711 | 0.040228 | 0.532245 | 0.50959 | 611 | tags=83%, list=46%, signal=46% | ENOSF1/CBS/APMAP/GGCX/CA12/TKFC/FAHD2A/CSAD/CA5BP1/SCLY/ADCY7/NAXD/ADCY5/ECHDC2/ADCY9 |
| 21 | 0.346379 | 1.585987 | 0.040244 | 0.532245 | 0.50959 | 622 | tags=70%, list=47%, signal=38% | AKT1/PDCL3/MAPKAPK2/FUS/CDK5RAP3/TIRAP/NBAS/ACACB/ATP13A2/CIRBP/LARP1/UBXN1/PINK1/RGN/CIRBP/CIRBP |
| 54 | 0.261026 | 1.615428 | 0.04126 | 0.537032 | 0.514173 | 622 | tags=61%, list=47%, signal=34% | PAIP2B/POM121/POM121/HABP4/AKT1/EIF4B/AKT2/MAPKAPK2/RPS9/RPS14/FUS/PSMC4/RBM4/EIF4B/LARP4/SOX4/PSMB3/TIRAP/SEC13/RPL13A/NUP210/FXR1/DIS3/CIRBP/MTG2/RCC1L/LARP1/NUP93/RPS14/HSPA1A/PINK1/MTRF1/CARHSP1/CDK5RAP1/EXOSC6/RPS6KB2/CIRBP/CIRBP |
| 19 | 0.375764 | 1.649726 | 0.042404 | 0.537032 | 0.514173 | 622 | tags=76%, list=47%, signal=41% | AKT1/MAPKAPK2/FUS/PSMC4/PSMB3/TIRAP/NBAS/FXR1/DIS3/CIRBP/LARP1/HSPA1A/CARHSP1/EXOSC6/CIRBP/CIRBP |
| 13 | 0.433001 | 1.64329 | 0.04244 | 0.537032 | 0.514173 | 439 | tags=69%, list=33%, signal=47% | ITPR2/GNAS/ILK/FN1/F2/DGKZ/FIBP/STXBP1/MYH9 |
| 13 | 0.430408 | 1.633451 | 0.04244 | 0.537032 | 0.514173 | 825 | tags=100%, list=62%, signal=39% | HMGCS2/ACOT7/PDPR/ACLY/ACOT11/PDPR/ACACB/ACSBG1/DLST/PDP2/AASS/PMVK/OGDH/ACSBG2/OGDH |
| 117 | 0.205763 | 1.530388 | 0.042596 | 0.537032 | 0.514173 | 492 | tags=47%, list=37%, signal=33% | CD74/TOM1/CPSF4/POM121/POM121/RPS2/SPG7/AKT1/AKT2/THOC1/SGSM3/RPL35/ARF1/ACTR1A/VPS52/PKD1/SNX12/RPS9/RPS14/RILPL2/RPS2/NUTF2/GOLGB1/WDR33/ACTR1A/SYNE2/RBM4/AKTIP/F2/CDC42/PPP1R13B/LSG1/DYNC1LI2/TMED9/BCAP31/STX4/RPS2/SEC13/PSAP/RPL13A/RPS5/MIEF1/STXBP1/ACACB/NUDT19/SNUPN/ATP13A2/PMM1/NUP210/DCTN3/SNX17/TRIM28/RPL18A/APPBP2/DHRS4/GRIPAP1/AP2S1/NUP93/SEC23B/RPS14/RPL22 |
| 16 | 0.390504 | 1.613316 | 0.043202 | 0.53775 | 0.51486 | 370 | tags=53%, list=28%, signal=39% | CD74/ZNF683/XBP1/THOC1/APBB1IP/MAD2L2/JAK3/PGLYRP1/ITGAL |
| 10 | 0.458889 | 1.537928 | 0.043909 | 0.53775 | 0.51486 | 269 | tags=60%, list=20%, signal=48% | ITGB8/CX3CL1/VTN/ILK/FN1/CIB2 |
| 16 | 0.389005 | 1.607123 | 0.044473 | 0.53775 | 0.51486 | 768 | tags=94%, list=58%, signal=40% | MCM8/SKIV2L/DDX17/SMARCA2/MCM8/TTF2/DDX51/NAV2/DDX31/CHD8/DDX11/RUVBL1/DDX19B/MCM2/DDX56/EP400 |
| 10 | -0.39849 | -1.5618 | 0.044731 | 0.53775 | 0.51486 | 808 | tags=100%, list=61%, signal=40% | MEN1/KMT2C/KMT2A/BCOR/NTMT1/MLLT6/EHMT2/ZNF304/PRMT7 |
| 15 | 0.40615 | 1.631142 | 0.045045 | 0.53775 | 0.51486 | 485 | tags=61%, list=36%, signal=39% | XIST/POM121/POM121/CTBP1/DDX17/RBM4/SEC13/NUP210/FXR1/NUP93/SCMH1 |
| 18 | 0.37415 | 1.605489 | 0.045045 | 0.53775 | 0.51486 | 679 | tags=83%, list=51%, signal=42% | CD74/PDRG1/FKBP5/PRKCSH/PPIL2/PDCL3/CCT7/UBXN1/HSPA1A/TCP1/TBCD/PPIE/P4HB/AMFR/GNB3 |
| 10 | 0.457523 | 1.533351 | 0.046742 | 0.549287 | 0.525906 | 347 | tags=60%, list=26%, signal=45% | CX3CL1/GHRL/GPER1/F2/PGLYRP1/SIRPA |
| 47 | 0.272369 | 1.614421 | 0.047514 | 0.549287 | 0.525906 | 845 | tags=84%, list=63%, signal=32% | SOCS7/PRPS1/WWP2/CCNF/KLHL42/SUPT20H/DCAF7/USP22/RABGGTA/MAD2L2/MAX/MGA/DCAF8/NAA10/TRMT6/MED10/DDA1/MIB2/UBE2D4/MCRS1/DLST/ZNF335/CHD8/ASB2/ZSWIM8/METTL1/GID4/MEN1/ING4/AMFR/RUVBL1/OGDH/KLHL22/UBE2B/KMT2C/FBXO44/EP400/ZER1/KMT2A/OGDH/SNRPB |
| 45 | 0.280623 | 1.644723 | 0.047566 | 0.549287 | 0.525906 | 527 | tags=58%, list=39%, signal=36% | RPS2/AKT1/AKT2/RHOQ/PPIL2/RPL35/GPER1/RPS9/RPS14/RILPL2/RPS2/PPP1R13B/STX4/RPS2/RPL13A/ITGAL/RPS5/MIEF1/STXBP1/AGRN/GPHN/RPL18A/GRIPAP1/RPS14/ZMYND8/RPL22/MYO1C/CNPY4/SORBS1 |
| 41 | 0.285254 | 1.622711 | 0.048152 | 0.549287 | 0.525906 | 581 | tags=59%, list=44%, signal=34% | CD74/TOM1/HLA-DQA1/HLA-DQA1/ACP2/SLC11A2/ANPEP/SPNS1/SLC2A6/HLA-DQA2/HLA-DQB1/HLA-DRB5/CTNS/IGF2R/HLA-DMA/SEC13/PSAP/SZT2/ATP13A2/MFSD12/STARD3/HLA-DRB4/AP2S1/SYNGR1/HLA-DPB1/PI4K2A/EEF1A1 |
| 16 | 0.385284 | 1.591754 | 0.048285 | 0.549287 | 0.525906 | 213 | tags=41%, list=16%, signal=35% | ITGB8/XBP1/BMPR1B/GHRL/GLG1/PKD1/GNAS |
| 11 | 0.452727 | 1.587818 | 0.048679 | 0.549287 | 0.525906 | 264 | tags=50%, list=20%, signal=40% | CD74/ZNF683/XBP1/STAT5B/SOX4/JAK3 |
| 18 | 0.372101 | 1.596698 | 0.048906 | 0.549287 | 0.525906 | 527 | tags=67%, list=39%, signal=41% | AKT1/AKT2/RHOQ/GPER1/PPP1R13B/STX4/MIEF1/GRIPAP1/ZMYND8/MYO1C/CNPY4/SORBS1 |
| 55 | 0.254181 | 1.588866 | 0.049516 | 0.549287 | 0.525906 | 758 | tags=75%, list=57%, signal=34% | MAGEA2B/XBP1/AKT1/WWP2/UBD/CCNF/KLHL42/USP10/PDCL3/PKD1/VGLL4/PSMC4/USP22/CDK5RAP3/NSFL1C/PSMB3/NSFL1C/BCAP31/DDA1/PSAP/NFE2L1/UBE2D4/ATP13A2/AP2S1/RNF121/UBXN1/USP5/HSPA1A/PINK1/RGN/ASB2/GID4/TMUB2/LONP1/ARAF/AMFR/TMUB2/UBA7/UBXN11/KLHL22/PSAP/UBE2B/TMUB2/TGFB1I1/FBXO44 |
| 53 | 0.254183 | 1.569062 | 0.049676 | 0.549287 | 0.525906 | 355 | tags=38%, list=27%, signal=29% | CD74/FUT1/HLA-DQA1/HLA-DQA1/GOLGA8A/PI4KB/ARF1/GLG1/VPS52/PKD1/GPER1/GOLGB1/XYLT2/KIAA0319L/HLA-DQA2/GPR89B/HLA-DQB1/HLA-DRB5/CDC42/IGF2R/TMED9/BCAP31/SEC13 |
